# Prognosis of male lung cancer patients with urinary cancer: a study from a national population-based analysis

**DOI:** 10.1038/s41598-023-27566-8

**Published:** 2023-01-06

**Authors:** Wenqiang Li, Mingliang Cheng, Pei Hu, Liang Jiang, Xiaoping Zhao

**Affiliations:** 1 Center of Stomatology, Tongji Hospital, Tongji Medical College, Huazhong University of Science and Technology, Wuhan, China; 2 Tongji Hospital, Tongji Medical College, Huazhong University of Science and Technology, Wuhan, China

**Keywords:** Cancer epidemiology, Lung cancer, Urological cancer, Scientific data, Statistics

## Abstract

Lung cancer accounts for the most cancer-related deaths in the world. Our previous study suggested the improved survival of lung cancer patients, mainly female patients, with subsequent metachronous primary breast cancer. However, whether the survival advantages of the two primaries are associated with patients’ sex and the specific breast cancer is unclear. Whether male lung cancer patients with another primary may encounter the same survival advantage as female patients is also uncertain. The uncertainty hinders these patients from the potential benefit of lung cancer clinical trial. A total of 343 male lung adenocarcinoma patients with subsequent bladder papillary transitional cell carcinoma (LCBC), 1539 lung adenocarcinoma patients with prior bladder papillary transitional cell carcinoma (BCLC), 1181 lung adenocarcinoma patients with subsequent prostate adenocarcinoma (LCPC), 7426 lung adenocarcinoma patients with prior prostate adenocarcinoma (PCLC), and patients with single bladder/prostate/lung (SLC) cancer were identified from the Surveillance, Epidemiology, and End Results. Patients were classified into simultaneous two primary cancer (sTPC), metachronous two primary cancer 1 (mTPC1), or mTPC2 groups when interval time between two cancers was within 6 months, between 7 and 60 months, or over 60 months, respectively. Propensity matching score program was executed to match the two primary cancers with single primary. Cox regression and competing risk regression were performed to identify confounders associated with all-cause and cancer-specific survival, respectively. The major cancer-related and non-cancer-related death in the two primaries were lung cancer and heart disease, respectively. Median overall survival times since lung primary of LCBC and SLC were 97 and 17 months, respectively, and incidence of all-cause and cancer-specific death in LCBC since lung malignancy was significantly lower (Coef. − 1.24, 95% CI − 1.49 to 0.99; SHR 0.42, 95% CI 0.33–0.53). Among the categorized groups, prognosis values of sTPC and mTPC2 groups were not statically different from that of the matched single lung cancer, whereas increased overall survival time and decreased incidence of all-cause and cancer-specific death relative to the matched patients were observed in mTPC1 group (H.R 0.28, 95% CI 0.19–0.41; SHR 0.33, 95% CI 0.23–0.47). Similar prognosis of LCPC relative to SLC was also observed. Furthermore, a generally improved survival relative to SLC was observed in PCLC (median survival times of PCLC and SLC were 17 and 12 months, respectively; Coef. − 0.32, 95% CI − 0.43 to 0.22; SHR 0.77, 95% CI 0.69–0.85), whereas prognosis of BCLC was similar to the matched ones. These results hinted that survival of lung cancer patients might vary with prior cancer history. Further analysis among groups with the two primaries suggested that advanced bladder cancer was not associated with prognosis of patients with LCBC and BCLC. On the contrary, advanced prostate cancer was associated with all-cause and cancer-specific death in patients with PCLC but not in patients with LCPC. Compared with patients with single lung cancer, male lung cancer patients with subsequent bladder/prostate primary over 6 months experienced generally improved survival. These results were similar to our previous study regarding female lung cancer patients with another breast primary. On the contrary, male lung cancer patients with prior primary malignancy encountered varied prognosis: improved survival relative to single lung primary was observed in lung cancer with prior prostate cancer, whereas prognosis of lung cancer with prior bladder cancer was not different. Therefore, great attention was required to characterize prognosis of lung cancer patients with another primary in advance, which was essential to eliminate the potential bias when these patients were included into the clinical trials.

## Introduction

Great progress has been made in many cancers’ treatment in recent years. However, patients with lung adenocarcinoma still encounter poor prognosis, and these patients may simultaneously/metachronously suffer from another primary cancer^1^. In consideration of multiple cofactors associated with survival, prognosis of the two primaries are poorly known. Moreover, studies targeting lung cancer barely include the two primaries, but some works suggested that risk of potential bias due to the involvement of the two primaries is exaggerated^2–4^. This conclusion is difficult to draw due to the complex influences involved and the lack of population-based evidence. Our previous report suggests that, compared with patients with single lung cancer and lung cancer with prior/subsequent primary breast cancer within 6 months, lung cancer patients, mainly female, with subsequent breast cancer over 6 months encounter improved survival^5^. However, whether the survival advantages of the two primaries is associated with patients’ sex and specific breast cancer is unclear. Whether male lung cancer patients with another primary may encounter the same survival advantage as female patients is also uncertain.

For male patients, the risk of malignancies in urinary system is higher than that in other anatomical sites: incidence of papillary transitional cell carcinoma in bladder (PTCB) and adenocarcinoma in prostate (ACPS) is estimated at 8.6% and 33.10% in 2021^6,7^, respectively. Meanwhile, ratio of PTCB/ACPS in male lung cancer patients with another primary is reported to be higher than that with other malignancies either, which is similar to breast cancer in female lung cancer patients^8^. However, studies focusing on the majority of male two primaries are limited. Missing information of patients’ survival hinders the profound appeal to promote the involvement of these patients into the clinical lung cancer trials.

With data from the Surveillance, Epidemiology, and End Results database of the National Cancer Institute (SEER), this study was designed to depict survival of male lung cancer patients with another bladder/prostate cancer relative to single primary, as well as the cofactors associated with prognosis since lung malignancy.

## Materials and methods

Medical records between January 1975 to December 2018 were identified from SEER by SEER*Stat software version 8.3.5 (accession number: 12223-Nov2018). International Classification of Diseases for Oncology Site Recode (third edition, ICD-O-3), rule of international multiple primary cancers, and SEER historic stage were applied to characterize the anatomical site, records of two primary cancers, and clinical stages (localize, regional, distant, and unknown), respectively. Notably, localized and regional stage of prostate cancer were not distinguished from each other in SEER database. Basic information (race, sex, year and age at diagnosis, insurance, and marital status) and cancer-related information (histology, site, clinical stage, surgical and radiation status, survival time, and cause of death) were selected either. Based on the International Statistical Classification of Diseases and Related Health Problems (10th Revision), cause of deaths such as prostate-, bladder-, and lung-related deaths were considered interested ones, whereas events such as heart disease- and chronic obstructive pulmonary disease-related deaths were considered competing ones^9^. Chemotherapy was excluded due to that the information was missing in most records. After incomplete medical records, only autopsy or death certificate, and female patients were removed, 343 male lung adenocarcinoma patients with subsequent bladder papillary transitional cell carcinoma (LCBC), 1539 lung adenocarcinoma patients with prior bladder papillary transitional cell carcinoma (BCLC), 1181 lung adenocarcinoma patients with subsequent prostate adenocarcinoma (LCPC), 7426 lung adenocarcinoma patients with prior prostate adenocarcinoma (PCLC), 120,237 patients with single bladder papillary transitional cell carcinoma, 941,818 patients with single prostate adenocarcinoma, and 136,514 patients with single lung adenocarcinoma were identified (Tables 1, s1).

**Table 1:** characteristics of two primary cancer.

	LCBC				BCLC				LCPC				PCLC			
	Overall	sTPC	mTPC1	mTPC2	Overall	sTPC	mTPC1	mTPC2	Overall	sTPC	mTPC1	mTPC2	Overall	sTPC	mTPC1	mTPC2
**No**																
	343	118	143	82	1539	182	651	706	1181	374	538	269	7426	685	2969	3772
**Race**																
NHW	314	113	124	77	1363	166	570	627	867	261	400	206	5422	484	2154	2784
NHB	15	4	11	0	72	12	30	30	212	79	87	46	1252	128	528	596
NHA	6	1	2	3	59	3	26	30	52	18	26	8	372	37	145	190
Hispanic	8	0	6	2	43	1	25	17	48	16	23	9	358	36	132	190
Others	0	0	0	0	2	0	0	2	2	0	2	0	22	0	10	12
**Sex**																
	Male	Male	Male	Male	Male	Male	Male	Male	Male	Male	Male	Male	Male	Male	Male	Male
**First diagnosis of year**																
> 2005	145	64	66	15	510	108	311	91	449	159	159	131	1653	154	518	981
1995–2005	119	32	49	38	596	47	222	327	366	89	176	101	3559	221	1205	2133
< 1995	79	22	28	29	433	27	118	288	366	126	203	37	2214	310	1246	658
**Age of first diagnosis**																
Median	70	71	71	67	69	71	71	65	67	71	67	62	68	70	70	67
IQR	64–76	66–77	64–78	60–72	62–75	67–78	65–77	58–72	61–73	65–76	62–73	56–67	63–73	65–75	65–75	62–72
**First insurance status**																
No	1	1	0	0	3	1	1	1	6	1	4	1	17	2	9	6
Yes	130	55	62	13	412	92	259	61	306	109	171	26	1587	242	944	401
Unknown	212	62	81	69	1124	89	391	644	869	264	363	242	5822	441	2016	3365
**First marital status**																
Single	82	31	36	15	333	45	166	122	306	138	117	51	1568	159	675	734
Married	254	86	102	66	1147	132	459	556	849	227	408	214	5146	446	1959	2741
Unknown	7	1	5	1	59	5	26	28	26	9	13	4	712	80	335	297
**First site**																
	17	17	17	17	3	3	3	3	17	17	17	17	16	16	16	16
**First histology**																
	8140	8140	8140	8140	8130	8130	8130	8130	8140	8140	8140	8140	8140	8140	8140	8140
**First cancer stage**																
Localized	124	18	59	47	1411	148	601	662	403	56	243	104	6448	502	2632	3314
Regional	94	32	45	17	99	26	39	34	278	62	147	69				
Distant	70	47	21	2	2	1	0	1	204	138	51	15	180	69	69	42
Unstaged	55	21	18	16	27	7	11	9	296	118	97	81	798	114	268	416
**First surgery**																
No	140	74	61	5	51	7	24	20	403	254	131	18	4367	503	1947	1917
Yes	199	41	81	77	1485	175	624	686	762	108	404	250	2966	159	987	1820
Unknown	4	3	1	0	3		3	0	16	12	3	1	93	23	35	35
**First radiotherapy**																
No	2	2	0	0					3	2		1	59	4	30	25
Yes	130	56	58	16	24	6	12	6	325	138	135	52	3053	177	1300	1576
Unknown	211	60	85	66	1515	176	639	700	853	234	403	216	4314	504	1639	2171
**Interval time (month)**																
Median	21	1	25	100.5	55	2	31	117	21	1	26	104	62	2	31	108
IQR	3–58	0–4	15–40	75–162	22–108	1–4	19–46	83–156	3–56	0–3	15–40	80–141	26–109	1–4	19–45	81–147
**Secondary insurance**																
No	1	1	0	0	5	0	3	2	3	2	1		34	4	16	14
Yes	174	53	72	49	852	93	348	411	401	89	200	112	4363	279	1438	2646
Unknown	168	64	71	33	682	89	300	293	777	283	337	157	3029	402	1515	1112
**Secondary marital status**																
Single	88	32	36	20	422	44	184	194	297	128	111	58	2059	169	807	1083
Married	230	80	95	55	1061	132	447	482	754	211	363	180	5088	488	2051	2549
Unknown	25	6	12	7	56	6	20	30	130	35	64	31	279	28	111	140
**Secondary diagnosis of year**																
> 2005	199	64	80	55	944	109	385	450	343	157	138	48	669	153	340	176
1995–2005	91	33	39	19	398	46	180	172	314	90	143	81	1961	217	1005	739
< 1995	53	21	24	8	197	27	86	84	524	127	257	140	4796	315	1624	2857
**Age of secondary diagnosis**																
Median	73	71	73	76	75	72	73	76	70	71	69	72	75	70	73	77
IQR	67–80	66–77	66–80	70–82	69–80	67–78	68–80	70–82	65–76	65–76	65–75	67–77	69–80	65–75	68–78	72–82
**Secondary site**																
	3	3	3	3	17	17	17	17	16	16	16	16	17	17	17	17
**Secondary histology**																
	8130	8130	8130	8130	8140	8140	8140	8140	8140	8140	8140	8140	8140	8140	8140	8140
**Secondary cancer stage**																
Localized	283	98	120	65	288	54	112	122	824	204	389	231	1270	125	497	648
Regional	21	11	6	4	277	37	130	110	-	-	-	-	1400	165	575	660
Distant	3	2	0	1	753	66	319	368	83	39	30	14	3855	286	1584	1985
Unstaged	36	7	17	12	221	25	90	106	274	131	119	24	901	109	313	479
**Secondary surgery**																
No	23	10	10	3	1131	118	467	546	846	279	390	177	5586	432	2206	2948
Yes	320	108	133	79	389	62	177	150	304	78	137	89	1771	241	736	794
Unknown	0	0	0	0	19	2	7	10	31	17	11	3	69	12	27	30
**Secondary radiotherapy**																
No	0	0	0	0	15	1	5	9	5	2	3	0	75	2	35	38
Yes	12	7	2	3	568	55	245	268	355	59	190	106	2709	245	1155	1309
Unknown	331	111	141	79	956	126	401	429	821	313	345	163	4642	438	1779	2425
**Survival time since lung primary (month)**																
Mean	53	11	52	160.5	7	11	8	6	66	9	70	167	7	11	8	7
IQR	20–125	44–705	33–95	107–217	44–611	4–33	44–612	44–608	18–132	3–21.5	39–108	124–218	44–613	3–32	44–614	44–610
**Major cause of cancer-related death**																
	LUAC	LUAC	LUAC	LUAC	LUAC	LUAC	LUAC	LUAC	LUAC	LUAC	LUAC	LUAC	LUAC	LUAC	LUAC	LUAC
**Major cause of noncancer-related death**																
	HD	HD	HD	HD	HD	HD	HD	HD	HD	HD	HD	HD	HD	HD	HD	HD

*LCBC* lung cancer with subsequent bladder cancer; *BCLC* bladder cancer with subsequent lung cancer; *LCPC* lung cancer with subsequent prostate cancer; *PCLC* prostate cancer with subsequent lung cancer; *NHW* non-hispanic white; *NHB* non-hispanic black; *NHA* non-hispanic asian or pacific islander; *histology 8140* adenocarcinoma; *histology 8130* papillary transitional cell carcinoma; *site 3* bladder; *site 16* prostate; *site 17* lung and bronchus; *LUAC* lung adenocarcinoma; *HD* heart disease.

The sequence of primary cancers was determined by sequence number as reported^10^. Briefly, simultaneous two primaries (sTPC), metachronous two primary primaries (mTPC1), or mTPC2 were determined when the interval times between first and secondary primary cancers were within 6 months, between 7 and 60 months, or over 60 months, respectively. To reduce the unknown factors, such as medical progress with years, year of malignant diagnosis was classified into three groups: before 1995, between 1995 and 2005, and after 2005. Propensity matching score (PSM) program was executed to evaluate survival of the two primary cancer patients relative to single lung/bladder/prostate cancer. In brief, PSM with 1:1 ratio without replacement was performed between selected records of two primaries and single lung/prostate/bladder cancer. Briefly, caliper size was set at 0.1 and command psestimate was executed for covariates including race, year of diagnosis, age at diagnosis, insurance and marital status, as well as surgery and radiotherapy, to achieve optimal imitative effect. Next, command psmatch2 was executed with the selected linear or quadratic function of covariates and command pstest was executed to determine whether the absolute standardized difference between matched covariates was within 10% (s20 tab- s51 tab). Notably, for secondary cancer in records with two primaries and first cancer in sTPC group, all selected corresponding single primary were involved in the PSM program; for first cancer in mTPC1 group, the matched single cancers were required to survival at least 6 months; for first cancer in mTPC2 group, the single cancers were required to have a survival time of over 60 months.

All-cause survival of cancer patients was determined via median survival time, which was obtained via the Kaplan–Meier mode. Cox proportional hazard regression model with explanatory factors, such as clinical stage, age at diagnosis, and surgical and radiation status, was performed to identify factors associated with all-cause death. Proportional hazard assumption after Cox regression was tested and regression model was considered to fit the assumption if p value was over 0.05. For p value below 0.05, time-dependent Cox regression was subsequently performed to analyze the cofactors associated with survival. Competing risk modes (Fine and Gray’s competing risk regression modes) was applied to identify cofactors associated with cancer-specific death. Other variables such as race, sex, first and secondary marital/insurance status, and clarified diagnosis-year were included in the modes if *p* of univariate analysis of the indicated variable was less than 0.15.

The statistical analyses were performed using STATA. To reduce type I error after multiple comparisons, p value of cofactors associated with survival was adjusted by Benjamini–Hochberg program and p value below 0.05 was considered statistically significant. This study was approved by the Institutional Review Board of Tongji Hospital, Tongji Medical College, Huazhong University of Science and Technology. In consideration of the retrospective nature of this study, informed consent of patients was exempted.

## Results

### Basic information

Among the selected records, the median ages of diagnosed lung malignancy in LCBC, BCLC, LCPC, and PCLC were 70, 75, 67, and 75, respectively; median interval times between two primaries were 21, 55, 21, and 62 months. Localized, regional, distant, and unknown stage of lung primary accounted for proportions of 36.15%, 27.41%, 20.41%, and 16.03% in LCBC, respectively; on the contrary, they accounted for proportions of 18.71%, 18.00%, 48.93%, and 14.36% in BCLC. For patients with LCPC, the proportions of localized, regional, distant, and unknown stage of lung primary were 34.12%, 23.54%, 17.27%, and 25.06%, respectively; by contrast, the proportions were 17.10%, 18.85%, 51.91%, and 12.13% in patients with PCLC. The major cancer-related deaths in identified two primaries, single prostate cancer, single lung cancer, and single bladder cancer were lung cancer, prostate cancer, lung cancer, and bladder cancer, respectively; on the contrary, the major non-cancer-related death in identified two primaries and single primaries was heart disease (Tables 1, s1).

### Prognosis relative to single primary

Prognosis of lung cancer patients with another primary relative to the single lung primary has drawn great attention. Therefore, survival times of LCBC, BCLC, LCPC, and PCLC were subsequently matched with single primary malignancy via PSM program. A total of 696 patients with LCBC or single lung cancer were included in the cohort. Median overall survival times since lung primary of LCBC and single lung cancer were 97 and 17 months, respectively (Table 2). Incidence of all-cause and cancer-specific death of LCBC since lung malignancy was significantly lower than that of matched single primary (Coef. − 1.24, 95% CI − 1.49 to 0.99; SHR 0.42, 95% CI 0.33–0.53). When categorized by interval time, prognosis values of sTPC and mTPC2 groups were not statically different from that of matched single lung cancer (sTPC group: H.R 0.94, 95% CI 0.66–1.32; SHR 1.01, 95% CI 0.73–1.41; mTPC2 group: H.R 0.55, 95% CI 0.26–1.14; SHR 0.69, 95% CI 0.33–1.44). By contrast, increased overall survival time and decreased incidence of all-cause and cancer-specific death relative to the matched patients were observed in mTPC1 group (H.R 0.28, 95% CI 0.19–0.41; SHR 0.33, 95% CI 0.23–0.47.). For BCLC and matched single lung cancer, median overall survival times were 14 and 13 months, respectively (Table 2); incidence of all-cause and cancer-specific death was generally not different from each other (H.R 0.92, 95% CI 0.80–1.05; SHR 0.87, 95% CI 0.76–1.00). Meanwhile, overall survival time of LCBC and BCLC was generally shorter than that of matched single bladder cancer, and incidence of all-cause and cancer-specific death in the two primaries was higher than the matched ones either (Tables s2–s9).

**Table 2:** Overall-survival of LCBC, BCLC and matched single lung/bladder cancer.

	LCBC																BCLC															
	Overall				sTPC				mTPC1				mTPC2				Overall				sTPC				mTPC1				mTPC2			
	No	0.5	95% CI		No	0.5	95% CI		No	0.5	95% CI		No	0.5	95% CI		No	0.5	95% CI		No	0.5	95% CI		No	0.5	95% CI		No	0.5	95% CI	
SLC	249	17	13	21	97	10	7	16	118	27	20	35	58	273	142	–	583	13	11	15	139	16	12	19	358	13	11	17	293	13	10	16
TPC	262	97	67	154*	104	16	10	21	118	103	67	154*	60	–	202	–	587	14	12	16*	138	18	11	29	347	13	10	15	304	14	12	18
SBC	95	229	158	–	19	–	32	–	63	229	229	–	63	–	–	–	337	–	–	–	93	–	229	–	226	–	–	–	176	–	–	–
TPC	93	35	21	63*	19	15	4	35*	58	42	25	–*	62	150	82	–	336	86	72	99*	89	15	11	21*	226	50	45	53	176	132	119	146

Overall survival time of all LCBC and the categorized group mTPC1 were longer than matched single lung cancer, whereas overall survival time of all LCBC, the categorized group sTPC and mTPC1 were shorter than that of corresponding single bladder cancer. For BCLC, overall survival time of all and categorized groups was generally not different from that of the matched single lung primary, whereas overall survival time of all and categorized group sTPC were shorter than that of matched single bladder cancer.

*SLC* single lung cancer; *SBC* single bladder cancer; *LCBC* lung cancer with subsequent bladder cancer; *BCLC* lung cancer with previous bladder cancer; *TPC* two primary cancer;

‘-’not reached.

‘*’indicated *p* value was < 0.05.

**Figure 1:**
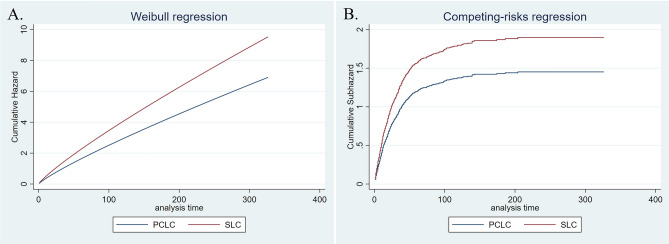
Adjusted cumulative hazard of all-cause death (**A**) and cancer-specific death (**B**) in PCLC and matched single lung cancer.

**Figure 2:**
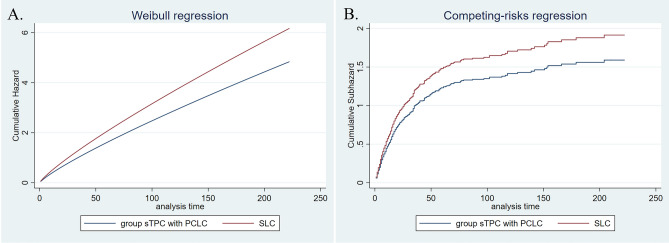
Adjusted cumulative hazard of all-cause death (**A**) and cancer-specific death (**B**) in sTPC group with PCLC and matched single lung cancer.

**Figure 3:**
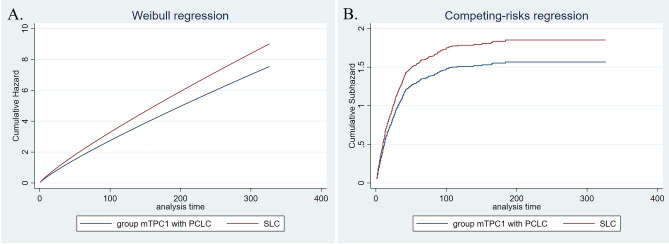
Adjusted cumulative hazard of all-cause death (**A**) and cancer-specific death (**B**) in mTPC1 group with PCLC and matched single lung cancer.

**Figure 4:**
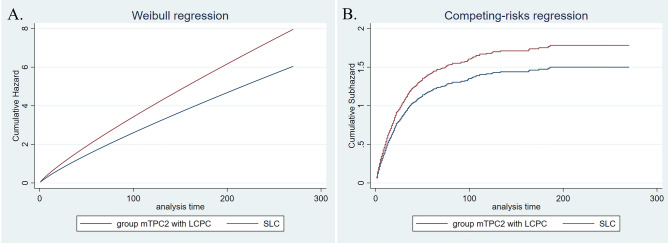
Adjusted cumulative hazard of all-cause death (**A**) and cancer-specific death (**B**) in mTPC2 group with PCLC and matched single lung cancer.

Median overall survival times of LCPC and single lung cancer involved in the cohort form PSM analysis were 116 and 16 months, respectively (Table 3); incidence of all-cause and cancer-specific death in LCPC was lower than that in corresponding lung cancer (Coef. − 1.31, 95% CI − 1.46 to 1.15; SHR 0.40, 95% CI 0.35–0.47). Further analysis suggested that prognosis of sTPC group was not statically different from that of matched single lung cancer (H.R 0.97, 95% CI 0.79–1.19; SHR 1.04, 95% CI 0.84–1.27); by contrast, survival times of mTPC1 and mTPC2 groups were improved (mTPC1 group: Coef. − 1.40, 95% CI − 1.62–1.18; SHR 0.32, 95% CI 0.26–0.40; mTPC2 group: Coef. − 0.90, 95% CI − 1.35 to 0.44; SHR 0.54, 95% CI 0.35–0.85). These results were similar to that from LCBC. Interestingly, further regression analysis suggested that PCLC generally encountered an improved survival relative to the corresponding single lung cancer (median survival times of PCLC and SLC were 17 and 12 months, respectively; PCLC vs. SLC: Coef. − 0.32, 95% CI − 0.43 to 0.22; SHR 0.77, 95% CI 0.69–0.85). However, prognosis values of LCPC and PCLC were generally inferior to that of matched single prostate cancer (Tables 3, s10–s16, Figs. 1, 2, 3 and 4).

**Table 3:** Overall-survival of LCPC, PCLC and matched single lung/prostate cancer.

	LCPC																PCLC															
	Overall				sTPC				mTPC1				mTPC2				Overall				sTPC				mTPC1				mTPC2			
	No	0.5	95% CI		No	0.5	95% CI		No	0.5	95% CI		No	0.5	95% CI		No	0.5	95%CI		No	0.5	95%CI		No	0.5	95%CI		No	0.5	95%CI	
SLC	629	16	13	19	258	11	7	14	338	30	25	35	145	273	152	–	1121	12	11	14	414	12	10	14	826	12	10	13	783	13	11	15
TPC	653	116	89	143*	256	14	11	16	338	119	99	137*	147	326	266	–*	1151	17	16	19*	430	17	14	21*	826	17	14	20*	805	17	15	19*
SPC	555	–	–	–	257	225	173	–	336	–	–	–	165	–	177	–	585	–	235	–	365	–	236	–	696	–	220	–	678	–	–	–
TPC	504	83	63	102*	207	14	12	17*	315	102	87	126*	164	–	116	–*	609	81	70	91*	406	18	16	21*	753	49	47	52*	729	130	124	138*

Overall survival time of all LCPC, categorized group mTPC1 and mPTC2 were generally longer than that of matched single lung cancer, whereas overall survival time of all LCPC, categorized group sTPC and mPTC1 were shorter than that of matched single prostate cancer. For PCLC, overall survival time of all and the categorized groups were generally longer than that of matched single lung cancer and shorter than that of matched prostate cancer, respectively.

*LCPC* lung cancer with subsequent prostate cancer; *PCLC* prostate cancer with subsequent lung cancer; *TPC* two primary cancer.

‘-’not reached.

‘*’indicated *p* value was < 0.05.

### Survival analysis among sTPC, mTPC1, and mTPC2 groups

The median overall survival times since lung malignancy in patients with LCBC and BCLC were 103 and 11 months, respectively. The median overall survival times since lung primary of sTPC, mTPC1, and mTPC2 groups were 16, 92, and 345 months, respectively, among patients with LCBC; by contrast, the times were 18, 12, and 10 months among patients with BCLC (Table s17). After cofactors such as age and stage were adjusted, incidence of all-cause and cancer-specific death in sTPC group was significantly higher than that in mTPC1 and mTPC2 groups with LCBC (mTPC1 vs. sTPC: Coef. − 1.68, 95% CI − 2.07 to 1.29; SHR 0.25, 95% CI 0.17–0.37; mTPC2 vs. sTPC: Coef. − 3.04, 95% CI − 3.67 to 2.41; SHR 0.11, 95% CI 0.06–0.21); on the contrary, sTPC group was insignificantly different from mTPC1 and mTPC2 groups with BCLC (mTPC1 vs. sTPC: Coef. 0.09, 95% CI − 0.12 to 0.31; SHR 1.10, 95% CI 0.90–1.35; mTPC2 vs. sTPC: Coef. 0.16, 95% CI − 0.05 to 0.38; SHR 1.15, 95% CI 0.95–1.41). In detail, advanced bladder cancer was not associated with all-cause and cancer-specific death since lung cancer in patients with LCBC and BCLC. Interestingly, lung-surgery (vs. no surgery) was associated with improved prognosis in LCBC and BCLC, whereas advanced lung cancer was associated with poor prognosis of BCLC but not with that of LCBC (Table s18).

Meanwhile, median survival times since lung malignancy in patients with LCPC and PCLC were 137 and 12 months, respectively. In detail, sTPC, mTPC1, and mTPC2 groups with LCPC experienced 13, 129, and 319 months of median overall survival, respectively; by contrast, the times were 16, 12, and 11 months in patients with PCLC (s17 tab). Similar to the analysis of LCBC and BCLC, prognosis of sTPC group with LCPC was much inferior to that of mTPC1 and mTPC2 groups (mTPC1 vs. sTPC: Coef. − 1.40, 95% CI − 1.61 to 1.19; SHR 0.35, 95% CI 0.27–0.45; mTPC2 vs. sTPC: Coef. − 2.50, 95% CI − 2.84 to 2.17; SHR 0.18, 95% CI 0.13–0.25). For patients with PCLC, incidence of all-cause death since lung primary in sTPC group was lower than that in mTPC1 and mTPC2 groups, whereas cancer-specific death was not different (mTPC1 vs. sTPC: Coef. 0.17, 95% CI 0.06–0.27; SHR 1.09, 95% CI 0.98–1.22; mTPC2 vs. sTPC: Coef. 0.21, 95% CI 0.10–0.32; SHR 1.08, 95% CI 0.96–1.21). Advanced prostate cancer was associated with all-cause and cancer-specific death for patients with PCLC but not for patients with LCPC. Meanwhile, advanced stages of lung cancer and no surgery (vs. surgery) were associated with worse prognosis of LCPC and PCLC (s19 tab).

## Discussion

In this study, we depicted survival of male lung cancer patients with another primary bladder/prostate cancer relative to single primary and characterized cofactors associated with the prognosis of the two primaries since lung malignancy. Compared with single lung cancer, lung cancer with subsequent bladder cancer over 6 months and lung cancer with prior prostate cancer experienced increased median overall survival time and decreased incidence of all-cause and cancer-specific death; by contrast, the remaining encountered no statically different survival. Further analysis suggested the improved survival of mTPC1 and mTPC2 groups relative to sTPC group since lung primary in lung cancer with subsequent bladder/prostate cancer, whereas survival of mTPC1 and mTPC2 groups was not statically different from that of sTPC group in lung cancer with prior bladder cancer. Meanwhile, prognosis of the two primary patients was generically poorer than that of single bladder/prostate cancer patient. To our knowledge, this study is the first population-based systematic work of survival of male lung cancer patients with another primary bladder/prostate cancer.

Lung cancer is one of most common malignancy following urinary cancer, but studies of the two primary patients are limited. Zhou et al. reported that lung cancer patients with prior bladder/prostate cancer generally experienced inferior survival to patients without prior cancer^10^. Laccetti et al. reported that lung cancer patients with prior bladder cancer had no worse survival compared with patients without prior cancer history^11–14^. These results are conflicting, and the number of patients involved in Laccetti’s report is greater (patients diagnosed between 2004 and 2008 vs. 1992 and 2009). In this study, we included entire records with primary lung and bladder/prostate cancers linked in SEER database. We also excluded the potential bias such as medical progress accompanying with years at beginning of PSM program. Our results suggested all-cause and cancer-specific survival of lung cancer patients with prior bladder is similar to that of patients without prior bladder history, which is in consistent with Laccetti’s report and the draft guidance from the Food and Drug Administration either^15^. However, the generally improved survival of lung cancer with prior prostate cancer may complicate the results when the two primary patients were involved in the lung-clinical trial. Mechanisms mediating the survival advantage remains to be uncovered, and more data are required to interpret these results.

Meanwhile, information about prognosis of lung cancer with subsequent bladder/prostate cancer is scarcer, which may be attributed to its rareness. Our analysis suggested the time dependent impact of subsequent bladder/prostate cancer over prognosis of the two primary cancer patients: the prognosis may be better when the interval between first and secondary primary is longer^5^. These results are similar to that of female lung cancer with subsequent breast cancer, which may be attributed to the aggressive nature of secondary malignancy with prior early-stage cancer and more frequent follow-up associated lead- and length-time biases^3^. Another possible reason is an advantageous lung cancer phenotype in survivors with metachronous bladder/prostate, which is reported to be a better responder to local and systemic therapy^16–18^. Moreover, the common pathways among lung, bladder, and prostate cancers are another reason^19^. Therefore, drugs such as cisplatin during bladder chemotherapy may simultaneously target the lung cancer cells. More studies are required to reveal mechanisms mediating survival advantage of lung cancer patients with subsequent bladder/prostate cancer over 6 months.

The limitation of this study included several dimensions. First, information associated with patients’ prognosis such as chemotherapy for lung/bladder/prostate cancer, smoking history, and economic status was missing in the database, which might affect the results. However, the consistency of Cox analysis among the two primary cancer patients and PSM matching program between two primary cancer and single primary supported of the soundness of our results. Second, although this study was based on a national database, the number of records qualified for this study, especially for patients with LCBC, was still limited. This limitation may contribute to the bias in the stratified survival of two primaries. For example, survival of male lung cancer patients with subsequent bladder over 60 months (only 60 qualified records) is not statically different to that of matched sing lung primary, which was greatly improved compared with that of simultaneous lung and bladder primaries. Finally, bias was inevitable due to the properties of a retrospective epidemiological study.

Overall, our analysis suggested that prognosis of lung cancer patients with subsequent bladder/prostate over 6 months was generally improved relative to that of single lung cancer, which are similar to our previous report regarding survival of female lung cancer with subsequent breast cancer. Meanwhile, male lung cancer patients with prior urinary cancer experienced varied prognosis: survival of lung cancer with prior bladder cancer was similar to that of single lung cancer, which supported involvement of these patients into clinical trials. However, prognosis of lung cancer with prior prostate cancer was generally improved, which may complicate the results when these patients were involved into clinical trials. Further study is still required to confirm our results and clarify the mechanisms mediating survival advantage of patients with the two primary cancers.

## Supplementary Information

Supplementary Information.

## Data Availability

The data that support the findings of this study are available from the Surveillance, Epidemiology, and End Results (SEER) but restrictions apply to the availability of these data, which were used under license for the current study, and so are not publicly available. Data are available from the authors upon reasonable request and with permission of SEER.
