# Supplementary material for: Prognosis of male lung cancer patients with urinary cancer: a study from a national population-based analysis
Source: Sci Rep. 2023 Jan 6;13:283. doi: 10.1038/s41598-023-27566-8 (PMC9822891; doi:10.1038/s41598-023-27566-8)
Supplement: Supplementary file 1 — Supplementary Information. [file 41598_2023_27566_MOESM1_ESM.docx]

Tab S1. summary of identified single prostate, lung and bladder cancer

|  |  | prostate cancer | lung cancer | bladder cancer |
| --- | --- | --- | --- | --- |
| NO. | |  |  |  |
|  |  | 941818 | 136514 | 120237 |
| Race | |  |  |  |
|  | NHW | 666829 | 100,512 | 102917 |
|  | NHB | 131699 | 16746 | 4621 |
|  | NHA | 43767 | 11042 | 4507 |
|  | Hispanic | 76377 | 7563 | 6297 |
|  | Others | 23146 | 651 | 1895 |
| Year of diagnosis | |  |  |  |
|  | >2005 | 449958 | 65568 | 61286 |
|  | 1995-2005 | 320959 | 40648 | 35980 |
|  | <1995 | 170901 | 30298 | 22971 |
| Age of diagnosis | |  |  |  |
|  | Median | 67 | 67 | 70 |
|  | IQR | 61-74 | 59-74 | 60-78 |
| Insurance | |  |  |  |
|  | Uninsured | 6084 | 2228 | 1038 |
|  | Insured | 350094 | 57016 | 51912 |
|  | Unknown | 585640 | 77270 | 67287 |
| Marital status | |  |  |  |
|  | Single | 202857 | 44393 | 30930 |
|  | Married | 629467 | 87060 | 81313 |
|  | Unknown | 109494 | 5061 | 7994 |
| Site | |  |  |  |
|  |  | 16 | 17 | 6 |
| Histologic | |  |  |  |
|  |  | 8140 | 8140 | 8130 |
| SEER stage | |  |  |  |
|  | Localized | 18860 | 38117 | 100457 |
|  | Regional | 707766 | 71296 | 10126 |
|  | Distant | 34051 | 3836 | 1476 |
|  | Unknown | 181141 | 23265 | 8178 |
| Radiation | |  |  |  |
|  | No | 5,365 | 27 | 0 |
|  | Yes | 308586 | 58127 | 3223 |
|  | Unknown | 627867 | 78360 | 117014 |
| Surgery | |  |  |  |
|  | No | 515050 | 102,189 | 4886 |
|  | Yes | 410581 | 31346 | 114971 |
|  | Unknown | 16187 | 2979 | 380 |
| Major cause of cancer-related death | | |  |  |
|  |  | Prostate (30.21%) | Lung (83.17%) | Bladder (21.95%) |

| Major cause of noncancer-related death | | |  |  |
| --- | --- | --- | --- | --- |
|  |  | HD (26.95%) | HD (3.99%) | HD (29.31%) |

NHW: Non-Hispanic White; NHB: Non-Hispanic Black; NHA: Non-Hispanic Asian or Pacific Islander; HD: heart disease

Tab S2. All cause and cancer-specific death between LCBC and matched single lung cancer

|  |  | Coef. | P | 95% CI | | SHR | P | 95% CI | |
| --- | --- | --- | --- | --- | --- | --- | --- | --- | --- |
| Group | | |  |  |  |  |  |  |  |
|  | SLC | 0.00 |  |  |  | 1.00 |  |  |  |
|  | LCBC | -1.24 | 0.00 | -1.49 | -0.99 | 0.42 | 0.00 | 0.33 | 0.53 |
| Age | | 0.03 | 0.00 | 0.01 | 0.04 | 1.00 | 0.84 | 0.99 | 1.02 |
| Category | | |  |  |  |  |  |  |  |
|  | >2005 | 0.00 |  |  |  | 1.00 |  |  |  |
|  | 1995-2005 | -0.41 | 0.11 | -0.83 | 0.02 | 0.67 | 0.23 | 0.41 | 1.11 |
|  | <1995 | -0.13 | 0.81 | -0.63 | 0.37 | 0.86 | 0.81 | 0.51 | 1.46 |
| Race | | |  |  |  |  |  |  |  |
|  | NHW | 0.00 |  |  |  | 1.00 |  |  |  |
|  | NHB | 0.26 | 0.45 | -0.25 | 0.77 | 1.01 | 0.98 | 0.55 | 1.85 |
|  | NHA | -0.01 | 0.98 | -0.74 | 0.72 | 1.02 | 0.98 | 0.59 | 1.73 |
|  | Hispanic | -0.15 | 0.86 | -1.14 | 0.85 | 0.81 | 0.84 | 0.29 | 2.27 |
| Insurance | | |  |  |  |  |  |  |  |
|  | No | 0.00 |  |  |  | 1.00 |  |  |  |
|  | Yes | -1.90 | 0.12 | -3.96 | 0.16 | 0.25 | 0.00 | 0.13 | 0.48 |
|  | Unknown | -1.31 | 0.35 | -3.41 | 0.78 | 0.41 | 0.06 | 0.19 | 0.93 |
| Marital | |  |  |  |  |  |  |  |  |
|  | Single | 0.00 |  |  |  | 1.00 |  |  |  |
|  | Married | -0.36 | 0.02 | -0.61 | -0.11 | 0.71 | 0.02 | 0.55 | 0.91 |
|  | Unknown | 0.04 | 0.98 | -0.72 | 0.79 | 0.74 | 0.79 | 0.30 | 1.84 |
| SEER stage | | |  |  |  |  |  |  |  |
|  | Localized | 0.00 |  |  |  | 1.00 |  |  |  |
|  | Regional | 1.40 | 0.00 | 1.07 | 1.73 | 3.01 | 0.00 | 2.18 | 4.15 |
|  | Distant | 1.10 | 0.00 | 0.73 | 1.47 | 2.62 | 0.00 | 1.81 | 3.79 |
|  | Unknown | 0.82 | 0.00 | 0.36 | 1.28 | 2.03 | 0.00 | 1.28 | 3.22 |
| Surgery | | |  |  |  |  |  |  |  |
|  | No | 0.00 |  |  |  | 1.00 |  |  |  |
|  | Yes | -1.02 | 0.00 | -1.35 | -0.68 | 0.44 | 0.00 | 0.32 | 0.61 |
|  | Unknown | 0.18 | 0.81 | -0.58 | 0.95 | 1.24 | 0.79 | 0.70 | 2.19 |
| Radiation | | | |  |  |  |  |  |  |
|  | Do | 0.00 |  |  |  | 1.00 |  |  |  |
|  | Unknown | -0.05 | 0.83 | -0.33 | 0.22 | 0.93 | 0.82 | 0.71 | 1.24 |
| _Cons | | -3.05 | 0.02 | -5.28 | -0.83 |  |  |  |  |
| /Ln_p | | -0.16 | 0.00 | -0.25 | -0.07 |  |  |  |  |

Incidence of both all-cause and cancer specific death in LCBC were lower than that in the matched single lung cancer patients. SLC: single lung cancer; LCBC: lung cancer with subsequent bladder cancer; Coef.: regression coefficient; SHR: risk of cancer-specific death; p: adjusted p value.

Tab S3. All cause and cancer-specific death between categorized LCBC and matched single lung cancer

|  |  | sTPC | | | | | | mTPC1 | | | | | | | | | | mTPC2 | | | | | | | |
| --- | --- | --- | --- | --- | --- | --- | --- | --- | --- | --- | --- | --- | --- | --- | --- | --- | --- | --- | --- | --- | --- | --- | --- | --- | --- |
|  |  | H.R | p | 95% CI | | SHR | p | 95% CI | | H.R | p | 95% CI | | SHR | p | 95% CI | | H.R | p | 95% CI | | SHR | p | 95% CI | |
| Group | |  |  |  |  |  |  |  |  |  |  |  |  |  |  |  |  |  |  |  |  |  |  |  |  |
|  | SLC | 1.00 |  |  |  | 1.00 |  |  |  | 1.00 |  |  |  | 1.00 |  |  |  | 1.00 |  |  |  | 1.00 |  |  |  |
|  | LCBC | 0.94 | 0.76 | 0.66 | 1.32 | 1.01 | 0.94 | 0.73 | 1.41 | 0.28 | 0.00 | 0.19 | 0.40 | 0.33 | 0.00 | 0.23 | 0.47 | 0.55 | 0.51 | 0.26 | 1.14 | 0.69 | 0.64 | 0.33 | 1.44 |
| Age | | 1.02 | 0.18 | 1.00 | 1.04 | 1.00 | 0.88 | 0.98 | 1.02 | 0.99 | 0.58 | 0.97 | 1.01 | 0.98 | 0.09 | 0.96 | 1.00 | 1.08 | 0.00 | 1.03 | 1.14 | 1.04 | 0.28 | 1.00 | 1.09 |
| Category | | |  |  |  |  |  |  |  |  |  |  |  |  |  |  |  |  |  |  |  |  |  |  |  |
|  | >2005 | 1.00 |  |  |  | 1.00 |  |  |  | 1.00 |  |  |  | 1.00 |  |  |  | 1.00 |  |  |  | 1.00 |  |  |  |
|  | 1995-2005 | 0.42 | 0.03 | 0.21 | 0.81 | 0.53 | 0.21 | 0.26 | 1.09 | 2.37 | 0.41 | 0.56 | 10.00 | 3.19 | 0.30 | 0.66 | 15.54 | 0.49 | 0.92 | 0.10 | 2.42 | 0.30 | 0.62 | 0.05 | 1.73 |
|  | <1995 | 0.66 | 0.38 | 0.32 | 1.35 | 0.93 | 0.90 | 0.46 | 1.88 | 2.02 | 0.56 | 0.43 | 9.46 | 2.00 | 0.47 | 0.37 | 10.83 | 0.72 | 0.92 | 0.13 | 3.95 | 0.47 | 0.70 | 0.08 | 2.77 |
| Race | | |  |  |  |  |  |  |  |  |  |  |  |  |  |  |  |  |  |  |  |  |  |  |  |
|  | NHW | 1.00 |  |  |  | 1.00 |  |  |  |  |  |  |  |  |  |  |  | 1.00 |  |  |  | 1.00 |  |  |  |
|  | NHB | 0.76 | 0.74 | 0.27 | 2.16 | 0.78 | 0.82 | 0.27 | 2.24 |  |  |  |  |  |  |  |  | 1.28 | 0.92 | 0.36 | 4.54 | 1.57 | 0.70 | 0.43 | 5.76 |
|  | NHA | 0.60 | 0.67 | 0.13 | 2.70 | 0.85 | 0.82 | 0.46 | 1.58 |  |  |  |  |  |  |  |  | 1.14 | 0.98 | 0.14 | 9.14 | 1.08 | 0.95 | 0.12 | 9.77 |
| Insurance | | |  |  |  |  |  |  |  |  |  |  |  |  |  |  |  |  |  |  |  |  |  |  |  |
|  | No | 1.00 |  |  |  | 1.00 |  |  |  |  |  |  |  |  |  |  |  |  |  |  |  |  |  |  |  |
|  | Yes | 0.27 | 0.38 | 0.03 | 2.63 | 0.44 | 0.30 | 0.14 | 1.35 | 1.00 |  |  |  | 1.00 |  |  |  | 1.00 |  |  |  | 1.00 |  |  |  |
|  | Unknown | 0.70 | 0.76 | 0.07 | 7.13 | 0.77 | 0.82 | 0.23 | 2.55 | 0.60 | 0.58 | 0.14 | 2.52 | 0.53 | 0.47 | 0.11 | 2.58 | 1.63 | 0.92 | 0.25 | 10.89 | 1.61 | 0.84 | 0.19 | 13.72 |
| Marital | | |  |  |  |  |  |  |  |  |  |  |  |  |  |  |  |  |  |  |  |  |  |  |  |
|  | Single | 1.00 |  |  |  | 1.00 |  |  |  | 1.00 |  |  |  | 1.00 |  |  |  | 1.00 |  |  |  | 1.00 |  |  |  |
|  | Married | 0.55 | 0.00 | 0.37 | 0.80 | 0.60 | 0.04 | 0.41 | 0.88 | 1.09 | 0.77 | 0.70 | 1.68 | 1.20 | 0.47 | 0.77 | 1.86 | 0.72 | 0.92 | 0.34 | 1.55 | 0.62 | 0.62 | 0.28 | 1.35 |
|  | Unknown |  |  |  |  |  |  |  |  | 2.22 | 0.38 | 0.73 | 6.75 | 2.22 | 0.30 | 0.75 | 6.61 | 0.00 | 1.00 | 0.00 | . | 0.00 | 0.00 | 0.00 | 0.00 |
| Seerstage | | |  |  |  |  |  |  |  |  |  |  |  |  |  |  |  |  |  |  |  |  |  |  |  |
|  | Localized | 1.00 |  |  |  | 1.00 |  |  |  | 1.00 |  |  |  | 1.00 |  |  |  | 1.00 |  |  |  | 1.00 |  |  |  |
|  | Regional | 3.29 | 0.00 | 1.71 | 6.33 | 2.48 | 0.00 | 1.35 | 4.57 | 1.65 | 0.08 | 1.08 | 2.52 | 1.58 | 0.09 | 1.06 | 2.36 | 1.07 | 0.98 | 0.38 | 3.00 | 1.04 | 0.95 | 0.39 | 2.75 |
|  | Distant | 2.85 | 0.00 | 1.48 | 5.50 | 2.59 | 0.00 | 1.40 | 4.77 | 1.71 | 0.24 | 0.95 | 3.09 | 1.55 | 0.34 | 0.79 | 3.03 | 3.50 | 0.92 | 0.37 | 33.05 | 3.50 | 0.64 | 0.31 | 39.09 |
|  | Unstaged | 2.35 | 0.13 | 1.01 | 5.45 | 1.74 | 0.34 | 0.76 | 3.96 | 1.63 | 0.41 | 0.73 | 3.63 | 1.67 | 0.38 | 0.70 | 4.02 | 0.82 | 0.92 | 0.29 | 2.38 | 1.20 | 0.84 | 0.44 | 3.25 |
| Surgery | | |  |  |  |  |  |  |  |  |  |  |  |  |  |  |  |  |  |  |  |  |  |  |  |
|  | No | 1.00 |  |  |  | 1.00 |  |  |  | 1.00 |  |  |  | 1.00 |  |  |  | 1.00 |  |  |  | 1.00 |  |  |  |
|  | Yes | 0.40 | 0.00 | 0.24 | 0.65 | 0.49 | 0.00 | 0.30 | 0.79 | 0.47 | 0.00 | 0.32 | 0.70 | 0.53 | 0.00 | 0.36 | 0.80 | 1.83 | 0.92 | 0.28 | 11.79 | 1.91 | 0.70 | 0.31 | 11.71 |
|  | Unknown | 1.16 | 0.76 | 0.46 | 2.89 | 1.39 | 0.53 | 0.72 | 2.70 | 0.89 | 0.88 | 0.20 | 3.95 | 1.02 | 0.96 | 0.49 | 2.10 |  |  |  |  |  |  |  |  |
| Radiation | | |  |  |  |  |  |  |  |  |  |  |  |  |  |  |  |  |  |  |  |  |  |  |  |
|  | No | 1.00 |  |  |  | 1.00 |  |  |  |  |  |  |  |  |  |  |  |  |  |  |  |  |  |  |  |
|  | Yes | 0.42 | 0.25 | 0.13 | 1.35 | 0.36 | 0.05 | 0.15 | 0.84 |  |  |  |  |  |  |  |  | 1.00 |  |  |  | 1.00 |  |  |  |
|  | Unknown | 0.36 | 0.18 | 0.11 | 1.19 | 0.33 | 0.05 | 0.13 | 0.84 |  |  |  |  |  |  |  |  | 0.42 | 0.51 | 0.16 | 1.09 | 0.45 | 0.28 | 0.20 | 1.02 |
| phtest | |  | 0.93 |  |  |  |  |  |  |  | 0.07 |  |  |  |  |  |  |  | 0.25 |  |  |  |  |  |  |

Incidence of both all-cause and cancer specific death in categorized group mTPC1 with LCBC were lower than that in the matched single lung cancer patients. SLC: single lung cancer; LCBC: lung cancer with subsequent bladder cancer; H.R: risk of all-cause death; SHR: risk of cancer-specific death. p: adjusted p value.

Tab S4. All cause and cancer-specific death between LCBC and matched single bladder cancer

|  |  | H.R | p | 95% CI | | SHR | p | 95% CI | |
| --- | --- | --- | --- | --- | --- | --- | --- | --- | --- |
| Group | |  |  |  |  |  |  |  |  |
|  | SBC | 1.00 |  |  |  | 1.00 |  |  |  |
|  | LCBC | 4.00 | 0.00 | 2.14 | 7.47 | 3.85 | 0.00 | 1.91 | 7.77 |
| Age | | 1.00 | 0.81 | 0.97 | 1.02 | 0.98 | 0.38 | 0.96 | 1.00 |
| Category | | |  |  |  |  |  |  |  |
|  | >2005 | 1.00 |  |  |  | 1.00 |  |  |  |
|  | 1995-2005 | 1.67 | 0.81 | 0.36 | 7.83 | 1.52 | 0.65 | 0.30 | 7.83 |
|  | <1995 | 1.48 | 0.81 | 0.31 | 7.18 | 1.85 | 0.63 | 0.35 | 9.73 |
| Race | | |  |  |  |  |  |  |  |
|  | NHW | 1.00 |  |  |  | 1.00 |  |  |  |
|  | NHB | 1.17 | 0.81 | 0.58 | 2.36 | 1.29 | 0.63 | 0.70 | 2.36 |
|  | NHA | 1.14 | 0.84 | 0.43 | 3.05 | 1.55 | 0.46 | 0.77 | 3.11 |
|  | Hispanic | 0.19 | 0.59 | 0.03 | 1.44 | 0.20 | 0.38 | 0.03 | 1.39 |
| Insurance | | |  |  |  |  |  |  |  |
|  | No | 1.00 |  |  |  | 1.00 |  |  |  |
|  | Yes | 0.38 | 0.78 | 0.04 | 3.70 | 0.42 | 0.40 | 0.13 | 1.35 |
|  | Unknown | 0.24 | 0.78 | 0.02 | 3.59 | 0.24 | 0.38 | 0.04 | 1.45 |
| Marital | | |  |  |  |  |  |  |  |
|  | Single | 1.00 |  |  |  | 1.00 |  |  |  |
|  | Married | 0.45 | 0.00 | 0.26 | 0.76 | 0.41 | 0.00 | 0.24 | 0.70 |
|  | Unknown | 0.60 | 0.78 | 0.17 | 2.16 | 0.70 | 0.65 | 0.19 | 2.60 |
| SEER stage | | |  |  |  |  |  |  |  |
|  | Localized | 1.00 |  |  |  | 1.00 |  |  |  |
|  | Regional | 1.64 | 0.72 | 0.80 | 3.38 | 1.33 | 0.63 | 0.61 | 2.89 |
|  | Distant | 2.13 | 0.78 | 0.41 | 11.13 | 2.11 | 0.62 | 0.44 | 10.15 |
|  | Unknown | 1.11 | 0.85 | 0.37 | 3.34 | 0.91 | 0.86 | 0.31 | 2.67 |
| Surgery | | |  |  |  |  |  |  |  |
|  | No | 1.00 |  |  |  | 1.00 |  |  |  |
|  | Yes | 0.82 | 0.81 | 0.41 | 1.64 | 0.81 | 0.65 | 0.40 | 1.63 |
| Radiation | | |  |  |  |  |  |  |  |
|  | Yes | 1.00 |  |  |  | 1.00 |  |  |  |
|  | Unknown | 0.63 | 0.78 | 0.28 | 1.43 | 0.57 | 0.46 | 0.22 | 1.42 |
| phtest | | | 0.50 |  |  |  |  |  |  |

Incidence of both all-cause and cancer specific death in LCBC were higher than that in the matched single bladder cancer. SBC: single bladder cancer; LCBC: lung cancer with subsequent bladder cancer; H.R: risk of all-cause death; SHR: risk of cancer-specific death. p: adjusted p value.

Tab S5. All cause and cancer-specific death between categorized LCBC and matched single bladder cancer

|  |  |  | | | | | | | |  | | | | | | | |  | | | | | | | |
| --- | --- | --- | --- | --- | --- | --- | --- | --- | --- | --- | --- | --- | --- | --- | --- | --- | --- | --- | --- | --- | --- | --- | --- | --- | --- |
|  |  | H.R | p | 95% CI | | SHR | p | 95% CI | | H.R | p | 95% CI | | SHR | p | 95% CI | | H.R | p | 95% CI | | SHR | p | 95% CI | |
| Group | | |  |  |  |  |  |  |  |  |  |  |  |  |  |  |  |  |  |  |  |  |  |  |  |
|  | SBC | 1.00 |  |  |  | 1.00 |  |  |  | 1.00 |  |  |  | 1.00 |  |  |  | 1.00 |  |  |  |  |  |  |  |
|  | LCBC | 29.46 | 0.00 | 5.20 | 166.75 | 29.99 | 0.00 | 6.90 | 130.37 | 9.59 | 0.00 | 3.82 | 24.08 | 7.62 | 0.00 | 2.89 | 20.07 | 2.13 | 0.26 | 0.75 | 6.03 | 2.10 | 0.07 | 1.07 | 6.79 |
| Age | | 1.00 | 0.95 | 0.94 | 1.06 | 0.99 | 0.87 | 0.93 | 1.06 | 0.97 | 0.35 | 0.93 | 1.01 | 0.96 | 0.28 | 0.92 | 1.00 | 1.00 | 0.93 | 0.92 | 1.08 | -1.01 | 0.47 | 0.89 | 1.04 |
| Category | | | |  |  |  |  |  |  |  |  |  |  |  |  |  |  |  |  |  |  |  |  |  |  |
|  | >2005 |  |  |  |  |  |  |  |  | 1.00 |  |  |  | 1.00 |  |  |  | 1.00 |  |  |  |  |  |  |  |
|  | 1995-2005 |  |  |  |  |  |  |  |  | 1.47 | 0.85 | 0.18 | 11.92 | 1.99 | 0.57 | 0.29 | 13.47 | 0.23 | 0.09 | 0.07 | 0.84 | -2.72 | 0.02 | 0.06 | 0.63 |
|  | <1995 |  |  |  |  |  |  |  |  | 1.73 | 0.75 | 0.26 | 11.71 | 2.27 | 0.54 | 0.43 | 11.98 | 0.28 | 0.18 | 0.07 | 1.22 | -2.58 | 0.02 | 0.10 | 0.73 |
| Race | | |  |  |  |  |  |  |  |  |  |  |  |  |  |  |  |  |  |  |  |  |  |  |  |
|  | NHW | 1.00 |  |  |  | 1.00 |  |  |  | 1.00 |  |  |  | 1.00 |  |  |  | 1.00 |  |  |  |  |  |  |  |
|  | NHB | 0.58 | 0.65 | 0.09 | 3.53 | 0.48 | 0.33 | 0.14 | 1.67 | 0.97 | 0.94 | 0.42 | 2.23 | 1.32 | 0.57 | 0.64 | 2.72 | 6.69 | 0.09 | 1.17 | 38.22 | 2.92 | 0.00 | 1.97 | 31.73 |
|  | NHA | 0.37 | 0.57 | 0.03 | 4.44 | 0.35 | 0.26 | 0.10 | 1.30 | 0.13 | 0.21 | 0.02 | 1.05 | 0.22 | 0.36 | 0.03 | 1.42 | 1.96 | 0.72 | 0.22 | 17.55 | 0.86 | 0.47 | 0.34 | 15.37 |
| Insurance | | |  |  |  |  |  |  |  |  |  |  |  |  |  |  |  |  |  |  |  |  |  |  |  |
|  | No | 1.00 |  |  |  | 1.00 |  |  |  | 1.00 |  |  |  | 1.00 |  |  |  |  |  |  |  |  |  |  |  |
|  | Yes | 0.10 | 0.41 | 0.00 | 3.58 | 0.10 | 0.23 | 0.01 | 1.20 | 0.75 | 0.86 | 0.09 | 6.10 | 0.45 | 0.56 | 0.07 | 2.75 | 1.00 |  |  |  |  |  |  |  |
|  | Unknown | 0.91 | 0.95 | 0.04 | 22.42 | 0.87 | 0.89 | 0.11 | 6.95 |  |  |  |  |  |  |  |  | 12.42 | 0.09 | 1.49 | 103.26 | 2.69 | 0.02 | 2.11 | 115.94 |
| Marital | | | |  |  |  |  |  |  |  |  |  |  |  |  |  |  |  |  |  |  |  |  |  |  |
|  | Single | 1.00 |  |  |  | 1.00 |  |  |  | 1.00 |  |  |  | 1.00 |  |  |  | 1.00 |  |  |  |  |  |  |  |
|  | Married | 0.46 | 0.41 | 0.14 | 1.59 | 0.48 | 0.30 | 0.15 | 1.49 | 0.15 | 0.00 | 0.06 | 0.39 | 0.18 | 0.00 | 0.07 | 0.45 | 0.28 | 0.12 | 0.08 | 1.01 | -2.04 | 0.07 | 0.09 | 0.95 |
|  | Unknown | 0.04 | 0.04 | 0.00 | 0.46 | 0.05 | 0.00 | 0.01 | 0.38 | 0.25 | 0.21 | 0.06 | 1.10 | 0.35 | 0.40 | 0.08 | 1.51 | 0.63 | 0.72 | 0.11 | 3.51 | -0.53 | 0.61 | 0.07 | 4.44 |
| SEER stage | | |  |  |  |  |  |  |  |  |  |  |  |  |  |  |  |  |  |  |  |  |  |  |  |
|  | Localized | 1.00 |  |  |  | 1.00 |  |  |  | 1.00 |  |  |  | 1.00 |  |  |  | 1.00 |  |  |  |  |  |  |  |
|  | Regional | 3.56 | 0.41 | 0.53 | 23.88 | 3.45 | 0.28 | 0.64 | 18.62 | 0.70 | 0.75 | 0.19 | 2.49 | 1.08 | 0.91 | 0.27 | 4.32 | 1.53 | 0.77 | 0.16 | 14.79 | 0.52 | 0.61 | 0.25 | 10.93 |
|  | Distant | 3.40 | 0.41 | 0.53 | 22.01 | 3.54 | 0.30 | 0.48 | 25.85 |  |  |  |  |  |  |  |  |  |  |  |  |  |  |  |  |
|  | Unknown | 3.02 | 0.57 | 0.26 | 34.74 | 3.44 | 0.26 | 0.73 | 16.35 | 3.37 | 0.26 | 0.79 | 14.34 | 1.51 | 0.62 | 0.37 | 6.21 | 41.68 | 0.09 | 2.32 | 749.95 | 2.77 | 0.02 | 3.37 | 1224.27 |
| Surgery | | |  |  |  |  |  |  |  |  |  |  |  |  |  |  |  |  |  |  |  |  |  |  |  |
|  | No | 1.00 |  |  |  | 1.00 |  |  |  | 1.00 |  |  |  | 1.00 |  |  |  |  |  |  |  |  |  |  |  |
|  | Yes | 0.14 | 0.04 | 0.03 | 0.63 | 0.16 | 0.09 | 0.04 | 0.71 | 0.67 | 0.75 | 0.19 | 2.42 | 0.47 | 0.40 | 0.14 | 1.56 |  |  |  |  |  |  |  |  |
| Radiation | | |  |  |  |  |  |  |  |  |  |  |  |  |  |  |  |  |  |  |  |  |  |  |  |
|  | Yes | 1.00 |  |  |  | 1.00 |  |  |  | 1.00 |  |  |  | 1.00 |  |  |  | 1.00 |  |  |  |  |  |  |  |
|  | Unknown | 0.50 | 0.57 | 0.10 | 2.48 | 0.56 | 0.46 | 0.14 | 2.15 | 0.23 | 0.35 | 0.03 | 2.08 | 0.27 | 0.40 | 0.04 | 1.85 | 0.28 | 0.51 | 0.02 | 3.85 | -0.92 | 0.47 | 0.02 | 4.13 |
| phtest | | | 0.9964 |  |  |  |  |  |  |  | 0.09 |  |  |  |  |  |  |  | 0.99 |  |  |  |  |  |  |

Incidence of both all-cause and cancer specific death in categorized group sTPC and mTPC1 with LCBC were higher than that in the matched single bladder cancer. SBC: single bladder cancer; LCBC: lung cancer with subsequent bladder cancer; H.R: risk of all-cause death; SHR: risk of cancer-specific death; p: adjusted p value.

Tab S6. All cause and cancer-specific death between BCLC and matched single lung cancer

|  |  | H.R | p | 95% CI | | SHR | p | 95% CI | |
| --- | --- | --- | --- | --- | --- | --- | --- | --- | --- |
| Group | |  |  |  |  |  |  |  |  |
|  | SLC | 1.00 |  |  |  | 1.00 |  |  |  |
|  | BCLC | 0.92 | 0.47 | 0.80 | 1.05 | 0.87 | 0.18 | 0.76 | 1.00 |
| Age | | 1.01 | 0.07 | 1.00 | 1.02 | 1.01 | 0.28 | 1.00 | 1.01 |
| Category | |  |  |  |  |  |  |  |  |
|  | >2005 | 1.00 |  |  |  | 1.00 |  |  |  |
|  | 1995-2005 | 1.04 | 0.88 | 0.78 | 1.37 | 0.99 | 0.95 | 0.73 | 1.34 |
|  | <1995 | 1.08 | 0.78 | 0.80 | 1.46 | 1.02 | 0.95 | 0.74 | 1.42 |
| Race | |  |  |  |  |  |  |  |  |
|  | NHW | 1.00 |  |  |  | 1.00 |  |  |  |
|  | NHB | 1.01 | 0.93 | 0.79 | 1.30 | 1.02 | 0.95 | 0.79 | 1.31 |
|  | NHA | 0.72 | 0.09 | 0.53 | 0.97 | 0.76 | 0.21 | 0.56 | 1.02 |
|  | Hispanic | 1.24 | 0.44 | 0.91 | 1.68 | 1.22 | 0.40 | 0.90 | 1.64 |
|  | Others | 1.63 | 0.71 | 0.40 | 6.66 | 1.97 | 0.40 | 0.72 | 5.45 |
| Insurance | |  |  |  |  |  |  |  |  |
|  | No | 1.00 |  |  |  | 1.00 |  |  |  |
|  | Yes | 0.84 | 0.88 | 0.21 | 3.42 | 1.74 | 0.65 | 0.44 | 6.87 |
|  | Unknown | 1.16 | 0.88 | 0.28 | 4.78 | 2.34 | 0.41 | 0.58 | 9.45 |
| Marital | |  |  |  |  |  |  |  |  |
|  | Single | 1.00 |  |  |  | 1.00 |  |  |  |
|  | Married | 1.06 | 0.71 | 0.91 | 1.23 | 1.04 | 0.87 | 0.89 | 1.21 |
|  | Unknown | 0.82 | 0.48 | 0.58 | 1.14 | 0.85 | 0.57 | 0.60 | 1.20 |
| SEER stage | |  |  |  |  |  |  |  |  |
|  | Localized | 1.00 |  |  |  | 1.00 |  |  |  |
|  | Regional | 2.00 | 0.00 | 1.65 | 2.43 | 1.99 | 0.00 | 1.65 | 2.39 |
|  | Distant | 1.92 | 0.00 | 1.56 | 2.37 | 2.11 | 0.00 | 1.72 | 2.58 |
|  | Unknown | 1.47 | 0.05 | 1.12 | 1.93 | 1.56 | 0.00 | 1.19 | 2.05 |
| Surgery | |  |  |  |  |  |  |  |  |
|  | No | 1.00 |  |  |  | 1.00 |  |  |  |
|  | Yes | 0.36 | 0.00 | 0.29 | 0.43 | 0.47 | 0.00 | 0.39 | 0.56 |
|  | Unknown | 1.19 | 0.71 | 0.72 | 1.95 | 0.90 | 0.89 | 0.53 | 1.52 |
| Radiation | |  |  |  |  |  |  |  |  |
|  | Yes | 1.00 |  |  |  | 1.00 |  |  |  |
|  | Unknown | 1.05 | 0.71 | 0.90 | 1.22 | 1.02 | 0.91 | 0.88 | 1.20 |
| phtest | |  | 0.17 |  |  |  |  |  |  |

Incidence of all-cause and cancer specific death in BCLC were not statically different from that in the matched single lung cancer. SLC: single lung cancer; BCLC: bladder cancer with subsequent lung cancer; H.R: risk of all-cause death; SHR: risk of cancer-specific death; p: adjusted p value.

Tab S7. All cause and cancer-specific death between categorized group with BCLC and matched single lung cancer

|  |  | sTPC | | | | | | | | mTPC1 | | | | | | | | mTPC2 | | | | | | | |
| --- | --- | --- | --- | --- | --- | --- | --- | --- | --- | --- | --- | --- | --- | --- | --- | --- | --- | --- | --- | --- | --- | --- | --- | --- | --- |
|  |  | H.R | p | 95% CI | | SHR | p | 95% CI | | H.R | p | 95% CI | | SHR | p | 95% CI | | H.R | p | 95% CI | | SHR | p | 95% CI | |
| Group | | |  |  |  |  |  |  |  |  |  |  |  |  |  |  |  |  |  |  |  |  |  |  |  |
|  | SLC | 1.00 |  |  |  | 1.00 |  |  |  | 1.00 |  |  |  | 1.00 |  |  |  | 1.00 |  |  |  | 1.00 |  |  |  |
|  | BCLC | 1.04 | 0.83 | 0.78 | 1.40 | 0.98 | 0.89 | 0.73 | 1.30 | 1.14 | 0.28 | 0.95 | 1.36 | 1.13 | 0.33 | 0.94 | 1.35 | 0.93 | 0.58 | 0.76 | 1.13 | 0.92 | 0.56 | 0.76 | 1.12 |
| Age | | 1.02 | 0.04 | 1.01 | 1.04 | 1.03 | 0.03 | 1.01 | 1.05 | 1.01 | 0.32 | 1.00 | 1.02 | 1.00 | 0.99 | 0.99 | 1.01 | 1.00 | 0.54 | 0.99 | 1.02 | 1.00 | 0.71 | 0.99 | 1.01 |
| Category | |  |  |  |  |  |  |  |  |  |  |  |  |  |  |  |  |  |  |  |  |  |  |  |  |
|  | >2005 | 1.00 |  |  |  | 1.00 |  |  |  | 1.00 |  |  |  | 1.00 |  |  |  | 1.00 |  |  |  | 1.00 |  |  |  |
|  | 1995-2005 | 1.41 | 0.56 | 0.78 | 2.56 | 1.74 | 0.23 | 0.85 | 3.54 | 1.45 | 0.00 | 1.18 | 1.79 | 1.39 | 0.00 | 1.14 | 1.71 | 0.92 | 0.68 | 0.60 | 1.39 | 0.91 | 0.74 | 0.59 | 1.40 |
|  | <1995 | 1.46 | 0.56 | 0.73 | 2.93 | 2.07 | 0.14 | 0.97 | 4.39 | 1.45 | 0.03 | 1.11 | 1.90 | 1.32 | 0.09 | 1.00 | 1.73 | 0.91 | 0.68 | 0.59 | 1.41 | 0.91 | 0.74 | 0.57 | 1.45 |
| Race | |  |  |  |  |  |  |  |  |  |  |  |  |  |  |  |  |  |  |  |  |  |  |  |  |
|  | NHW | 1.00 |  |  |  | 1.00 |  |  |  | 1.00 |  |  |  | 1.00 |  |  |  | 1.00 |  |  |  | 1.00 |  |  |  |
|  | NHB | 1.26 | 0.70 | 0.67 | 2.38 | 1.67 | 0.14 | 0.98 | 2.82 | 0.67 | 0.09 | 0.46 | 0.97 | 0.66 | 0.08 | 0.46 | 0.95 | 1.58 | 0.08 | 1.08 | 2.32 | 1.71 | 0.00 | 1.24 | 2.36 |
|  | NHA | 1.31 | 0.70 | 0.46 | 3.70 | 1.07 | 0.89 | 0.40 | 2.92 | 0.82 | 0.46 | 0.54 | 1.26 | 1.01 | 1.00 | 0.66 | 1.54 | 0.68 | 0.22 | 0.44 | 1.04 | 0.65 | 0.12 | 0.42 | 0.99 |
|  | Hispanic | 2.07 | 0.57 | 0.49 | 8.75 | 2.12 | 0.00 | 1.49 | 3.02 | 1.21 | 0.46 | 0.81 | 1.80 | 1.16 | 0.59 | 0.80 | 1.68 | 1.77 | 0.05 | 1.18 | 2.64 | 1.56 | 0.08 | 1.04 | 2.32 |
|  | Others |  |  |  |  |  |  |  |  |  |  |  |  |  |  |  |  | 0.35 | 0.47 | 0.05 | 2.52 | 0.27 | 0.43 | 0.03 | 2.47 |
| Insurance | | |  |  |  |  |  |  |  |  |  |  |  |  |  |  |  |  |  |  |  |  |  |  |  |
|  | No |  |  |  |  |  |  |  |  |  |  |  |  |  |  |  |  | 1.00 |  |  |  | 1.00 |  |  |  |
|  | Yes | 1.00 |  |  |  | 1.00 |  |  |  |  |  |  |  |  |  |  |  | 2.27 | 0.47 | 0.55 | 9.34 | 1.93 | 0.56 | 0.43 | 8.64 |
|  | Unknown | 1.20 | 0.70 | 0.69 | 2.09 | 1.05 | 0.89 | 0.52 | 2.11 |  |  |  |  |  |  |  |  | 4.10 | 0.19 | 0.95 | 17.68 | 3.27 | 0.25 | 0.69 | 15.40 |
| Marital | |  |  |  |  |  |  |  |  |  |  |  |  |  |  |  |  |  |  |  |  |  |  |  |  |
|  | Single | 1.00 |  |  |  | 1.00 |  |  |  | 1.00 |  |  |  | 1.00 |  |  |  | 1.00 |  |  |  | 1.00 |  |  |  |
|  | Married | 1.29 | 0.45 | 0.90 | 1.84 | 1.22 | 0.45 | 0.85 | 1.74 | 0.99 | 0.95 | 0.82 | 1.20 | 1.00 | 1.00 | 0.83 | 1.21 | 1.05 | 0.68 | 0.85 | 1.31 | 0.97 | 0.79 | 0.78 | 1.20 |
|  | Unknown | 1.37 | 0.70 | 0.41 | 4.53 | 1.57 | 0.54 | 0.58 | 4.23 | 0.92 | 0.85 | 0.52 | 1.65 | 0.81 | 0.61 | 0.45 | 1.47 | 0.70 | 0.30 | 0.44 | 1.12 | 0.85 | 0.63 | 0.52 | 1.37 |
| SEER stage | | |  |  |  |  |  |  |  |  |  |  |  |  |  |  |  |  |  |  |  |  |  |  |  |
|  | Localized | 1.00 |  |  |  | 1.00 |  |  |  | 1.00 |  |  |  | 1.00 |  |  |  | 1.00 |  |  |  | 1.00 |  |  |  |
|  | Regional | 2.07 | 0.00 | 1.34 | 3.18 | 2.23 | 0.00 | 1.51 | 3.30 | 2.33 | 0.00 | 1.79 | 3.05 | 2.18 | 0.00 | 1.70 | 2.81 | 1.76 | 0.00 | 1.31 | 2.35 | 1.76 | 0.00 | 1.34 | 2.31 |
|  | Distant | 1.80 | 0.04 | 1.17 | 2.78 | 2.02 | 0.00 | 1.30 | 3.13 | 1.92 | 0.00 | 1.46 | 2.53 | 1.88 | 0.00 | 1.46 | 2.43 | 1.66 | 0.00 | 1.22 | 2.25 | 1.87 | 0.00 | 1.40 | 2.50 |
|  | Unknown | 1.69 | 0.45 | 0.81 | 3.55 | 1.25 | 0.72 | 0.61 | 2.58 | 1.83 | 0.00 | 1.27 | 2.62 | 1.83 | 0.00 | 1.28 | 2.60 | 1.39 | 0.24 | 0.94 | 2.05 | 1.41 | 0.17 | 0.97 | 2.06 |
| Surgery | | |  |  |  |  |  |  |  |  |  |  |  |  |  |  |  |  |  |  |  |  |  |  |  |
|  | No | 1.00 |  |  |  | 1.00 |  |  |  | 1.00 |  |  |  | 1.00 |  |  |  | 1.00 |  |  |  | 1.00 |  |  |  |
|  | Yes | 0.29 | 0.00 | 0.19 | 0.45 | 0.39 | 0.00 | 0.26 | 0.58 | 0.30 | 0.00 | 0.23 | 0.39 | 0.38 | 0.00 | 0.30 | 0.48 | 0.35 | 0.00 | 0.27 | 0.47 | 0.47 | 0.00 | 0.37 | 0.61 |
|  | Unknown | 1.63 | 0.70 | 0.38 | 7.06 | 1.83 | 0.14 | 0.95 | 3.54 | 0.84 | 0.80 | 0.37 | 1.93 | 0.63 | 0.50 | 0.25 | 1.59 | 0.77 | 0.67 | 0.33 | 1.82 | 0.63 | 0.43 | 0.28 | 1.44 |
| Radiation | | |  |  |  |  |  |  |  |  |  |  |  |  |  |  |  |  |  |  |  |  |  |  |  |
|  | No |  |  |  |  |  |  |  |  |  |  |  |  |  |  |  |  | 1.00 |  |  |  | 1.00 |  |  |  |
|  | Yes | 1.00 |  |  |  | 1.00 |  |  |  | 1.00 |  |  |  | 1.00 |  |  |  | 0.49 | 0.47 | 0.12 | 2.01 | 0.48 | 0.00 | 0.32 | 0.73 |
|  | Unknown | 1.01 | 0.97 | 0.73 | 1.40 | 1.05 | 0.89 | 0.74 | 1.47 | 1.17 | 0.21 | 0.97 | 1.43 | 1.23 | 0.09 | 1.01 | 1.50 | 0.48 | 0.47 | 0.12 | 1.97 | 0.43 | 0.00 | 0.28 | 0.66 |
| phtest | |  | 0.31 |  |  |  |  |  |  |  | 0.11 |  |  |  |  |  |  |  | 0.48 |  |  |  |  |  |  |

Incidence of all-cause and cancer specific death in categorized group with BCLC were not statically different from that in the matched single lung cancer. SLC: single lung cancer; BCLC: bladder cancer with subsequent lung cancer; H.R: risk of all-cause death; SHR: risk of cancer-specific death; p: adjusted p value.

Tab S8. All cause and cancer-specific death between BCLC and matched single bladder cancer

|  |  | Coef. | p | 95% CI | | SHR | p | 95% CI | |
| --- | --- | --- | --- | --- | --- | --- | --- | --- | --- |
| Group | | |  |  |  |  |  |  |  |
|  | SBC | 0.00 |  |  |  | 1.00 |  |  |  |
|  | BCLC | 1.56 | 0.00 | 1.27 | 1.84 | 5.65 | 0.00 | 4.10 | 7.80 |
| Age | | 0.04 | 0.00 | 0.03 | 0.05 | 1.02 | 0.00 | 1.01 | 1.03 |
| Category | |  |  |  |  |  |  |  |  |
|  | >2005 | 0.00 |  |  |  | 1.00 |  |  |  |
|  | 1995-2005 | -0.17 | 0.73 | -0.57 | 0.24 | 0.92 | 0.87 | 0.60 | 1.41 |
|  | <1995 | -0.20 | 0.72 | -0.61 | 0.22 | 1.02 | 0.94 | 0.66 | 1.57 |
| Race | |  |  |  |  |  |  |  |  |
|  | NHW | 0.00 |  |  |  | 1.00 |  |  |  |
|  | NHB | 0.16 | 0.72 | -0.20 | 0.52 | 1.39 | 0.14 | 1.01 | 1.91 |
|  | NHA | 0.05 | 0.96 | -0.33 | 0.42 | 1.12 | 0.87 | 0.78 | 1.62 |
|  | Hispanic | 0.03 | 0.96 | -0.38 | 0.44 | 1.21 | 0.61 | 0.86 | 1.70 |
|  | Others | -0.12 | 0.96 | -2.10 | 1.87 | 0.92 | 0.87 | 0.51 | 1.65 |
| Insurance | |  |  |  |  |  |  |  |  |
|  | No | 0.00 |  |  |  | 1.00 |  |  |  |
|  | Yes | 0.27 | 0.96 | -1.72 | 2.25 | 1.89 | 0.87 | 0.21 | 17.12 |
|  | Unknown | -0.02 | 0.98 | -2.02 | 1.98 | 1.66 | 0.87 | 0.18 | 15.07 |
| Marital | |  |  |  |  |  |  |  |  |
|  | Single | 0.00 |  |  |  | 1.00 |  |  |  |
|  | Married | -0.15 | 0.67 | -0.42 | 0.11 | 0.95 | 0.87 | 0.73 | 1.24 |
|  | Unknown | -0.07 | 0.96 | -0.46 | 0.32 | 1.06 | 0.87 | 0.72 | 1.56 |
| SEER stage | |  |  |  |  |  |  |  |  |
|  | Localized | 0.00 |  |  |  | 1.00 |  |  |  |
|  | Regional | 0.60 | 0.00 | 0.28 | 0.92 | 1.85 | 0.00 | 1.33 | 2.57 |
|  | Distant | 1.45 | 0.08 | 0.21 | 2.68 | 4.07 | 0.27 | 0.80 | 20.67 |
|  | Unknown | -0.38 | 0.67 | -1.06 | 0.30 | 0.73 | 0.61 | 0.44 | 1.23 |
| Surgery | |  |  |  |  |  |  |  |  |
|  | No | 0.00 |  |  |  | 1.00 |  |  |  |
|  | Yes | -0.32 | 0.67 | -0.87 | 0.22 | 0.67 | 0.14 | 0.45 | 0.99 |
|  | Unknown | 0.39 | 0.96 | -1.72 | 2.50 | 1.29 | 0.89 | 0.11 | 14.75 |
| Radiation | |  |  |  |  |  |  |  |  |
|  | Yes | 0.00 |  |  |  | 1.00 |  |  |  |
|  | Unknown | -0.16 | 0.78 | -0.62 | 0.30 | 0.87 | 0.87 | 0.49 | 1.53 |
| _Cons | | -9.50 | 0.00 | -11.82 | -7.18 |  |  |  |  |
| /Ln_p | | 0.20 | 0.00 | 0.10 | 0.29 |  |  |  |  |

Incidence of all-cause and cancer specific death in BCLC was higher than that in the matched single bladder cancer. SBC: single bladder cancer; BCLC: bladder cancer with subsequent lung cancer; Coef.: regression coefficient; SHR: risk of cancer-specific death; p: adjusted p value.

Tab S9. All cause and cancer-specific death between categorized group with BCLC and matched single bladder cancer

|  |  | sTPC | | | | | | | | mTPC1 | | | | | | | | mTPC2 | | | | | | | |
| --- | --- | --- | --- | --- | --- | --- | --- | --- | --- | --- | --- | --- | --- | --- | --- | --- | --- | --- | --- | --- | --- | --- | --- | --- | --- |
|  |  | H.R | P | 95% CI | | SHR | P | 95% CI | | Coef. | P | 95% CI | | SHR | P | 95% CI | | H.R | P | 95% CI | | SHR | P | 95% CI | |
| Group | | |  |  |  |  |  |  |  |  |  |  |  |  |  |  |  |  |  |  |  |  |  |  |  |
|  | SBC | 1.00 |  |  |  | 1.00 |  |  |  | 0.00 |  |  |  | 1.00 |  |  |  | 1.00 |  |  |  | 1.00 |  |  |  |
|  | BCLC | 7.35 | 0.00 | 4.28 | 12.61 | 6.28 | 0.00 | 3.77 | 10.45 | 2.77 | 0.00 | 2.37 | 3.18 | 10.62 | 0.00 | 7.02 | 16.06 | 11.51 | 0.00 | 7.15 | 18.54 | 12.02 | 0.00 | 7.26 | 19.90 |
| Age | | 1.02 | 0.30 | 1.00 | 1.05 | 1.02 | 0.40 | 0.99 | 1.05 | 0.02 | 0.00 | 0.01 | 0.04 | 1.01 | 0.46 | 0.99 | 1.02 | 1.06 | 0.00 | 1.04 | 1.08 | 1.02 | 0.05 | 1.00 | 1.03 |
| Category | | |  |  |  |  |  |  |  |  |  |  |  |  |  |  |  |  |  |  |  |  |  |  |  |
|  | >2005 | 1.00 |  |  |  | 1.00 |  |  |  | 0.00 |  |  |  | 1.00 |  |  |  | 1.00 |  |  |  | 1.00 |  |  |  |
|  | 1995-2005 | 0.85 | 0.83 | 0.37 | 2.00 | 0.95 | 0.91 | 0.38 | 2.39 | 0.28 | 0.41 | -0.19 | 0.75 | 1.41 | 0.38 | 0.90 | 2.20 | 0.95 | 0.89 | 0.50 | 1.81 | 0.97 | 0.99 | 0.51 | 1.84 |
|  | <1995 | 1.73 | 0.54 | 0.70 | 4.26 | 1.99 | 0.41 | 0.71 | 5.53 | 0.09 | 0.85 | -0.44 | 0.61 | 1.49 | 0.38 | 0.91 | 2.44 | 0.72 | 0.55 | 0.37 | 1.37 | 0.93 | 0.99 | 0.49 | 1.74 |
| Race | | |  |  |  |  |  |  |  |  |  |  |  |  |  |  |  |  |  |  |  |  |  |  |  |
|  | NHW | 1.00 |  |  |  | 1.00 |  |  |  | 0.00 |  |  |  | 1.00 |  |  |  | 1.00 |  |  |  | 1.00 |  |  |  |
|  | NHB | 1.32 | 0.83 | 0.63 | 2.75 | 1.49 | 0.47 | 0.71 | 3.13 | -0.20 | 0.66 | -0.70 | 0.30 | 0.91 | 0.87 | 0.58 | 1.43 | 0.83 | 0.58 | 0.51 | 1.36 | 1.07 | 0.99 | 0.68 | 1.69 |
|  | NHA | 1.20 | 0.83 | 0.40 | 3.59 | 1.47 | 0.61 | 0.57 | 3.76 | 0.06 | 0.86 | -0.41 | 0.52 | 1.19 | 0.75 | 0.74 | 1.93 | 0.71 | 0.55 | 0.40 | 1.29 | 0.72 | 0.58 | 0.41 | 1.29 |
|  | Hispanic |  |  |  |  |  |  |  |  | 0.03 | 0.91 | -0.43 | 0.48 | 1.12 | 0.83 | 0.76 | 1.64 | 0.78 | 0.56 | 0.42 | 1.42 | 1.03 | 0.99 | 0.62 | 1.71 |
|  | Others |  |  |  |  |  |  |  |  |  |  |  |  |  |  |  |  | 1.90 | 0.56 | 0.44 | 8.23 | 2.44 | 0.48 | 0.65 | 9.18 |
| Insurance | | |  |  |  |  |  |  |  |  |  |  |  |  |  |  |  |  |  |  |  |  |  |  |  |
|  | No |  |  |  |  |  |  |  |  | 0.00 |  |  |  | 1.00 |  |  |  |  |  |  |  |  |  |  |  |
|  | Yes | 1.00 |  |  |  | 1.00 |  |  |  | -1.27 | 0.41 | -3.31 | 0.77 | 0.18 | 0.32 | 0.03 | 1.09 | 1.00 |  |  |  | 1.00 |  |  |  |
|  | Unknown | 0.88 | 0.83 | 0.38 | 2.01 | 0.92 | 0.91 | 0.37 | 2.34 | -1.54 | 0.36 | -3.62 | 0.54 | 0.15 | 0.32 | 0.03 | 0.94 | 0.69 | 0.55 | 0.33 | 1.45 | 1.00 | 1.00 | 0.44 | 2.26 |
| Marital | | |  |  |  |  |  |  |  |  |  |  |  |  |  |  |  |  |  |  |  |  |  |  |  |
|  | Single | 1.00 |  |  |  | 1.00 |  |  |  | 0.00 |  |  |  | 1.00 |  |  |  | 1.00 |  |  |  | 1.00 |  |  |  |
|  | Married | 0.90 | 0.83 | 0.56 | 1.46 | 0.76 | 0.47 | 0.47 | 1.24 | -0.28 | 0.23 | -0.59 | 0.04 | 1.04 | 0.88 | 0.76 | 1.43 | 1.03 | 0.89 | 0.70 | 1.53 | 1.22 | 0.62 | 0.82 | 1.82 |
|  | Unknown | 0.40 | 0.54 | 0.09 | 1.90 | 0.29 | 0.40 | 0.06 | 1.42 | -0.86 | 0.00 | -1.41 | -0.31 | 0.68 | 0.38 | 0.40 | 1.16 | 1.42 | 0.55 | 0.82 | 2.45 | 1.72 | 0.23 | 0.98 | 3.02 |
| SEER stage | | |  |  |  |  |  |  |  |  |  |  |  |  |  |  |  |  |  |  |  |  |  |  |  |
|  | Localized | 1.00 |  |  |  | 1.00 |  |  |  | 0.00 |  |  |  | 1.00 |  |  |  | 1.00 |  |  |  | 1.00 |  |  |  |
|  | Regional | 1.97 | 0.04 | 1.19 | 3.27 | 2.14 | 0.03 | 1.25 | 3.64 | 0.09 | 0.84 | -0.34 | 0.51 | 1.08 | 0.87 | 0.72 | 1.63 | 0.95 | 0.89 | 0.56 | 1.59 | 1.12 | 0.99 | 0.69 | 1.82 |
|  | Unknown | 0.99 | 0.99 | 0.22 | 4.45 | 0.89 | 0.90 | 0.34 | 2.35 | -0.26 | 0.73 | -1.14 | 0.61 | 0.62 | 0.38 | 0.31 | 1.26 | 0.41 | 0.26 | 0.16 | 1.08 | 0.56 | 0.48 | 0.23 | 1.34 |
| Surgery | | |  |  |  |  |  |  |  |  |  |  |  |  |  |  |  |  |  |  |  |  |  |  |  |
|  | No | 1.00 |  |  |  | 1.00 |  |  |  | 0.00 |  |  |  | 1.00 |  |  |  | 1.00 |  |  |  | 1.00 |  |  |  |
|  | Yes | 0.26 | 0.04 | 0.10 | 0.71 | 0.43 | 0.40 | 0.13 | 1.37 | 0.15 | 0.73 | -0.35 | 0.65 | 0.97 | 0.88 | 0.63 | 1.49 | 0.49 | 0.05 | 0.29 | 0.84 | 0.54 | 0.05 | 0.34 | 0.85 |
|  | Unknown |  |  |  |  |  |  |  |  | 0.92 | 0.41 | -0.60 | 2.45 | 2.44 | 0.38 | 0.64 | 9.39 |  |  |  |  |  |  |  |  |
| Radiation | | |  |  |  |  |  |  |  |  |  |  |  |  |  |  |  |  |  |  |  |  |  |  |  |
|  | Yes | 1.00 |  |  |  | 1.00 |  |  |  | 0.00 |  |  |  | 1.00 |  |  |  | 1.00 |  |  |  | 1.00 |  |  |  |
|  | Unknown | 0.75 | 0.83 | 0.29 | 1.92 | 1.20 | 0.91 | 0.39 | 3.73 | 0.70 | 0.20 | -0.02 | 1.42 | 0.93 | 0.88 | 0.44 | 1.96 | 0.58 | 0.55 | 0.22 | 1.53 | 1.16 | 0.99 | 0.37 | 3.58 |
| _Cons | |  |  |  |  |  |  |  |  | -10.01 | 0.00 | -12.58 | -7.44 |  |  |  |  |  |  |  |  |  |  |  |  |
| /Ln_p | |  |  |  |  |  |  |  |  | 0.44 | 0.00 | 0.33 | 0.54 |  |  |  |  |  |  |  |  |  |  |  |  |
| phtest | |  | 0.09 |  |  |  |  |  |  |  |  |  |  |  |  |  |  |  | 0.63 |  |  |  |  |  |  |

Incidence of all-cause and cancer specific death in categorized group with BCLC was higher than that in the matched single bladder cancer. SBC: single bladder cancer; BCLC: bladder cancer with subsequent lung cancer; Coef.: regression coefficient; H.R: risk of all-cause death; SHR: risk of cancer-specific death; p: adjusted p value.

Tab S10. All cause and cancer-specific death between LCPC and matched single lung cancer

|  |  | Coef. | p | 95% CI | | SHR | p | 95% CI | |
| --- | --- | --- | --- | --- | --- | --- | --- | --- | --- |
| Group | |  |  |  |  |  |  |  |  |
|  | SLC | 0.00 |  |  |  | 1.00 |  |  |  |
|  | LCPC | -1.31 | 0.00 | -1.46 | -1.15 | 0.40 | 0.00 | 0.35 | 0.47 |
| Race | |  |  |  |  |  |  |  |  |
|  | NHW | 0.00 |  |  |  | 1.00 |  |  |  |
|  | NHB | 0.08 | 0.59 | -0.09 | 0.26 | 1.07 | 0.62 | 0.88 | 1.30 |
|  | NHA | -0.28 | 0.20 | -0.61 | 0.05 | 0.69 | 0.10 | 0.49 | 0.98 |
|  | Hispanic | -0.09 | 0.83 | -0.43 | 0.25 | 0.86 | 0.52 | 0.62 | 1.20 |
| Marital | |  |  |  |  |  |  |  |  |
|  | Single | 0.00 |  |  |  | 1.00 |  |  |  |
|  | Married | 0.06 | 0.66 | -0.09 | 0.22 | 1.13 | 0.34 | 0.95 | 1.34 |
|  | Unknown | -0.07 | 0.84 | -0.52 | 0.38 | 0.86 | 0.62 | 0.53 | 1.41 |
| Insurance | |  |  |  |  |  |  |  |  |
|  | No | 0.00 |  |  |  | 1.00 |  |  |  |
|  | Yes | 0.08 | 0.89 | -1.06 | 1.23 | 1.29 | 0.68 | 0.39 | 4.28 |
|  | Unknown | 0.16 | 0.84 | -1.02 | 1.34 | 1.49 | 0.62 | 0.43 | 5.12 |
| SEER stage | |  |  |  |  |  |  |  |  |
|  | Localized | 0.00 |  |  |  | 1.00 |  |  |  |
|  | Regional | 0.86 | 0.00 | 0.65 | 1.07 | 1.99 | 0.00 | 1.64 | 2.41 |
|  | Distant | 0.56 | 0.00 | 0.32 | 0.80 | 1.35 | 0.06 | 1.05 | 1.72 |
|  | Unknown | 0.63 | 0.00 | 0.37 | 0.89 | 1.60 | 0.00 | 1.23 | 2.10 |
| Surgery | |  |  |  |  |  |  |  |  |
|  | No | 0.00 |  |  |  | 1.00 |  |  |  |
|  | Yes | -1.17 | 0.00 | -1.36 | -0.98 | 0.39 | 0.00 | 0.32 | 0.48 |
|  | Unknown | -0.10 | 0.84 | -0.64 | 0.44 | 0.69 | 0.45 | 0.35 | 1.37 |
| Radiation | |  |  |  |  |  |  |  |  |
|  | Unknown | 0.00 |  |  |  | 1.00 |  |  |  |
|  | Unknown | -0.09 | 0.49 | -0.25 | 0.07 | 0.90 | 0.45 | 0.76 | 1.08 |
| Category | |  |  |  |  |  |  |  |  |
|  | >2005 | 0.00 |  |  |  | 1.00 |  |  |  |
|  | 1995-2005 | 0.07 | 0.84 | -0.28 | 0.43 | 1.08 | 0.68 | 0.75 | 1.56 |
|  | <1995 | 0.29 | 0.24 | -0.07 | 0.66 | 1.30 | 0.34 | 0.89 | 1.90 |
| Age | | 0.03 | 0.00 | 0.02 | 0.04 | 1.01 | 0.03 | 1.00 | 1.02 |
| _Cons | | -4.75 | 0.00 | -6.06 | -3.45 |  |  |  |  |
| /Ln_p | | -0.22 | 0.00 | -0.27 | -0.16 |  |  |  |  |

Incidence of all-cause and cancer specific death in all LCPC, categorized group mTPC1 and mPTC2 were lower than that of matched single lung cancer. SLC: single lung cancer; LCPC: lung cancer with subsequent prostate cancer; Coef.: regression coefficient; SHR: risk of cancer-specific death; p: adjusted p value.

Tab S11. All cause and cancer-specific death between categorized group with LCPC and matched single lung cancer

|  |  | sTPC | | | | | | | | mTPC1 | | | | | | | | mTPC2 | | | | | | | |
| --- | --- | --- | --- | --- | --- | --- | --- | --- | --- | --- | --- | --- | --- | --- | --- | --- | --- | --- | --- | --- | --- | --- | --- | --- | --- |
|  |  | H.R | p | 95% CI | | SHR | p | 95% CI | | Coef. | p | 95% CI | | SHR | p | 95% CI | | Coef. | p | 95% CI | | SHR | p | 95% CI | |
| Group | | |  |  |  |  |  |  |  |  |  |  |  |  |  |  |  |  |  |  |  |  |  |  |  |
|  | SLC | 1.00 |  |  |  | 1.00 |  |  |  | 0.00 |  |  |  | 1.00 |  |  |  | 0.00 |  |  |  | 1.00 |  |  |  |
|  | LCPC | 0.97 | 0.81 | 0.79 | 1.19 | 1.04 | 0.74 | 0.84 | 1.27 | -1.40 | 0.00 | -1.62 | -1.18 | 0.32 | 0.00 | 0.26 | 0.40 | -0.90 | 0.00 | -1.35 | -0.44 | 0.54 | 0.13 | 0.35 | 0.85 |
| Race | | |  |  |  |  |  |  |  |  |  |  |  |  |  |  |  |  |  |  |  |  |  |  |  |
|  | NHW | 1.00 |  |  |  | 1.00 |  |  |  | 0.00 |  |  |  | 1.00 |  |  |  | 0.00 |  |  |  | 1.00 |  |  |  |
|  | NHB | 1.32 | 0.11 | 1.01 | 1.71 | 1.34 | 0.08 | 1.04 | 1.74 | -0.08 | 0.62 | -0.35 | 0.19 | 0.95 | 0.87 | 0.71 | 1.28 | 0.52 | 0.14 | 0.00 | 1.03 | 1.45 | 0.55 | 0.85 | 2.45 |
|  | NHA | 0.75 | 0.44 | 0.47 | 1.22 | 0.62 | 0.10 | 0.39 | 0.99 | 0.28 | 0.32 | -0.13 | 0.70 | 1.37 | 0.12 | 0.97 | 1.91 | -0.87 | 0.16 | -1.81 | 0.08 | 0.52 | 0.55 | 0.22 | 1.25 |
|  | Hispanic | 0.87 | 0.79 | 0.52 | 1.47 | 0.90 | 0.69 | 0.57 | 1.42 | 0.17 | 0.62 | -0.38 | 0.72 | 1.04 | 0.93 | 0.62 | 1.77 |  |  |  |  |  |  |  |  |
| Marital | | | |  |  |  |  |  |  |  |  |  |  |  |  |  |  |  |  |  |  |  |  |  |  |
|  | Single | 1.00 |  |  |  | 1.00 |  |  |  | 0.00 |  |  |  | 1.00 |  |  |  | 0.00 |  |  |  | 1.00 |  |  |  |
|  | Married | 1.08 | 0.74 | 0.86 | 1.35 | 1.07 | 0.66 | 0.86 | 1.35 | -0.16 | 0.32 | -0.40 | 0.08 | 0.95 | 0.87 | 0.73 | 1.24 | 0.13 | 0.77 | -0.43 | 0.68 | 1.19 | 0.85 | 0.69 | 2.06 |
|  | Unknown | 1.02 | 0.96 | 0.51 | 2.05 | 0.73 | 0.61 | 0.34 | 1.56 | -0.08 | 0.84 | -0.83 | 0.68 | 1.42 | 0.58 | 0.67 | 2.98 |  |  |  |  |  |  |  |  |
| Insurance | | | |  |  |  |  |  |  |  |  |  |  |  |  |  |  |  |  |  |  |  |  |  |  |
|  | No |  |  |  |  |  |  |  |  |  |  |  |  |  |  |  |  |  |  |  |  |  |  |  |  |
|  | Yes | 1.00 |  |  |  | 1.00 |  |  |  | 0.00 |  |  |  | 1.00 |  |  |  | 0.00 |  |  |  | 1.00 |  |  |  |
|  | Unknown | 1.15 | 0.79 | 0.64 | 2.07 | 1.29 | 0.54 | 0.76 | 2.19 | 0.07 | 0.84 | -0.44 | 0.57 | 1.01 | 0.97 | 0.59 | 1.74 | 0.22 | 0.81 | -1.14 | 1.59 | 0.79 | 0.86 | 0.18 | 3.38 |
| SEER stage | | | |  |  |  |  |  |  |  |  |  |  |  |  |  |  |  |  |  |  |  |  |  |  |
|  | Localized | 1.00 |  |  |  | 1.00 |  |  |  | 0.00 |  |  |  | 1.00 |  |  |  | 0.00 |  |  |  | 1.00 |  |  |  |
|  | Regional | 2.58 | 0.00 | 1.76 | 3.77 | 2.15 | 0.00 | 1.51 | 3.04 | 0.87 | 0.00 | 0.60 | 1.14 | 2.12 | 0.00 | 1.64 | 2.73 | 0.20 | 0.77 | -0.57 | 0.98 | 0.85 | 0.86 | 0.39 | 1.87 |
|  | Distant | 1.72 | 0.03 | 1.17 | 2.52 | 1.51 | 0.08 | 1.04 | 2.18 | 0.52 | 0.02 | 0.13 | 0.91 | 1.58 | 0.08 | 1.06 | 2.34 | 0.62 | 0.42 | -0.41 | 1.65 | 1.39 | 0.85 | 0.44 | 4.34 |
|  | Unknown | 2.58 | 0.00 | 1.72 | 3.88 | 2.22 | 0.00 | 1.51 | 3.27 | 0.59 | 0.00 | 0.23 | 0.95 | 1.42 | 0.12 | 0.97 | 2.07 | 0.20 | 0.77 | -0.46 | 0.85 | 1.19 | 0.85 | 0.63 | 2.27 |
| Surgery | | |  |  |  |  |  |  |  |  |  |  |  |  |  |  |  |  |  |  |  |  |  |  |  |
|  | No | 1.00 |  |  |  | 1.00 |  |  |  | 0.00 |  |  |  | 1.00 |  |  |  | 0.00 |  |  |  | 1.00 |  |  |  |
|  | Yes | 0.34 | 0.00 | 0.25 | 0.46 | 0.42 | 0.00 | 0.31 | 0.57 | -0.91 | 0.00 | -1.18 | -0.65 | 0.53 | 0.00 | 0.40 | 0.70 | -0.83 | 0.14 | -1.67 | 0.00 | 0.42 | 0.39 | 0.17 | 1.02 |
|  | Unknown | 0.63 | 0.37 | 0.33 | 1.20 | 0.44 | 0.09 | 0.20 | 0.97 | 0.76 | 0.23 | -0.17 | 1.69 | 2.24 | 0.12 | 0.96 | 5.24 |  |  |  |  |  |  |  |  |
| Radiation | | |  |  |  |  |  |  |  |  |  |  |  |  |  |  |  |  |  |  |  |  |  |  |  |
|  | Yes | 1.00 |  |  |  | 1.00 |  |  |  | 0.00 |  |  |  | 1.00 |  |  |  | 0.00 |  |  |  | 1.00 |  |  |  |
|  | Unknown | 0.96 | 0.81 | 0.77 | 1.20 | 0.83 | 0.14 | 0.66 | 1.03 | -0.14 | 0.34 | -0.39 | 0.11 | 0.91 | 0.68 | 0.70 | 1.18 | -0.02 | 0.94 | -0.66 | 0.61 | 0.95 | 0.86 | 0.50 | 1.78 |
| Category | | |  |  |  |  |  |  |  |  |  |  |  |  |  |  |  |  |  |  |  |  |  |  |  |
|  | >2005 | 1.00 |  |  |  | 1.00 |  |  |  | 0.00 |  |  |  | 1.00 |  |  |  | 0.00 |  |  |  | 1.00 |  |  |  |
|  | 1995-2005 | 1.31 | 0.56 | 0.74 | 2.33 | 1.18 | 0.66 | 0.70 | 1.98 | 0.27 | 0.34 | -0.21 | 0.75 | 1.70 | 0.11 | 1.02 | 2.84 | -0.92 | 0.32 | -2.19 | 0.36 | 0.58 | 0.85 | 0.14 | 2.35 |
|  | <1995 | 1.46 | 0.40 | 0.81 | 2.62 | 1.13 | 0.69 | 0.67 | 1.91 | 0.31 | 0.34 | -0.20 | 0.83 | 1.83 | 0.10 | 1.07 | 3.13 | -0.64 | 0.54 | -1.96 | 0.69 | 0.88 | 0.86 | 0.21 | 3.59 |
| Age | | 1.02 | 0.00 | 1.01 | 1.03 | 1.02 | 0.04 | 1.00 | 1.03 | 0.02 | 0.02 | 0.00 | 0.03 | 1.00 | 0.87 | 0.99 | 1.01 | 0.05 | 0.00 | 0.03 | 0.08 | 1.02 | 0.57 | 0.99 | 1.04 |
| _Cons | |  |  |  |  |  |  |  |  | -5.54 | 0.00 | -6.60 | -4.49 |  |  |  |  | -13.98 | 0.00 | -17.08 | -10.88 |  |  |  |  |
| /Ln_p | |  |  |  |  |  |  |  |  | 0.15 | 0.00 | 0.08 | 0.23 |  |  |  |  | 0.74 | 0.00 | 0.57 | 0.92 |  |  |  |  |
| phtest | |  | 0.25 |  |  |  |  |  |  |  |  |  |  |  |  |  |  |  |  |  |  |  |  |  |  |

Incidence of all-cause and cancer specific death in group mTPC1, and incidence of all-cause in group mPTC2 with LCPC were lower than that of matched single lung cancer. SLC: single lung cancer; LCPC: lung cancer with subsequent prostate cancer; H.R: risk of all-cause death; Coef.: regression coefficient; SHR: risk of cancer-specific death; p: adjusted p value.

Tab S11. All cause and cancer-specific death between LCPC and matched single prostate cancer

|  |  | Coef. | p | 95% CI | | SHR | p | 95% CI | |
| --- | --- | --- | --- | --- | --- | --- | --- | --- | --- |
| Group | |  |  |  |  |  |  |  |  |
|  | SPC | 0.00 |  |  |  | 1.00 |  |  |  |
|  | LCPC | 0.79 | 0.00 | 0.45 | 1.12 | 2.12 | 0.00 | 1.51 | 2.97 |
| Race | |  |  |  |  |  |  |  |  |
|  | NHW | 0.00 |  |  |  | 1.00 |  |  |  |
|  | NHB | 0.25 | 0.10 | 0.00 | 0.50 | 1.20 | 0.27 | 0.94 | 1.53 |
|  | NHA | -0.16 | 0.52 | -0.55 | 0.24 | 0.88 | 0.72 | 0.58 | 1.33 |
|  | Hispanic | -0.11 | 0.62 | -0.56 | 0.33 | 0.80 | 0.54 | 0.50 | 1.29 |
|  | Others | 1.27 | 0.13 | -0.14 | 2.67 | 3.02 | 0.02 | 1.29 | 7.03 |
| Marital | |  |  |  |  |  |  |  |  |
|  | Single | 0.00 |  |  |  | 1.00 |  |  |  |
|  | Married | -0.13 | 0.37 | -0.36 | 0.11 | 0.94 | 0.74 | 0.74 | 1.19 |
|  | Unknown | -0.12 | 0.55 | -0.46 | 0.22 | 0.93 | 0.78 | 0.66 | 1.31 |
| Insurance | |  |  |  |  |  |  |  |  |
|  | No | 0.00 |  |  |  | 1.00 |  |  |  |
|  | Yes | -1.37 | 0.10 | -2.78 | 0.05 | 0.27 | 0.02 | 0.10 | 0.71 |
|  | Unknown | -1.47 | 0.10 | -2.91 | -0.03 | 0.27 | 0.02 | 0.10 | 0.74 |
| SEER stage | |  |  |  |  |  |  |  |  |
|  | Localized | 0.00 |  |  |  | 1.00 |  |  |  |
|  | Distant | 2.49 | 0.00 | 1.94 | 3.05 | 9.58 | 0.00 | 5.83 | 15.74 |
|  | Unknown | 1.35 | 0.00 | 0.87 | 1.83 | 3.15 | 0.00 | 1.96 | 5.08 |
| Surgery | |  |  |  |  |  |  |  |  |
|  | No | 0.00 |  |  |  | 1.00 |  |  |  |
|  | Yes | -0.42 | 0.00 | -0.67 | -0.17 | 0.68 | 0.00 | 0.52 | 0.88 |
|  | Unknown | 0.42 | 0.20 | -0.13 | 0.97 | 1.20 | 0.72 | 0.66 | 2.18 |
| Radiation | |  |  |  |  |  |  |  |  |
|  | No | 0.00 |  |  |  | 1.00 |  |  |  |
|  | Yes | -0.76 | 0.20 | -1.79 | 0.28 | 0.58 | 0.54 | 0.18 | 1.88 |
|  | Unknown | -0.27 | 0.62 | -1.29 | 0.75 | 0.89 | 0.85 | 0.28 | 2.90 |
| Category | |  |  |  |  |  |  |  |  |
|  | >2005 | 0.00 |  |  |  | 1.00 |  |  |  |
|  | 1995-2005 | 0.51 | 0.02 | 0.12 | 0.90 | 1.80 | 0.00 | 1.20 | 2.70 |
|  | <1995 | 0.71 | 0.00 | 0.28 | 1.14 | 2.07 | 0.00 | 1.30 | 3.28 |
| Age | | 0.02 | 0.02 | 0.00 | 0.03 | 1.00 | 0.85 | 0.99 | 1.01 |
| _Cons | | -5.47 | 0.00 | -7.43 | -3.52 |  |  |  |  |
| /Ln_p | | -0.21 | 0.00 | -0.30 | -0.13 |  |  |  |  |

Incidence of all-cause and cancer specific death in LCPC were higher than that of matched single prostate cancer. SPC: single prostate cancer; LCPC: lung cancer with subsequent prostate cancer; Coef.: regression coefficient; SHR: risk of cancer-specific death; p: adjusted p value.

Tab S12. All cause and cancer-specific death between categorized group with LCPC and matched single prostate cancer

|  |  | sTPC | | | | | | | | mTPC1 | | | | | | | | mTPC2 | | | | | | | |
| --- | --- | --- | --- | --- | --- | --- | --- | --- | --- | --- | --- | --- | --- | --- | --- | --- | --- | --- | --- | --- | --- | --- | --- | --- | --- |
|  |  | Coef. | p | 95% CI | | SHR | p | 95% CI | | H.R | p | 95% CI | | SHR | p | 95% CI | | H.R | p | 95% CI | | SHR | p | 95% CI | |
| Group | | |  |  |  |  |  |  |  |  |  |  |  |  |  |  |  |  |  |  |  |  |  |  |  |
|  | SPC | 0.00 |  |  |  | 1.00 |  |  |  | 1.00 |  |  |  | 1.00 |  |  |  | 1.00 |  |  |  | 1.00 |  |  |  |
|  | LCPC | 2.05 | 0.00 | 1.60 | 2.49 | 5.16 | 0.00 | 3.25 | 8.18 | 1.75 | 0.06 | 1.11 | 2.74 | 1.80 | 0.03 | 1.15 | 2.81 | 1.48 | 0.53 | 0.72 | 3.01 | 1.11 | 0.98 | 0.53 | 2.34 |
| Race | | |  |  |  |  |  |  |  |  |  |  |  |  |  |  |  |  |  |  |  |  |  |  |  |
|  | NHW | 0.00 |  |  |  | 1.00 |  |  |  | 1.00 |  |  |  | 1.00 |  |  |  | 1.00 |  |  |  | 1.00 |  |  |  |
|  | NHB | 0.21 | 0.41 | -0.13 | 0.55 | 1.15 | 0.65 | 0.83 | 1.61 | 1.38 | 0.17 | 0.95 | 1.99 | 1.34 | 0.25 | 0.92 | 1.95 | 1.50 | 0.35 | 0.87 | 2.59 | 1.49 | 0.40 | 0.85 | 2.62 |
|  | NHA | 0.16 | 0.75 | -0.40 | 0.72 | 0.95 | 0.91 | 0.52 | 1.74 | 0.80 | 0.60 | 0.45 | 1.44 | 0.79 | 0.64 | 0.43 | 1.45 | 0.14 | 0.19 | 0.02 | 1.01 | 0.17 | 0.30 | 0.02 | 1.25 |
|  | Hispanic | -0.09 | 0.84 | -0.71 | 0.52 | 0.87 | 0.81 | 0.45 | 1.65 | 1.23 | 0.68 | 0.62 | 2.44 | 1.04 | 0.92 | 0.52 | 2.07 | 1.17 | 0.86 | 0.34 | 3.98 | 0.97 | 0.98 | 0.25 | 3.70 |
|  | Others |  |  |  |  |  |  |  |  | 4.03 | 0.13 | 0.97 | 16.84 | 3.92 | 0.03 | 1.50 | 10.24 |  |  |  |  |  |  |  |  |
| Marital | | | |  |  |  |  |  |  |  |  |  |  |  |  |  |  |  |  |  |  |  |  |  |  |
|  | Single | 0.00 |  |  |  | 1.00 |  |  |  | 1.00 |  |  |  | 1.00 |  |  |  | 1.00 |  |  |  | 1.00 |  |  |  |
|  | Married | -0.06 | 0.84 | -0.37 | 0.25 | 1.05 | 0.86 | 0.76 | 1.45 | 0.83 | 0.40 | 0.60 | 1.16 | 0.84 | 0.46 | 0.61 | 1.17 | 1.13 | 0.84 | 0.63 | 2.03 | 1.51 | 0.41 | 0.82 | 2.76 |
|  | Unknown | 0.02 | 0.94 | -0.49 | 0.53 | 0.97 | 0.91 | 0.57 | 1.64 | 1.00 | 1.00 | 0.62 | 1.62 | 1.08 | 0.87 | 0.66 | 1.78 | 0.76 | 0.84 | 0.29 | 2.01 | 0.97 | 0.98 | 0.32 | 2.89 |
| Insurance | | | |  |  |  |  |  |  |  |  |  |  |  |  |  |  |  |  |  |  |  |  |  |  |
|  | No | 0.00 |  |  |  | 1.00 |  |  |  |  |  |  |  |  |  |  |  |  |  |  |  |  |  |  |  |
|  | Yes | -0.70 | 0.53 | -2.15 | 0.75 | 0.54 | 0.32 | 0.24 | 1.19 | 1.00 |  |  |  | 1.00 |  |  |  | 1.00 |  |  |  | 1.00 |  |  |  |
|  | Unknown | -0.59 | 0.61 | -2.08 | 0.91 | 0.71 | 0.65 | 0.30 | 1.67 | 0.70 | 0.40 | 0.36 | 1.33 | 0.68 | 0.43 | 0.35 | 1.32 | 1.71 | 0.53 | 0.67 | 4.32 | 1.70 | 0.51 | 0.66 | 4.41 |
| SEER stage | | | |  |  |  |  |  |  |  |  |  |  |  |  |  |  |  |  |  |  |  |  |  |  |
|  | Localized | 0.00 |  |  |  | 1.00 |  |  |  | 1.00 |  |  |  | 1.00 |  |  |  | 1.00 |  |  |  | 1.00 |  |  |  |
|  | Distant | 2.33 | 0.00 | 1.59 | 3.08 | 7.74 | 0.00 | 3.80 | 15.77 | 5.30 | 0.00 | 1.94 | 14.49 | 4.43 | 0.03 | 1.55 | 12.66 | 8.34 | 0.00 | 2.41 | 28.90 | 6.82 | 0.00 | 1.88 | 24.69 |
|  | Unknown | 1.09 | 0.00 | 0.43 | 1.75 | 2.90 | 0.00 | 1.47 | 5.73 | 4.60 | 0.00 | 2.40 | 8.81 | 3.71 | 0.00 | 1.96 | 7.03 | 3.15 | 0.10 | 1.19 | 8.32 | 3.64 | 0.05 | 1.30 | 10.16 |
| Surgery | | | |  |  |  |  |  |  |  |  |  |  |  |  |  |  |  |  |  |  |  |  |  |  |
|  | No | 0.00 |  |  |  | 1.00 |  |  |  | 1.00 |  |  |  | 1.00 |  |  |  | 1.00 |  |  |  | 1.00 |  |  |  |
|  | Yes | -0.59 | 0.00 | -0.92 | -0.26 | 0.69 | 0.09 | 0.48 | 0.97 | 0.56 | 0.00 | 0.40 | 0.80 | 0.66 | 0.07 | 0.45 | 0.96 | 1.56 | 0.30 | 0.92 | 2.67 | 1.57 | 0.33 | 0.91 | 2.71 |
|  | Unknown | -0.42 | 0.51 | -1.22 | 0.38 | 0.69 | 0.65 | 0.26 | 1.84 | 2.39 | 0.12 | 1.02 | 5.59 | 2.22 | 0.17 | 0.91 | 5.41 | 1.62 | 0.84 | 0.21 | 12.59 | 0.97 | 0.98 | 0.09 | 10.29 |
| Radiation | | | |  |  |  |  |  |  |  |  |  |  |  |  |  |  |  |  |  |  |  |  |  |  |
|  | No | 0.00 |  |  |  | 1.00 |  |  |  | 1.00 |  |  |  | 1.00 |  |  |  |  |  |  |  |  |  |  |  |
|  | Yes | -3.04 | 0.00 | -4.54 | -1.53 | 0.10 | 0.00 | 0.02 | 0.43 | 0.94 | 1.00 | 0.22 | 4.10 | 1.23 | 0.87 | 0.31 | 4.90 | 1.00 |  |  |  | 1.00 |  |  |  |
|  | Unknown | -2.35 | 0.00 | -3.82 | -0.89 | 0.16 | 0.07 | 0.04 | 0.71 | 1.42 | 0.73 | 0.33 | 6.09 | 1.62 | 0.64 | 0.40 | 6.52 | 0.91 | 0.84 | 0.54 | 1.54 | 0.92 | 0.98 | 0.54 | 1.58 |
| Category | | | |  |  |  |  |  |  |  |  |  |  |  |  |  |  |  |  |  |  |  |  |  |  |
|  | >2005 | 0.00 |  |  |  | 1.00 |  |  |  | 1.00 |  |  |  | 1.00 |  |  |  | 1.00 |  |  |  | 1.00 |  |  |  |
|  | 1995-2005 | 0.07 | 0.84 | -0.45 | 0.59 | 1.12 | 0.81 | 0.66 | 1.89 | 2.29 | 0.03 | 1.23 | 4.25 | 2.62 | 0.00 | 1.40 | 4.90 | 0.86 | 0.84 | 0.37 | 1.99 | 0.90 | 0.98 | 0.38 | 2.13 |
|  | <1995 | 0.51 | 0.18 | -0.07 | 1.09 | 1.34 | 0.65 | 0.73 | 2.47 | 3.12 | 0.00 | 1.64 | 5.95 | 3.17 | 0.00 | 1.63 | 6.14 | 1.01 | 0.98 | 0.39 | 2.62 | 0.94 | 0.98 | 0.33 | 2.65 |
| Age | | 0.02 | 0.02 | 0.01 | 0.04 | 1.01 | 0.65 | 0.99 | 1.02 | 1.01 | 0.31 | 0.99 | 1.03 | 1.00 | 0.92 | 0.98 | 1.02 | 1.08 | 0.00 | 1.04 | 1.11 | 1.05 | 0.00 | 1.02 | 1.09 |
| _Cons | | -4.53 | 0.00 | -6.89 | -2.17 |  |  |  |  |  |  |  |  |  |  |  |  |  |  |  |  |  |  |  |  |
| /Ln_p | | -0.15 | 0.00 | -0.25 | -0.05 |  |  |  |  |  |  |  |  |  |  |  |  |  |  |  |  |  |  |  |  |
| phtest | |  |  |  |  |  |  |  |  |  | 0.06 |  |  |  |  |  |  |  |  |  | 0.95 |  |  |  |  |

Incidence of all-cause and cancer specific death in group sTPC1, and cancer specific death in group mPTC1 were higher than that of matched single prostate cancer. SPC: single prostate cancer; LCPC: lung cancer with subsequent prostate cancer; H.R: risk of all-cause death; Coef.: regression coefficient; SHR: risk of cancer-specific death; p: adjusted p value.

Tab S13.All cause and cancer-specific death between PCLC and matched single lung cancer

|  |  | Coef. | p | 95% CI | | SHR | p | 95% CI | |
| --- | --- | --- | --- | --- | --- | --- | --- | --- | --- |
| Group | | |  |  |  |  |  |  |  |
|  | SLC | 0.00 |  |  |  | 1.00 |  |  |  |
|  | PCLC | -0.32 | 0.00 | -0.43 | -0.22 | 0.77 | 0.00 | 0.69 | 0.85 |
| Race | | |  |  |  |  |  |  |  |
|  | NHW | 0.00 |  |  |  | 1.00 |  |  |  |
|  | NHB | 0.04 | 0.51 | -0.08 | 0.17 | 0.98 | 0.85 | 0.87 | 1.11 |
|  | NHA | -0.21 | 0.04 | -0.39 | -0.04 | 0.87 | 0.17 | 0.73 | 1.03 |
|  | Hispanic | -0.14 | 0.24 | -0.33 | 0.06 | 0.91 | 0.42 | 0.75 | 1.10 |
|  | Others | -0.30 | 0.51 | -1.10 | 0.51 | 0.70 | 0.40 | 0.37 | 1.33 |
| Marital | | |  |  |  |  |  |  |  |
|  | Single | 0.00 |  |  |  | 1.00 |  |  |  |
|  | Married | -0.09 | 0.15 | -0.20 | 0.02 | 0.96 | 0.53 | 0.86 | 1.07 |
|  | Unknown | -0.07 | 0.51 | -0.26 | 0.12 | 1.02 | 0.91 | 0.84 | 1.23 |
| SEER stage | | |  |  |  |  |  |  |  |
|  | Localized | 0.00 |  |  |  | 1.00 |  |  |  |
|  | Regional | 0.75 | 0.00 | 0.61 | 0.88 | 1.98 | 0.00 | 1.73 | 2.25 |
|  | Distant | 0.74 | 0.00 | 0.58 | 0.90 | 1.97 | 0.00 | 1.69 | 2.30 |
|  | Unknown | 0.67 | 0.00 | 0.49 | 0.86 | 1.59 | 0.00 | 1.33 | 1.91 |
| Surgery | | |  |  |  |  |  |  |  |
|  | No | 0.00 |  |  |  | 1.00 |  |  |  |
|  | Yes | -1.15 | 0.00 | -1.28 | -1.02 | 0.44 | 0.00 | 0.39 | 0.49 |
|  | Unknown | 0.25 | 0.13 | -0.03 | 0.52 | 1.38 | 0.07 | 1.02 | 1.85 |
| Radiation | | |  |  |  |  |  |  |  |
|  | No | 0.00 |  |  |  | 1.00 |  |  |  |
|  | Yes | -0.75 | 0.51 | -2.72 | 1.22 | 0.48 | 0.00 | 0.41 | 0.58 |
|  | Unknown | -0.69 | 0.51 | -2.66 | 1.28 | 0.50 | 0.00 | 0.41 | 0.60 |
| Category | | |  |  |  |  |  |  |  |
|  | >2005 | 0.00 |  |  |  | 1.00 |  |  |  |
|  | 1995-2005 | 0.29 | 0.00 | 0.16 | 0.41 | 1.38 | 0.00 | 1.23 | 1.56 |
|  | <1995 | 0.44 | 0.00 | 0.30 | 0.58 | 1.53 | 0.00 | 1.32 | 1.77 |
| Age | | 0.01 | 0.00 | 0.01 | 0.02 | 1.00 | 0.91 | 0.99 | 1.01 |
| _Cons | | -2.98 | 0.00 | -5.00 | -0.96 |  |  |  |  |
| /Ln_p | | -0.16 | 0.00 | -0.19 | -0.12 |  |  |  |  |

Incidence of all-cause and cancer specific death in PCLC were lower than that of matched single lung cancer. SLC: single lung cancer; PCLC: prostate cancer with subsequent lung cancer; Coef.: regression coefficient; SHR: risk of cancer-specific death; p: adjusted p value.

Tab S14.All cause and cancer-specific death between categorized group with PCLC and matched single lung cancer

|  |  | sTPC | | | | | | | | mTPC1 | | | | | | | | mTPC2 | | | | | | | |
| --- | --- | --- | --- | --- | --- | --- | --- | --- | --- | --- | --- | --- | --- | --- | --- | --- | --- | --- | --- | --- | --- | --- | --- | --- | --- |
|  |  | Coef. | p | 95% CI | | SHR | p | 95% CI | | Coef. | p | 95% CI | | SHR | p | 95% CI | | Coef. | p | 95% CI | | SHR | p | 95% CI | |
| Group | | | |  |  |  |  |  |  |  |  |  |  |  |  |  |  |  |  |  |  |  |  |  |  |
|  | SLC | 0.00 |  |  |  | 1.00 |  |  |  | 0.00 |  |  |  | 1.00 |  |  |  | 0.00 |  |  |  | 1.00 |  |  |  |
|  | PCLC | -0.24 | 0.02 | -0.41 | -0.07 | 0.83 | 0.08 | 0.70 | 0.98 | -0.18 | 0.00 | -0.30 | -0.06 | 0.85 | 0.02 | 0.75 | 0.95 | -0.28 | 0.00 | -0.40 | -0.15 | 0.84 | 0.03 | 0.74 | 0.95 |
| Race | | | |  |  |  |  |  |  |  |  |  |  |  |  |  |  |  |  |  |  |  |  |  |  |
|  | NHW | 0.00 |  |  |  | 1.00 |  |  |  | 0.00 |  |  |  | 1.00 |  |  |  | 0.00 |  |  |  | 1.00 |  |  |  |
|  | NHB | -0.09 | 0.47 | -0.30 | 0.11 | 0.88 | 0.35 | 0.72 | 1.08 | -0.05 | 0.59 | -0.20 | 0.09 | 0.96 | 0.81 | 0.83 | 1.12 | 0.00 | 0.99 | -0.16 | 0.16 | 0.91 | 0.43 | 0.78 | 1.07 |
|  | NHA | -0.37 | 0.06 | -0.72 | -0.02 | 0.85 | 0.44 | 0.60 | 1.20 | -0.28 | 0.02 | -0.49 | -0.08 | 0.81 | 0.09 | 0.66 | 1.00 | -0.33 | 0.00 | -0.56 | -0.10 | 0.86 | 0.34 | 0.69 | 1.08 |
|  | Hispanic | -0.39 | 0.06 | -0.75 | -0.02 | 0.71 | 0.15 | 0.49 | 1.04 | -0.06 | 0.67 | -0.28 | 0.16 | 1.02 | 0.91 | 0.82 | 1.27 | -0.03 | 0.84 | -0.25 | 0.19 | 0.94 | 0.69 | 0.75 | 1.18 |
|  | Others |  |  |  |  |  |  |  |  | 0.50 | 0.59 | -0.90 | 1.90 | 1.62 | 0.51 | 0.62 | 4.28 | -0.13 | 0.78 | -0.80 | 0.53 | 0.94 | 0.82 | 0.56 | 1.57 |
| Marital | | | |  |  |  |  |  |  |  |  |  |  |  |  |  |  |  |  |  |  |  |  |  |  |
|  | Single | 0.00 |  |  |  | 1.00 |  |  |  | 0.00 |  |  |  | 1.00 |  |  |  | 0.00 |  |  |  | 1.00 |  |  |  |
|  | Married | -0.06 | 0.55 | -0.24 | 0.12 | 0.94 | 0.57 | 0.78 | 1.14 | -0.07 | 0.43 | -0.19 | 0.06 | 1.00 | 0.94 | 0.88 | 1.13 | -0.09 | 0.28 | -0.22 | 0.04 | 1.02 | 0.82 | 0.89 | 1.16 |
|  | Unknown | 0.02 | 0.94 | -0.38 | 0.42 | 1.13 | 0.53 | 0.82 | 1.55 | -0.03 | 0.81 | -0.28 | 0.22 | 1.04 | 0.91 | 0.80 | 1.35 | 0.10 | 0.51 | -0.13 | 0.34 | 1.13 | 0.47 | 0.89 | 1.42 |
| SEER stage | | | |  |  |  |  |  |  |  |  |  |  |  |  |  |  |  |  |  |  |  |  |  |  |
|  | Localized | 0.00 |  |  |  | 1.00 |  |  |  | 0.00 |  |  |  | 1.00 |  |  |  | 0.00 |  |  |  | 1.00 |  |  |  |
|  | Regional | 0.99 | 0.00 | 0.74 | 1.25 | 2.12 | 0.00 | 1.67 | 2.69 | 0.87 | 0.00 | 0.71 | 1.04 | 2.24 | 0.00 | 1.91 | 2.62 | 0.80 | 0.00 | 0.63 | 0.96 | 1.98 | 0.00 | 1.69 | 2.32 |
|  | Distant | 1.05 | 0.00 | 0.78 | 1.32 | 2.29 | 0.00 | 1.80 | 2.92 | 0.89 | 0.00 | 0.72 | 1.06 | 2.27 | 0.00 | 1.92 | 2.67 | 0.68 | 0.00 | 0.50 | 0.87 | 1.84 | 0.00 | 1.53 | 2.20 |
|  | Unknown | 1.11 | 0.00 | 0.77 | 1.46 | 2.35 | 0.00 | 1.70 | 3.25 | 0.78 | 0.00 | 0.55 | 1.00 | 2.00 | 0.00 | 1.59 | 2.51 | 0.60 | 0.00 | 0.36 | 0.83 | 1.35 | 0.03 | 1.07 | 1.70 |
| Surgery | | | |  |  |  |  |  |  |  |  |  |  |  |  |  |  |  |  |  |  |  |  |  |  |
|  | No | 0.00 |  |  |  | 1.00 |  |  |  | 0.00 |  |  |  | 1.00 |  |  |  | 0.00 |  |  |  | 1.00 |  |  |  |
|  | Yes | -1.03 | 0.00 | -1.26 | -0.81 | 0.48 | 0.00 | 0.39 | 0.60 | -1.14 | 0.00 | -1.29 | -0.99 | 0.45 | 0.00 | 0.39 | 0.51 | -1.11 | 0.00 | -1.27 | -0.95 | 0.45 | 0.00 | 0.39 | 0.52 |
|  | Unknown | 0.21 | 0.47 | -0.30 | 0.73 | 1.33 | 0.41 | 0.78 | 2.26 | -0.10 | 0.67 | -0.50 | 0.30 | 0.94 | 0.91 | 0.58 | 1.53 | 0.21 | 0.51 | -0.24 | 0.67 | 1.36 | 0.26 | 0.91 | 2.02 |
| Radiation | | | |  |  |  |  |  |  |  |  |  |  |  |  |  |  |  |  |  |  |  |  |  |  |
|  | No |  |  |  |  |  |  |  |  | 0.00 |  |  |  | 1.00 |  |  |  |  |  |  |  |  |  |  |  |
|  | Yes | 0.00 |  |  |  | 1.00 |  |  |  | -0.80 | 0.20 | -1.79 | 0.20 | 0.50 | 0.00 | 0.37 | 0.68 | 0.00 |  |  |  | 1.00 |  |  |  |
|  | Unknown | -0.12 | 0.24 | -0.30 | 0.06 | 0.89 | 0.33 | 0.74 | 1.06 | -0.73 | 0.23 | -1.72 | 0.26 | 0.53 | 0.00 | 0.39 | 0.72 | 0.03 | 0.78 | -0.10 | 0.17 | 0.96 | 0.69 | 0.84 | 1.10 |
| Category | | | |  |  |  |  |  |  |  |  |  |  |  |  |  |  |  |  |  |  |  |  |  |  |
|  | >2005 | 0.00 |  |  |  | 1.00 |  |  |  | 0.00 |  |  |  | 1.00 |  |  |  | 0.00 |  |  |  | 1.00 |  |  |  |
|  | 1995-2005 | 0.30 | 0.00 | 0.11 | 0.50 | 1.40 | 0.00 | 1.15 | 1.69 | 0.26 | 0.00 | 0.12 | 0.40 | 1.32 | 0.00 | 1.16 | 1.52 | 0.31 | 0.00 | 0.16 | 0.46 | 1.39 | 0.00 | 1.20 | 1.60 |
|  | <1995 | 0.37 | 0.00 | 0.14 | 0.59 | 1.27 | 0.09 | 1.01 | 1.60 | 0.50 | 0.00 | 0.34 | 0.66 | 1.44 | 0.00 | 1.22 | 1.70 | 0.15 | 0.20 | -0.04 | 0.35 | 1.27 | 0.05 | 1.04 | 1.55 |
| Age | | 0.01 | 0.02 | 0.00 | 0.02 | 1.00 | 0.93 | 0.99 | 1.01 | 0.01 | 0.00 | 0.01 | 0.02 | 1.00 | 0.69 | 1.00 | 1.01 | 0.01 | 0.00 | 0.01 | 0.02 | 1.00 | 0.49 | 1.00 | 1.01 |
| _Cons | | -4.07 | 0.00 | -4.90 | -3.23 |  |  |  |  | -3.26 | 0.00 | -4.35 | -2.16 |  |  |  |  | -3.81 | 0.00 | -4.43 | -3.19 |  |  |  |  |
| /Ln_p | | -0.18 | 0.00 | -0.24 | -0.11 |  |  |  |  | -0.16 | 0.00 | -0.20 | -0.11 |  |  |  |  | -0.17 | 0.00 | -0.21 | -0.12 |  |  |  |  |

Incidence of all-cause and cancer specific death in categorized groups with PCLC were generally lower than that of matched single lung cancer. SLC: single lung cancer; PCLC: prostate cancer with subsequent lung cancer; Coef.: regression coefficient; SHR: risk of cancer-specific death; p: adjusted p value.

Tab S15.All cause and cancer-specific death between PCLC and matched single prostate cancer

|  |  | Coef. | p | 95% CI | | SHR | p | 95% CI | |
| --- | --- | --- | --- | --- | --- | --- | --- | --- | --- |
| Group | | |  |  |  |  |  |  |  |
|  | SPC | 0.00 |  |  |  | 1.00 |  |  |  |
|  | PCLC | 1.10 | 0.00 | 0.90 | 1.30 | 3.32 | 0.00 | 2.70 | 4.08 |
| Race | | |  |  |  |  |  |  |  |
|  | NHW | 0.00 |  |  |  | 1.00 |  |  |  |
|  | NHB | 0.14 | 0.36 | -0.07 | 0.36 | 1.09 | 0.59 | 0.88 | 1.36 |
|  | NHA | -0.30 | 0.05 | -0.56 | -0.04 | 0.86 | 0.42 | 0.66 | 1.12 |
|  | Hispanic | -0.15 | 0.37 | -0.41 | 0.12 | 0.94 | 0.72 | 0.73 | 1.23 |
| Marital | | |  |  |  |  |  |  |  |
|  | Single | 0.00 |  |  |  | 1.00 |  |  |  |
|  | Married | -0.12 | 0.36 | -0.33 | 0.09 | 1.01 | 0.96 | 0.81 | 1.25 |
|  | Unknown | 0.03 | 0.83 | -0.25 | 0.32 | 1.28 | 0.23 | 0.96 | 1.69 |
| SEER stage | | |  |  |  |  |  |  |  |
|  | Localized | 0.00 |  |  |  | 1.00 |  |  |  |
|  | Distant | 0.77 | 0.00 | 0.50 | 1.04 | 2.25 | 0.00 | 1.68 | 3.00 |
|  | Unknown | 0.13 | 0.43 | -0.14 | 0.40 | 1.18 | 0.42 | 0.90 | 1.53 |
| Surgery | | |  |  |  |  |  |  |  |
|  | No | 0.00 |  |  |  | 1.00 |  |  |  |
|  | Yes | -0.12 | 0.36 | -0.32 | 0.08 | 0.88 | 0.41 | 0.72 | 1.07 |
|  | Unknown | -0.06 | 0.78 | -0.39 | 0.28 | 0.91 | 0.68 | 0.65 | 1.26 |
| Radiation | | |  |  |  |  |  |  |  |
|  | No | 0.00 |  |  |  | 1.00 |  |  |  |
|  | Yes | 0.16 | 0.47 | -0.23 | 0.55 | 1.10 | 0.68 | 0.77 | 1.57 |
|  | Unknown | 0.35 | 0.16 | -0.04 | 0.74 | 1.22 | 0.42 | 0.85 | 1.74 |
| Category | | |  |  |  |  |  |  |  |
|  | >2005 | 0.00 |  |  |  | 1.00 |  |  |  |
|  | 1995-2005 | -0.46 | 0.00 | -0.66 | -0.26 | 0.70 | 0.00 | 0.57 | 0.86 |
|  | <1995 | -0.71 | 0.00 | -1.04 | -0.38 | 0.59 | 0.00 | 0.43 | 0.80 |
| Age | | 0.04 | 0.00 | 0.03 | 0.04 | 1.02 | 0.00 | 1.01 | 1.03 |
| _Cons | | -9.46 | 0.00 | -10.39 | -8.54 |  |  |  |  |
| /Ln_p | | 0.26 | 0.00 | 0.20 | 0.33 |  |  |  |  |

Incidence of all-cause and cancer specific death in PCLC were generally higher than that of matched single prostate cancer. SPC: single prostate cancer; PCLC: prostate cancer with subsequent lung cancer; Coef.: regression coefficient; SHR: risk of cancer-specific death; p: adjusted p value.

Tab S16.All cause and cancer-specific death between categorized group with PCLC and matched single prostate cancer

|  |  | sTPC | | | | | | | | mTPC1 | | | | | | | | mTPC2 | | | | | | | |
| --- | --- | --- | --- | --- | --- | --- | --- | --- | --- | --- | --- | --- | --- | --- | --- | --- | --- | --- | --- | --- | --- | --- | --- | --- | --- |
|  |  | Coef. | p | 95% CI | | SHR | p | 95% CI | | H.R | p | 95% CI | | SHR | p | 95% CI | | Coef. | p | 95% CI | | SHR | p | 95% CI | |
| Group | | |  |  |  |  |  |  |  |  |  |  |  |  |  |  |  |  |  |  |  |  |  |  |  |
|  | SPC | 0.00 |  |  |  | 1.00 |  |  |  | 1.00 |  |  |  | 1.00 |  |  |  | 0.00 |  |  |  | 1.00 |  |  |  |
|  | PCLC | 2.19 | 0.00 | 1.91 | 2.47 | 6.35 | 0.00 | 4.90 | 8.24 | 9.55 | 0.00 | 7.51 | 12.13 | 7.95 | 0.00 | 6.31 | 10.01 | 1.05 | 0.00 | 0.83 | 1.27 | 4.63 | 0.00 | 3.66 | 5.85 |
| Race | | |  |  |  |  |  |  |  |  |  |  |  |  |  |  |  |  |  |  |  |  |  |  |  |
|  | NHW | 0.00 |  |  |  | 1.00 |  |  |  | 1.00 |  |  |  | 1.00 |  |  |  | 0.00 |  |  |  | 1.00 |  |  |  |
|  | NHB | -0.07 | 0.74 | -0.33 | 0.19 | 0.89 | 0.51 | 0.68 | 1.15 | 1.13 | 0.42 | 0.93 | 1.36 | 1.07 | 0.88 | 0.88 | 1.29 | -0.06 | 0.67 | -0.25 | 0.14 | 0.94 | 0.65 | 0.78 | 1.13 |
|  | NHA | -0.23 | 0.34 | -0.55 | 0.09 | 0.81 | 0.44 | 0.58 | 1.11 | 0.89 | 0.67 | 0.68 | 1.18 | 0.94 | 0.90 | 0.71 | 1.23 | -0.32 | 0.05 | -0.59 | -0.05 | 0.77 | 0.13 | 0.60 | 1.01 |
|  | Hispanic |  |  |  |  |  |  |  |  | 1.09 | 0.69 | 0.85 | 1.41 | 1.12 | 0.84 | 0.87 | 1.44 | -0.28 | 0.05 | -0.55 | -0.02 | 0.81 | 0.23 | 0.62 | 1.06 |
| Marital | | |  |  |  |  |  |  |  |  |  |  |  |  |  |  |  |  |  |  |  |  |  |  |  |
|  | Single | 0.00 |  |  |  | 1.00 |  |  |  | 1.00 |  |  |  | 1.00 |  |  |  | 0.00 |  |  |  | 1.00 |  |  |  |
|  | Married | -0.20 | 0.28 | -0.43 | 0.04 | 0.83 | 0.44 | 0.64 | 1.07 | 0.97 | 0.88 | 0.81 | 1.17 | 1.02 | 0.90 | 0.85 | 1.23 | -0.03 | 0.82 | -0.22 | 0.16 | 1.09 | 0.51 | 0.91 | 1.32 |
|  | Unknown | 0.06 | 0.75 | -0.29 | 0.40 | 1.11 | 0.58 | 0.80 | 1.54 | 1.20 | 0.26 | 0.96 | 1.50 | 1.27 | 0.14 | 1.01 | 1.59 | -0.16 | 0.26 | -0.40 | 0.07 | 1.01 | 0.90 | 0.81 | 1.28 |
| SEER stage | | |  |  |  |  |  |  |  |  |  |  |  |  |  |  |  |  |  |  |  |  |  |  |  |
|  | Localized | 0.00 |  |  |  | 1.00 |  |  |  | 1.00 |  |  |  | 1.00 |  |  |  | 0.00 |  |  |  | 1.00 |  |  |  |
|  | Distant | 1.20 | 0.00 | 0.89 | 1.51 | 2.63 | 0.00 | 1.88 | 3.69 | 1.97 | 0.00 | 1.48 | 2.62 | 2.05 | 0.00 | 1.47 | 2.87 | 0.43 | 0.05 | 0.05 | 0.81 | 1.55 | 0.08 | 1.04 | 2.30 |
|  | Unknown | 0.20 | 0.60 | -0.27 | 0.68 | 0.96 | 0.86 | 0.58 | 1.57 | 1.36 | 0.11 | 1.03 | 1.82 | 1.32 | 0.17 | 0.99 | 1.76 | -0.22 | 0.26 | -0.54 | 0.10 | 0.93 | 0.73 | 0.70 | 1.23 |
| Surgery | | |  |  |  |  |  |  |  |  |  |  |  |  |  |  |  |  |  |  |  |  |  |  |  |
|  | No | 0.00 |  |  |  | 1.00 |  |  |  | 1.00 |  |  |  | 1.00 |  |  |  | 0.00 |  |  |  | 1.00 |  |  |  |
|  | Yes | -0.22 | 0.28 | -0.50 | 0.05 | 0.87 | 0.51 | 0.65 | 1.17 | 0.90 | 0.51 | 0.75 | 1.09 | 0.93 | 0.84 | 0.78 | 1.11 | -0.35 | 0.00 | -0.54 | -0.17 | 0.78 | 0.03 | 0.66 | 0.93 |
|  | Unknown | 0.36 | 0.34 | -0.16 | 0.88 | 1.20 | 0.54 | 0.73 | 1.98 | 1.05 | 0.88 | 0.68 | 1.61 | 1.06 | 0.90 | 0.69 | 1.63 | -0.27 | 0.31 | -0.69 | 0.16 | 0.77 | 0.23 | 0.55 | 1.09 |
| Radiation | | |  |  |  |  |  |  |  |  |  |  |  |  |  |  |  |  |  |  |  |  |  |  |  |
|  | No | 0.00 |  |  |  | 1.00 |  |  |  |  |  |  |  |  |  |  |  | 0.00 |  |  |  | 1.00 |  |  |  |
|  | Yes | 0.29 | 0.74 | -1.12 | 1.71 | 1.59 | 0.51 | 0.54 | 4.70 | 1.00 |  |  |  | 1.00 |  |  |  | 0.05 | 0.85 | -0.41 | 0.50 | 0.91 | 0.73 | 0.60 | 1.37 |
|  | Unknown | 0.33 | 0.74 | -1.09 | 1.74 | 1.59 | 0.51 | 0.54 | 4.68 | 1.04 | 0.84 | 0.88 | 1.23 | 1.01 | 0.90 | 0.86 | 1.19 | 0.17 | 0.59 | -0.29 | 0.62 | 1.09 | 0.73 | 0.73 | 1.63 |
| Category | | |  |  |  |  |  |  |  |  |  |  |  |  |  |  |  |  |  |  |  |  |  |  |  |
|  | >2005 | 0.00 |  |  |  | 1.00 |  |  |  | 1.00 |  |  |  | 1.00 |  |  |  | 0.00 |  |  |  | 1.00 |  |  |  |
|  | 1995-2005 | 0.15 | 0.38 | -0.10 | 0.39 | 1.32 | 0.09 | 1.04 | 1.68 | 1.21 | 0.11 | 1.01 | 1.45 | 1.30 | 0.00 | 1.09 | 1.56 | -0.54 | 0.00 | -0.76 | -0.32 | 0.66 | 0.00 | 0.53 | 0.83 |
|  | <1995 | 0.14 | 0.74 | -0.41 | 0.68 | 1.44 | 0.44 | 0.81 | 2.58 | 1.03 | 0.88 | 0.72 | 1.47 | 1.07 | 0.90 | 0.75 | 1.52 | -0.80 | 0.00 | -1.21 | -0.39 | 0.59 | 0.03 | 0.41 | 0.86 |
| Age | | 0.03 | 0.00 | 0.02 | 0.05 | 1.01 | 0.44 | 1.00 | 1.03 | 1.01 | 0.05 | 1.00 | 1.02 | 1.00 | 0.90 | 0.99 | 1.01 | 0.04 | 0.00 | 0.03 | 0.05 | 1.03 | 0.00 | 1.02 | 1.04 |
| _Cons | | -8.16 | 0.00 | -9.93 | -6.40 |  |  |  |  |  |  |  |  |  |  |  |  | -16.56 | 0.00 | -17.79 | -15.32 |  |  |  |  |
| /Ln_p | | -0.11 | 0.01 | -0.19 | -0.03 |  |  |  |  |  |  |  |  |  |  |  |  | 0.96 | 0.00 | 0.90 | 1.03 |  |  |  |  |
| phtest | |  |  |  |  |  |  |  |  |  | 0.11 |  |  |  |  |  |  |  |  |  |  |  |  |  |  |

Incidence of all-cause and cancer specific death in the categorized groups were generally higher than that of matched single prostate cancer. SPC: single prostate cancer; PCLC: prostate cancer with subsequent lung cancer; H.R: risk of all-cause death; Coef.: regression coefficient; SHR: risk of cancer-specific death; p: adjusted p value.

Tab S17. Median overall-survival since lung primary among patients with LCBC, BCLC, LCPC and PCLC

|  | LCBC | | | | BCLC | | | | LCPC | | | | PCLC | | | |
| --- | --- | --- | --- | --- | --- | --- | --- | --- | --- | --- | --- | --- | --- | --- | --- | --- |
|  | No. | 50% | 95% CI | | No. | 50% | 95% CI | | NO. | 50% | 95% CI | | NO. | 50% | 95% CI | |
| sTPC | 117 | 16 | 10 | 21 | 163 | 18 | 12 | 29 | 306 | 13 | 11 | 15 | 632 | 16 | 14 | 19 |
| mTPC1 | 143 | 92 | 65 | 133 | 569 | 12 | 10 | 13 | 538 | 129 | 113 | 153 | 2638 | 12 | 11 | 13 |
| mTPC2 | 82 | 345 | 284 | - | 627 | 10 | 9 | 12 | 269 | 319 | 250 | - | 3326 | 11 | 11 | 12 |
| total | 342 | 103 | 70 | 158 | 1359 | 11 | 10 | 13 | 1113 | 137 | 122 | 154 | 6596 | 12 | 11 | 12 |

LCBC: lung cancer with subsequent bladder cancer; BCLC: lung cancer with previous bladder cancer; LCPC: lung cancer with subsequent prostate cancer; PCLC: lung cancer with previous prostate cancer.

Tab S18. Covariates associated with all-cause and cancer-specific death since lung primary in LCBC and BCLC

|  |  | LCBC | | | | | | | | BCLC | | | | | | | |
| --- | --- | --- | --- | --- | --- | --- | --- | --- | --- | --- | --- | --- | --- | --- | --- | --- | --- |
|  |  | Coef. | p | 95% CI | | SHR | p | 95% CI | | Coef. | p | 95% CI | | SHR | p | 95% CI | |
| Group | | |  |  |  |  |  |  |  |  |  |  |  |  |  |  |  |
|  | sTPC | 0.00 |  |  |  | 1.00 |  |  |  | 0.00 |  |  |  | 1.00 |  |  |  |
|  | mTPC1 | -1.68 | 0.00 | -2.07 | -1.29 | 0.25 | 0.00 | 0.17 | 0.37 | 0.09 | 0.78 | -0.12 | 0.31 | 1.10 | 0.63 | 0.90 | 1.35 |
|  | mTPC2 | -3.04 | 0.00 | -3.67 | -2.41 | 0.12 | 0.00 | 0.06 | 0.21 | 0.16 | 0.46 | -0.05 | 0.38 | 1.15 | 0.44 | 0.95 | 1.41 |
| Year of lung diagnosis | | | |  |  |  |  |  |  |  |  |  |  |  |  |  |  |
|  | >2005 | 0.00 |  |  |  | 1.00 |  |  |  | 0.00 |  |  |  | 1.00 |  |  |  |
|  | 1995-2005 | -0.29 | 0.54 | -1.04 | 0.47 | 0.85 | 0.78 | 0.39 | 1.85 | -0.07 | 0.78 | -0.36 | 0.21 | 1.03 | 0.90 | 0.76 | 1.40 |
|  | <1995 | -0.01 | 0.99 | -0.86 | 0.84 | 1.29 | 0.67 | 0.58 | 2.84 | 0.15 | 0.78 | -0.17 | 0.47 | 1.27 | 0.44 | 0.91 | 1.77 |
| Marital of first diagnosis | | | |  |  |  |  |  |  |  |  |  |  |  |  |  |  |
|  | Single | 0.00 |  |  |  | 1.00 |  |  |  | 0.00 |  |  |  | 1.00 |  |  |  |
|  | Married | 0.40 | 0.31 | -0.16 | 0.96 | 1.26 | 0.51 | 0.76 | 2.09 | -0.06 | 0.78 | -0.28 | 0.15 | 0.93 | 0.72 | 0.74 | 1.16 |
|  | Unknown | 1.23 | 0.11 | 0.14 | 2.31 | 2.96 | 0.00 | 1.56 | 5.62 | 0.27 | 0.46 | -0.11 | 0.64 | 1.08 | 0.86 | 0.73 | 1.59 |
| SEER stage of first diagnosis | | | | |  |  |  |  |  |  |  |  |  |  |  |  |  |
|  | Localized | 0.00 |  |  |  | 1.00 |  |  |  | 0.00 |  |  |  | 1.00 |  |  |  |
|  | Regional | 0.28 | 0.44 | -0.20 | 0.75 | 1.28 | 0.50 | 0.78 | 2.09 | 0.00 | 1.00 | -0.25 | 0.25 | 1.12 | 0.63 | 0.89 | 1.42 |
|  | Distant | 0.49 | 0.15 | -0.04 | 1.02 | 1.78 | 0.07 | 1.04 | 3.03 | -0.29 | 0.81 | -1.90 | 1.31 | 1.33 | 0.72 | 0.54 | 3.30 |
|  | Unknown | 0.57 | 0.15 | -0.04 | 1.19 | 1.94 | 0.05 | 1.10 | 3.42 | -0.13 | 0.81 | -0.73 | 0.47 | 0.89 | 0.86 | 0.48 | 1.66 |
| Surgery of first diagnosis | | | | |  |  |  |  |  |  |  |  |  |  |  |  |  |
|  | No | 0.00 |  |  |  | 1.00 |  |  |  | 0.00 |  |  |  | 1.00 |  |  |  |
|  | Yes | -1.34 | 0.00 | -1.79 | -0.88 | 0.47 | 0.00 | 0.30 | 0.73 | -0.23 | 0.49 | -0.58 | 0.12 | 0.89 | 0.72 | 0.63 | 1.28 |
|  | Unknown | -0.01 | 0.99 | -1.06 | 1.05 | 1.45 | 0.55 | 0.60 | 3.51 | -0.57 | 0.78 | -2.62 | 1.49 | 0.71 | 0.88 | 0.07 | 7.36 |
| Radiation of first diagnosis | | | | |  |  |  |  |  |  |  |  |  |  |  |  |  |
|  | No | 0.00 |  |  |  | 1.00 |  |  |  |  |  |  |  |  |  |  |  |
|  | Yes | -0.62 | 0.53 | -2.14 | 0.90 | 0.27 | 0.03 | 0.10 | 0.72 | 0.00 |  |  |  | 1.00 |  |  |  |
|  | Unknown | -0.72 | 0.50 | -2.26 | 0.81 | 0.23 | 0.03 | 0.08 | 0.64 | 0.16 | 0.78 | -0.38 | 0.70 | 1.03 | 0.90 | 0.66 | 1.60 |
| Age of lung primary | | | |  |  |  |  |  |  |  |  |  |  |  |  |  |  |
|  | <55 | 0.00 |  |  |  | 1.00 |  |  |  | 0.00 |  |  |  | 1.00 |  |  |  |
|  | 55-75 | -0.54 | 0.44 | -1.49 | 0.41 | 0.54 | 0.33 | 0.22 | 1.35 | -0.06 | 0.87 | -0.55 | 0.42 | 0.91 | 0.72 | 0.66 | 1.25 |
|  | >75 | -0.47 | 0.50 | -1.45 | 0.51 | 0.45 | 0.18 | 0.18 | 1.16 | 0.17 | 0.78 | -0.32 | 0.66 | 0.98 | 0.90 | 0.70 | 1.37 |
| Insurance of secondary diagnosis | | | | | |  |  |  |  |  |  |  |  |  |  |  |  |
|  | No | 0.00 |  |  |  | 1.00 |  |  |  | 0.00 |  |  |  | 1.00 |  |  |  |
|  | Yes | 0.35 | 0.84 | -2.00 | 2.71 | 0.66 | 0.71 | 0.14 | 2.99 | 0.14 | 0.88 | -1.29 | 1.56 | 1.91 | 0.55 | 0.65 | 5.61 |
|  | Unknown | 0.78 | 0.60 | -1.68 | 3.24 | 0.77 | 0.80 | 0.15 | 4.07 | 0.54 | 0.78 | -0.91 | 1.98 | 2.59 | 0.36 | 0.85 | 7.91 |
| Marital of secondary diagnosis | | | | | |  |  |  |  |  |  |  |  |  |  |  |  |
|  | Single | 0.00 |  |  |  | 1.00 |  |  |  | 0.00 |  |  |  | 1.00 |  |  |  |
|  | Married | -0.99 | 0.00 | -1.54 | -0.43 | 0.38 | 0.00 | 0.23 | 0.62 | 0.04 | 0.81 | -0.16 | 0.24 | 1.09 | 0.72 | 0.88 | 1.35 |
|  | Unknown | -0.96 | 0.13 | -1.87 | -0.04 | 0.48 | 0.15 | 0.22 | 1.07 | -0.16 | 0.78 | -0.54 | 0.23 | 1.17 | 0.72 | 0.77 | 1.78 |
| SEER stage of secondary diagnosis | | | | | | |  |  |  |  |  |  |  |  |  |  |  |
|  | Localized | 0.00 |  |  |  | 1.00 |  |  |  | 0.00 |  |  |  | 1.00 |  |  |  |
|  | Regional | 0.90 | 0.11 | 0.07 | 1.73 | 1.00 | 1.00 | 0.30 | 3.31 | 0.51 | 0.00 | 0.28 | 0.73 | 1.55 | 0.00 | 1.26 | 1.91 |
|  | Distant | 1.98 | 0.10 | 0.34 | 3.63 | 3.51 | 0.18 | 0.82 | 14.96 | 1.36 | 0.00 | 1.14 | 1.57 | 3.32 | 0.00 | 2.72 | 4.04 |
|  | Unknown | -1.39 | 0.15 | -2.81 | 0.04 | 0.22 | 0.05 | 0.06 | 0.77 | 0.40 | 0.05 | 0.11 | 0.68 | 1.62 | 0.00 | 1.23 | 2.13 |
| Surgery of secondary diagnosis | | | | |  |  |  |  |  |  |  |  |  |  |  |  |  |
|  | No | 0.00 |  |  |  | 1.00 |  |  |  | 0.00 |  |  |  | 1.00 |  |  |  |
|  | Yes | -0.27 | 0.53 | -0.90 | 0.36 | 0.73 | 0.50 | 0.40 | 1.36 | -1.10 | 0.00 | -1.31 | -0.90 | 0.52 | 0.00 | 0.44 | 0.62 |
|  | Unknown |  |  |  |  |  |  |  |  | 0.20 | 0.78 | -0.53 | 0.93 | 0.59 | 0.48 | 0.27 | 1.30 |
| Radiation of secondary diagnosis | | | | |  |  |  |  |  |  |  |  |  |  |  |  |  |
|  | No |  |  |  |  |  |  |  |  | 0.00 |  |  |  | 1.00 |  |  |  |
|  | Yes | 0.00 |  |  |  | 1.00 |  |  |  | -0.51 | 0.45 | -1.14 | 0.13 | 0.56 | 0.17 | 0.32 | 0.99 |
|  | Unknown | 0.59 | 0.44 | -0.48 | 1.66 | 0.85 | 0.80 | 0.29 | 2.52 | -0.54 | 0.39 | -1.17 | 0.09 | 0.55 | 0.17 | 0.32 | 0.96 |
| _Cons | | -3.33 | 0.14 | -6.62 | -0.04 |  |  |  |  | -3.24 | 0.00 | -4.93 | -1.55 |  |  |  |  |
| /Ln_p | | 0.24 |  | 0.12 | 0.35 |  |  |  |  | -0.06 |  | -0.11 | -0.01 |  |  |  |  |

The regression analysis suggested incidence of all-cause and cancer-specific death in group sTPC with LCBC were statically higher than that in group mTPC1 and mTPC2; for BCLC, incidence of all-cause and cancer-specific death in group sTPC with were not statically different from that in group mTPC1 and mTPC2. LCBC: lung cancer with subsequent bladder cancer; BCLC: lung cancer with previous bladder cancer; Coef.: regression coefficient; SHR: risk of cancer-specific death.

Tab S19. Covariates associated with all-cause and cancer-specific death since lung primary in LCPC and PCLC

|  |  | LCPC | | | | | | | | PCLC | | | | | | | |
| --- | --- | --- | --- | --- | --- | --- | --- | --- | --- | --- | --- | --- | --- | --- | --- | --- | --- |
|  |  | Coef. | p | 95% CI | | SHR | p | 95% CI | | Coef. | p | 95% CI | | SHR | p | 95% CI | |
| Group | | | | | |  |  |  |  |  |  |  |  |  |  |  |  |
|  | sTPC | 0.00 |  |  |  | 1.00 |  |  |  | 0.00 |  |  |  | 1.00 |  |  |  |
|  | mTPC1 | -1.40 | 0.00 | -1.61 | -1.19 | 0.35 | 0.00 | 0.27 | 0.45 | 0.17 | 0.00 | 0.06 | 0.27 | 1.09 | 0.25 | 0.98 | 1.22 |
|  | mTPC2 | -2.50 | 0.00 | -2.84 | -2.17 | 0.18 | 0.00 | 0.13 | 0.25 | 0.21 | 0.00 | 0.10 | 0.32 | 1.08 | 0.38 | 0.96 | 1.21 |
| Year of lung diagnosis | | | | | |  |  |  |  |  |  |  |  |  |  |  |  |
|  | >2005 | 0.00 |  |  |  | 1.00 |  |  |  | 0.00 |  |  |  | 1.00 |  |  |  |
|  | 1995-2005 | -0.05 | 0.75 | -0.36 | 0.25 | 0.96 | 0.94 | 0.57 | 1.62 | 0.00 | 0.99 | -0.13 | 0.13 | 1.03 | 0.87 | 0.90 | 1.18 |
|  | <1995 | 0.43 | 0.07 | 0.07 | 0.78 | 1.29 | 0.62 | 0.74 | 2.25 | 0.27 | 0.00 | 0.10 | 0.44 | 1.18 | 0.19 | 0.99 | 1.41 |
| Marital of first diagnosis | | | | | |  |  |  |  |  |  |  |  |  |  |  |  |
|  | Single | 0.00 |  |  |  | 1.00 |  |  |  | 0.00 |  |  |  | 1.00 |  |  |  |
|  | Married | -0.06 | 0.75 | -0.39 | 0.26 | 1.57 | 0.86 | 0.22 | 11.39 | 0.06 | 0.23 | -0.03 | 0.16 | 0.95 | 0.91 | 0.51 | 1.77 |
|  | Unknown | -0.23 | 0.66 | -0.87 | 0.41 | 1.80 | 0.85 | 0.23 | 13.95 | 0.10 | 0.17 | -0.02 | 0.23 | 1.17 | 0.87 | 0.62 | 2.18 |
| SEER stage of first diagnosis | | | | | |  |  |  |  |  |  |  |  |  |  |  |  |
|  | Localized | 0.00 |  |  |  | 1.00 |  |  |  | 0.00 |  |  |  | 1.00 |  |  |  |
|  | Regional | 0.27 | 0.14 | -0.01 | 0.54 | 1.12 | 0.69 | 0.84 | 1.49 |  |  |  |  |  |  |  |  |
|  | Distant | 0.66 | 0.00 | 0.35 | 0.97 | 1.77 | 0.00 | 1.23 | 2.54 | 0.42 | 0.00 | 0.25 | 0.59 | 1.45 | 0.00 | 1.23 | 1.71 |
|  | Unknown | 0.22 | 0.30 | -0.08 | 0.53 | 1.19 | 0.52 | 0.87 | 1.64 | 0.11 | 0.09 | 0.00 | 0.22 | 1.06 | 0.50 | 0.95 | 1.19 |
| Surgery of first diagnosis | | | | | | |  |  |  |  |  |  |  |  |  |  |  |
|  | No | 0.00 |  |  |  | 1.00 |  |  |  | 0.00 |  |  |  | 1.00 |  |  |  |
|  | Yes | -0.90 | 0.00 | -1.15 | -0.64 | 0.51 | 0.00 | 0.38 | 0.68 | -0.05 | 0.23 | -0.12 | 0.02 | 0.99 | 0.91 | 0.92 | 1.07 |
|  | Unknown | -0.14 | 0.75 | -0.98 | 0.70 | 0.24 | 0.13 | 0.06 | 0.95 | 0.13 | 0.40 | -0.13 | 0.40 | 1.05 | 0.91 | 0.80 | 1.37 |
| Radiation of first diagnosis | | | | | | |  |  |  |  |  |  |  |  |  |  |  |
|  | No | 0.00 |  |  |  | 1.00 |  |  |  | 0.00 |  |  |  | 1.00 |  |  |  |
|  | Yes | -1.84 | 0.08 | -3.51 | -0.16 | 0.17 | 0.00 | 0.09 | 0.31 | -0.06 | 0.75 | -0.36 | 0.25 | 0.98 | 0.91 | 0.73 | 1.31 |
|  | Unknown | -2.17 | 0.04 | -3.84 | -0.50 | 0.13 | 0.00 | 0.07 | 0.23 | -0.12 | 0.52 | -0.43 | 0.19 | 0.92 | 0.85 | 0.69 | 1.24 |
| Age of lung primary | | | | | | |  |  |  |  |  |  |  |  |  |  |  |
|  | <55 | 0.00 |  |  |  | 1.00 |  |  |  | 0.00 |  |  |  | 1.00 |  |  |  |
|  | 55-75 | 0.11 | 0.71 | -0.26 | 0.47 | 0.98 | 0.94 | 0.71 | 1.34 | 0.12 | 0.58 | -0.24 | 0.48 | 1.09 | 0.85 | 0.82 | 1.45 |
|  | >75 | 0.24 | 0.48 | -0.18 | 0.66 | 1.03 | 0.94 | 0.69 | 1.53 | 0.32 | 0.13 | -0.04 | 0.68 | 1.19 | 0.43 | 0.89 | 1.59 |
| Insurance of secondary diagnosis | | | | | | |  |  |  |  |  |  |  |  |  |  |  |
|  | No | 0.00 |  |  |  | 1.00 |  |  |  | 0.00 |  |  |  | 1.00 |  |  |  |
|  | Yes | -0.65 | 0.58 | -2.07 | 0.76 | 0.51 | 0.52 | 0.16 | 1.65 | -0.77 | 0.00 | -1.17 | -0.36 | 0.46 | 0.00 | 0.30 | 0.69 |
|  | Unknown | -0.31 | 0.75 | -1.73 | 1.11 | 0.74 | 0.85 | 0.24 | 2.33 | -0.58 | 0.02 | -1.00 | -0.16 | 0.52 | 0.00 | 0.34 | 0.80 |
| Marital of secondary diagnosis | | | | | | |  |  |  |  |  |  |  |  |  |  |  |
|  | Single | 0.00 |  |  |  | 1.00 |  |  |  | 0.00 |  |  |  | 1.00 |  |  |  |
|  | Married | 0.06 | 0.75 | -0.27 | 0.39 | 0.97 | 0.94 | 0.76 | 1.24 | -0.13 | 0.00 | -0.22 | -0.05 | 0.95 | 0.25 | 0.89 | 1.01 |
|  | Unknown | -0.12 | 0.71 | -0.51 | 0.27 | 0.84 | 0.62 | 0.57 | 1.23 | -0.12 | 0.23 | -0.29 | 0.05 | 1.01 | 0.91 | 0.86 | 1.19 |
| SEER stage of secondary diagnosis | | | | | | |  |  |  |  |  |  |  |  |  |  |  |
|  | Localized | 0.00 |  |  |  | 1.00 |  |  |  | 0.00 |  |  |  | 1.00 |  |  |  |
|  | Regional |  |  |  |  |  |  |  |  | 0.62 | 0.00 | 0.52 | 0.73 | 1.74 | 0.00 | 1.58 | 1.91 |
|  | Distant | 0.34 | 0.08 | 0.04 | 0.64 | 1.00 | 0.99 | 0.64 | 1.57 | 1.30 | 0.00 | 1.19 | 1.40 | 3.09 | 0.00 | 2.81 | 3.39 |
|  | Unknown | 0.36 | 0.04 | 0.11 | 0.62 | 1.23 | 0.35 | 0.94 | 1.61 | 0.68 | 0.00 | 0.54 | 0.83 | 1.74 | 0.00 | 1.52 | 2.00 |
| Surgery of secondary diagnosis | | | | | | |  |  |  |  |  |  |  |  |  |  |  |
|  | No | 0.00 |  |  |  | 1.00 |  |  |  | 0.00 |  |  |  | 1.00 |  |  |  |
|  | Yes | -0.25 | 0.07 | -0.47 | -0.04 | 0.98 | 0.94 | 0.78 | 1.24 | -1.03 | 0.00 | -1.12 | -0.94 | 0.49 | 0.00 | 0.45 | 0.53 |
|  | Unknown | 0.28 | 0.57 | -0.28 | 0.83 | 1.57 | 0.52 | 0.70 | 3.51 | -0.09 | 0.63 | -0.39 | 0.22 | 0.98 | 0.91 | 0.72 | 1.33 |
| Radiation of secondary diagnosis | | | | | | |  |  |  |  |  |  |  |  |  |  |  |
|  | No | 0.00 |  |  |  | 1.00 |  |  |  | 0.00 |  |  |  | 1.00 |  |  |  |
|  | Yes | -1.05 | 0.30 | -2.42 | 0.33 | 0.35 | 0.11 | 0.13 | 0.92 | -0.54 | 0.00 | -0.84 | -0.24 | 0.77 | 0.35 | 0.53 | 1.12 |
|  | Unknown | -0.59 | 0.58 | -1.96 | 0.78 | 0.47 | 0.33 | 0.18 | 1.22 | -0.58 | 0.00 | -0.88 | -0.28 | 0.73 | 0.25 | 0.51 | 1.06 |
| _Cons | | -0.95 | 0.58 | -3.05 | 1.15 |  |  |  |  | -2.39 | 0.00 | -3.09 | -1.68 |  |  |  |  |
| /Ln_p | | 0.11 |  | 0.04 | 0.17 |  |  |  |  | -0.12 |  | -0.15 | -0.10 |  |  |  |  |

The regression analysis suggested incidence of all-cause and cancer-specific death in group sTPC with LCPC were statically higher than that in group mTPC1 and mTPC2; for PCLC, incidence of all-cause death in group sTPC was lower than that in group mTPC1 and mTPC2, whereas incidence of cancer-specific death in group sTPC was not statically different from that in group mTPC1 and mTPC2. LCPC: lung cancer with subsequent prostate cancer; PCLC: lung cancer with previous prostate cancer; H.R: risk of all-cause death; SHR: risk of cancer-specific death. Coef.: regression coefficient; SHR: risk of cancer-specific death

Tab S20. balance test between LABP and single lung cancer in the matched cohort

|  | Mean | |  |  |
| --- | --- | --- | --- | --- |
| Variable | Treated | Control | bias | p |
| age | 69.39 | 69.56 | 1.70 | 0.81 |
| 1.surgery | 0.58 | 0.57 | 1.90 | 0.82 |
| 2.surgery | 0.01 | 0.01 | 0.00 | 1.00 |
| 2.seerstage | 0.28 | 0.28 | 0.60 | 0.93 |
| 3.seerstage | 0.21 | 0.20 | 1.00 | 0.92 |
| 4.seerstage | 0.15 | 0.15 | 0.80 | 0.92 |
| 2.race | 0.04 | 0.04 | 0.00 | 1.00 |
| 3.race | 0.02 | 0.02 | 0.00 | 1.00 |
| 4.race | 0.02 | 0.01 | 4.50 | 0.40 |
| 1.radiation | 0.38 | 0.37 | 3.00 | 0.69 |
| 2.radiation | 0.61 | 0.63 | 3.00 | 0.69 |
| 1.marital | 0.74 | 0.73 | 1.90 | 0.79 |
| 2.marital | 0.02 | 0.02 | 1.80 | 0.78 |
| 2.category | 0.34 | 0.34 | 0.60 | 0.94 |
| 3.category | 0.23 | 0.24 | 1.40 | 0.86 |
| 2.seerstage#1.surgery | 0.19 | 0.19 | 1.00 | 0.92 |
| 2.seerstage#2.surgery | 0.00 | 0.00 | 0.00 | 1.00 |
| 3.seerstage#1.surgery | 0.03 | 0.01 | 11.90 | 0.19 |
| 4.seerstage#1.surgery | 0.08 | 0.09 | 1.20 | 0.89 |
| 4.seerstage#2.surgery | 0.01 | 0.01 | 0.00 | 1.00 |
| 2.category#2.seerstage | 0.10 | 0.11 | 1.70 | 0.80 |
| 2.category#3.seerstage | 0.08 | 0.07 | 1.50 | 0.88 |
| 3.category#2.seerstage | 0.04 | 0.04 | 2.80 | 0.70 |
| 3.category#3.seerstage | 0.01 | 0.01 | 3.10 | 0.71 |
| 3.category#4.seerstage | 0.13 | 0.13 | 0.90 | 0.91 |
| 1.radiation#1.surgery | 0.11 | 0.09 | 4.30 | 0.61 |
| 1.radiation#2.surgery | 0.01 | 0.01 | 0.00 | 1.00 |
| 2.radiation#1.surgery | 0.47 | 0.48 | 0.70 | 0.94 |
| 2.radiation#2.surgery | 0.01 | 0.01 | 0.00 | 1.00 |
| 2.seerstage#c.age | 19.17 | 19.27 | 0.30 | 0.97 |
| 3.seerstage#c.age | 14.30 | 14.34 | 0.20 | 0.98 |
| 4.seerstage#c.age | 10.04 | 10.22 | 0.70 | 0.92 |
| 2.race#2.seerstage | 0.01 | 0.01 | 0.00 | 1.00 |
| 2.race#3.seerstage | 0.02 | 0.02 | 0.00 | 1.00 |
| 2.race#4.seerstage | 0.01 | 0.01 | 2.60 | 0.65 |
| 3.race#2.seerstage | 0.01 | 0.01 | 0.00 | 1.00 |
| 4.race#2.seerstage | 0.01 | 0.00 | 2.10 | 0.56 |
| 4.race#3.seerstage | 0.00 | 0.00 | 6.00 | 0.32 |
| 4.race#4.seerstage | 0.00 | 0.00 | 0.00 | 1.00 |
| c.age#c.age | 4897.10 | 4920.10 | 1.70 | 0.81 |
| 1.surgery#c.age | 39.49 | 38.89 | 1.90 | 0.82 |
| 2.surgery#c.age | 0.78 | 0.78 | 0.00 | 1.00 |
| 1.marital#2.race | 0.04 | 0.04 | 1.40 | 0.84 |
| 1.marital#3.race | 0.01 | 0.01 | 1.60 | 0.74 |
| 1.marital#4.race | 0.01 | 0.01 | 3.80 | 0.48 |
| 2.marital#2.race | 0.00 | 0.00 | 0.00 | 1.00 |
| 2.marital#4.race | 0.00 | 0.00 | 5.80 | 0.32 |
| 2.category#1.surgery | 0.23 | 0.23 | 0.80 | 0.93 |
| 2.category#2.surgery | 0.00 | 0.00 | 0.00 | 1.00 |
| 3.category#1.surgery | 0.16 | 0.16 | 1.00 | 0.92 |
| 3.category#2.surgery | 0.01 | 0.01 | 0.00 | 1.00 |
| 2.category#2.race | 0.01 | 0.01 | 1.90 | 0.74 |
| 2.category#3.race | 0.01 | 0.01 | 0.00 | 1.00 |
| 2.category#4.race | 0.01 | 0.01 | 0.00 | 1.00 |
| 3.category#2.race | 0.01 | 0.01 | 0.00 | 1.00 |
| 3.category#3.race | 0.01 | 0.01 | 0.00 | 1.00 |
| 3.category#4.race | 0.01 | 0.00 | 3.80 | 0.56 |
| 2.category#c.age | 23.53 | 23.48 | 0.10 | 0.99 |
| 3.category#c.age | 15.32 | 15.66 | 1.30 | 0.87 |
| 2.category#1.marital | 0.26 | 0.25 | 2.10 | 0.79 |
| 2.category#2.marital | 0.01 | 0.01 | 0.00 | 1.00 |
| 3.category#1.marital | 0.17 | 0.17 | 0.00 | 1.00 |
| 3.category#2.marital | 0.01 | 0.00 | 3.90 | 0.56 |
| 2.race#c.age | 2.93 | 3.00 | 0.40 | 0.95 |
| 3.race#c.age | 1.13 | 1.10 | 0.20 | 0.97 |
| 4.race#c.age | 1.56 | 1.01 | 4.20 | 0.45 |
| 1.marital#2.seerstage | 0.20 | 0.20 | 0.00 | 1.00 |
| 1.marital#3.seerstage | 0.15 | 0.14 | 2.20 | 0.83 |
| 1.marital#4.seerstage | 0.12 | 0.12 | 0.90 | 0.91 |
| 2.marital#2.seerstage | 0.01 | 0.00 | 2.80 | 0.56 |
| 2.marital#3.seerstage | 0.00 | 0.01 | 6.00 | 0.56 |
| 2.marital#4.seerstage | 0.00 | 0.00 | 0.00 | 1.00 |

LABP: lung cancer with subsequent bladder cancer; bias: absolutely standardized difference; p: t test between matched treated and control group.

Tab S21. balance test between group sTPC with LABP and single lung cancer in the matched cohort

|  | Mean | |  |  |
| --- | --- | --- | --- | --- |
| Variable | Treated | Control | %bias | p |
| age | 70.91 | 70.50 | 4.20 | 0.70 |
| 2.seerstage | 0.27 | 0.28 | 1.80 | 0.89 |
| 3.seerstage | 0.40 | 0.40 | 0.00 | 1.00 |
| 4.seerstage | 0.18 | 0.18 | 0.00 | 1.00 |
| 2.race | 0.03 | 0.03 | 0.00 | 1.00 |
| 3.race | 0.01 | 0.01 | 0.00 | 1.00 |
| 1.surgery | 0.34 | 0.33 | 1.90 | 0.89 |
| 2.surgery | 0.03 | 0.03 | 0.00 | 1.00 |
| 1.radiation | 0.48 | 0.49 | 1.70 | 0.90 |
| 2.radiation | 0.50 | 0.50 | 1.70 | 0.90 |
| 2.category | 0.27 | 0.26 | 3.70 | 0.77 |
| 3.category | 0.19 | 0.21 | 4.20 | 0.74 |
| 1.marital | 0.74 | 0.74 | 0.00 | 1.00 |
| 1.surgery#2.seerstage | 0.15 | 0.15 | 0.00 | 1.00 |
| 1.surgery#3.seerstage | 0.05 | 0.04 | 5.40 | 0.76 |
| 1.surgery#4.seerstage | 0.06 | 0.06 | 0.00 | 1.00 |
| 2.surgery#2.seerstage | 0.01 | 0.01 | 0.00 | 1.00 |
| 2.surgery#4.seerstage | 0.02 | 0.02 | 0.00 | 1.00 |
| c.age#c.age | 5087.10 | 5033.60 | 4.20 | 0.71 |
| 2.seerstage#c.age | 19.24 | 19.90 | 2.00 | 0.88 |
| 3.seerstage#c.age | 27.89 | 27.50 | 1.50 | 0.93 |
| 4.seerstage#c.age | 12.86 | 12.86 | 0.00 | 1.00 |
| 1.marital#2.seerstage | 0.17 | 0.18 | 2.00 | 0.86 |
| 1.marital#3.seerstage | 0.30 | 0.31 | 2.50 | 0.89 |
| 1.marital#4.seerstage | 0.15 | 0.15 | 0.00 | 1.00 |
| 1.marital#2.category | 0.21 | 0.19 | 4.20 | 0.74 |
| 1.marital#3.category | 0.12 | 0.12 | 0.00 | 1.00 |
| 2.category#1.surgery | 0.09 | 0.09 | 3.00 | 0.82 |
| 2.category#2.surgery | 0.01 | 0.01 | 0.00 | 1.00 |
| 3.category#1.surgery | 0.07 | 0.07 | 0.00 | 1.00 |
| 3.category#2.surgery | 0.02 | 0.02 | 0.00 | 1.00 |
| 2.category#2.race | 0.02 | 0.02 | 0.00 | 1.00 |
| 2.category#3.race | 0.01 | 0.01 | 0.00 | 1.00 |
| 2.race#2.seerstage | 0.01 | 0.01 | 0.00 | 1.00 |
| 2.race#3.seerstage | 0.02 | 0.02 | 0.00 | 1.00 |
| 2.race#4.seerstage | 0.01 | 0.01 | 0.00 | 1.00 |
| 3.race#2.seerstage | 0.01 | 0.01 | 0.00 | 1.00 |

LABP: lung cancer with subsequent bladder cancer; bias: absolutely standardized difference; p: t test between matched treated and control group.

Tab S22. balance test between group mTPC1 with LABP and single lung cancer in the matched cohort

|  | Mean | |  |  |
| --- | --- | --- | --- | --- |
| Variable | Treated | Control | %bias | P |
| Age | 70.44 | 70.94 | 5.10 | 0.65 |
| 2.seerstage | 0.32 | 0.33 | 1.50 | 0.90 |
| 3.seerstage | 0.15 | 0.14 | 5.30 | 0.73 |
| 4.seerstage | 0.11 | 0.11 | 0.00 | 1.00 |
| 1.surgery | 0.57 | 0.57 | 0.00 | 1.00 |
| 2.surgery | 0.01 | 0.01 | 0.00 | 1.00 |
| 2.race | 0.08 | 0.07 | 2.40 | 0.82 |
| 3.race | 0.01 | 0.01 | 0.00 | 1.00 |
| 4.race | 0.04 | 0.04 | 0.00 | 1.00 |
| 1.radiation | 0.41 | 0.40 | 1.40 | 0.90 |
| 2.radiation | 0.59 | 0.60 | 1.40 | 0.90 |
| 1.insurance | 0.44 | 0.41 | 5.70 | 0.63 |
| 2.insurance | 0.56 | 0.59 | 5.70 | 0.63 |
| 2.category | 0.33 | 0.36 | 6.10 | 0.62 |
| 3.category | 0.20 | 0.21 | 1.80 | 0.88 |
| 2.category#2.seerstage | 0.11 | 0.14 | 6.70 | 0.59 |
| 2.category#3.seerstage | 0.05 | 0.05 | 0.00 | 1.00 |
| 3.category#2.seerstage | 0.04 | 0.04 | 3.90 | 0.76 |
| 3.category#4.seerstage | 0.11 | 0.11 | 0.00 | 1.00 |
| 1.surgery#2.seerstage | 0.22 | 0.23 | 2.20 | 0.89 |
| 1.surgery#3.seerstage | 0.01 | 0.00 | 15.70 | 0.16 |
| 1.surgery#4.seerstage | 0.04 | 0.04 | 0.00 | 1.00 |
| 2.surgery#4.seerstage | 0.01 | 0.01 | 0.00 | 1.00 |
| 1.radiation#1.surgery | 0.11 | 0.11 | 0.00 | 1.00 |
| 1.radiation#2.surgery | 0.01 | 0.01 | 0.00 | 1.00 |
| 2.radiation#1.surgery | 0.46 | 0.46 | 0.00 | 1.00 |
| 2.race#2.seerstage | 0.01 | 0.00 | 8.00 | 0.16 |
| 2.race#3.seerstage | 0.03 | 0.04 | 5.80 | 0.74 |
| 2.race#4.seerstage | 0.01 | 0.01 | 0.00 | 1.00 |
| 4.race#2.seerstage | 0.01 | 0.02 | 5.00 | 0.65 |
| 4.race#3.seerstage | 0.01 | 0.00 | 10.70 | 0.32 |
| 1.radiation#2.seerstage | 0.14 | 0.14 | 1.90 | 0.86 |
| 1.radiation#3.seerstage | 0.09 | 0.07 | 7.00 | 0.66 |
| 1.radiation#4.seerstage | 0.07 | 0.07 | 0.00 | 1.00 |
| 2.radiation#2.seerstage | 0.19 | 0.19 | 0.00 | 1.00 |
| 2.radiation#3.seerstage | 0.06 | 0.06 | 0.00 | 1.00 |
| 2.radiation#4.seerstage | 0.04 | 0.04 | 0.00 | 1.00 |
| 2.race#c.age | 5.24 | 4.72 | 2.70 | 0.81 |
| 3.race#c.age | 0.84 | 0.84 | 0.00 | 1.00 |
| 4.race#c.age | 2.90 | 2.96 | 0.40 | 0.97 |
| 2.seerstage#c.age | 22.51 | 23.16 | 2.00 | 0.87 |
| 3.seerstage#c.age | 10.58 | 9.79 | 4.10 | 0.80 |
| 4.seerstage#c.age | 7.15 | 7.15 | 0.00 | 1.00 |
| 2.race#1.surgery | 0.04 | 0.03 | 7.10 | 0.52 |
| 3.race#1.surgery | 0.01 | 0.01 | 0.00 | 1.00 |
| 4.race#1.surgery | 0.03 | 0.04 | 4.80 | 0.74 |

LABP: lung cancer with subsequent bladder cancer; bias: absolutely standardized difference; p: t test between matched treated and control group

Tab S23. balance test between group mTPC2 with LABP and single lung cancer in the matched cohort

|  | Mean | |  |  |
| --- | --- | --- | --- | --- |
| Variable | Treated | Control | %bias | p |
| age | 65.70 | 65.19 | 5.20 | 0.73 |
| 1.surgery | 0.94 | 0.94 | 0.00 | 1.00 |
| 2.seerstage | 0.21 | 0.20 | 3.60 | 0.85 |
| 3.seerstage | 0.01 | 0.01 | 0.00 | 1.00 |
| 4.seerstage | 0.19 | 0.19 | 0.00 | 1.00 |
| 3.race | 0.04 | 0.05 | 6.00 | 0.70 |
| 4.race | 0.03 | 0.03 | 0.00 | 1.00 |
| 2.category | 0.45 | 0.44 | 2.50 | 0.88 |
| 3.category | 0.36 | 0.38 | 2.70 | 0.87 |
| 1.marital | 0.80 | 0.80 | 0.00 | 1.00 |
| 2.marital | 0.01 | 0.01 | 0.00 | 1.00 |
| 2.category#2.seerstage | 0.09 | 0.08 | 5.20 | 0.77 |
| 2.category#3.seerstage | 0.01 | 0.01 | 0.00 | 1.00 |
| 3.category#2.seerstage | 0.04 | 0.04 | 0.00 | 1.00 |
| 3.category#4.seerstage | 0.19 | 0.19 | 0.00 | 1.00 |
| 2.category#3.race | 0.01 | 0.01 | 0.00 | 1.00 |
| 3.category#3.race | 0.03 | 0.04 | 9.50 | 0.65 |
| 3.category#4.race | 0.03 | 0.03 | 0.00 | 1.00 |
| 2.category#c.age | 30.83 | 30.03 | 2.40 | 0.88 |
| 3.category#c.age | 21.64 | 22.24 | 2.10 | 0.90 |
| 2.seerstage#c.age | 13.68 | 12.44 | 5.50 | 0.77 |
| 3.seerstage#c.age | 0.78 | 0.78 | 0.00 | 1.00 |
| 4.seerstage#c.age | 11.23 | 11.19 | 0.20 | 0.99 |
| c.age#c.age | 4400.50 | 4334.90 | 5.20 | 0.73 |
| 1.surgery#c.age | 60.99 | 60.48 | 2.30 | 0.86 |
| 1.marital#2.seerstage | 0.18 | 0.16 | 3.90 | 0.83 |
| 1.marital#3.seerstage | 0.01 | 0.01 | 0.00 | 1.00 |
| 1.marital#4.seerstage | 0.15 | 0.15 | 0.00 | 1.00 |
| 3.race#2.seerstage | 0.01 | 0.01 | 0.00 | 1.00 |
| 4.race#4.seerstage | 0.01 | 0.01 | 0.00 | 1.00 |

LABP: lung cancer with subsequent bladder cancer; bias: absolutely standardized difference; p: t test between matched treated and control group

Tab S24. balance test between LABP and single bladder cancer in the matched cohort

|  | Mean | |  |  |
| --- | --- | --- | --- | --- |
| Variable | Treated | Control | %bias | p |
| age | 72.97 | 73.08 | 1.00 | 0.87 |
| 2.race | 0.04 | 0.05 | 1.50 | 0.85 |
| 3.race | 0.02 | 0.02 | 0.00 | 1.00 |
| 4.race | 0.02 | 0.02 | 1.60 | 0.79 |
| 2.seerstage | 0.06 | 0.06 | 1.10 | 0.87 |
| 3.seerstage | 0.01 | 0.01 | 0.00 | 1.00 |
| 4.seerstage | 0.10 | 0.10 | 1.00 | 0.90 |
| 1.surgery | 0.93 | 0.94 | 1.30 | 0.88 |
| 1.insurance | 0.51 | 0.51 | 0.00 | 1.00 |
| 2.insurance | 0.49 | 0.49 | 0.60 | 0.94 |
| 2.radiation | 0.97 | 0.96 | 1.70 | 0.84 |
| c.age#c.age | 5400.20 | 5414.30 | 0.90 | 0.88 |
| 2.seerstage#c.age | 4.38 | 4.22 | 0.90 | 0.91 |
| 3.seerstage#c.age | 0.66 | 0.66 | 0.00 | 1.00 |
| 4.seerstage#c.age | 8.19 | 8.03 | 0.70 | 0.93 |
| 1.insurance#2.seerstage | 0.03 | 0.03 | 0.00 | 1.00 |
| 1.insurance#3.seerstage | 0.01 | 0.01 | 0.00 | 1.00 |
| 1.insurance#4.seerstage | 0.09 | 0.09 | 1.10 | 0.89 |
| 2.insurance#2.seerstage | 0.02 | 0.02 | 0.00 | 1.00 |
| 2.insurance#4.seerstage | 0.01 | 0.01 | 0.00 | 1.00 |
| 1.insurance#2.race | 0.02 | 0.02 | 0.00 | 1.00 |
| 1.insurance#3.race | 0.00 | 0.00 | 0.00 | 1.00 |
| 1.insurance#4.race | 0.02 | 0.01 | 2.00 | 0.76 |
| 2.insurance#2.race | 0.02 | 0.03 | 4.10 | 0.63 |
| 2.insurance#3.race | 0.01 | 0.01 | 0.00 | 1.00 |
| 2.insurance#4.race | 0.01 | 0.01 | 0.00 | 1.00 |
| 1.surgery#2.race | 0.03 | 0.03 | 0.00 | 1.00 |
| 1.surgery#3.race | 0.01 | 0.01 | 0.00 | 1.00 |
| 1.surgery#4.race | 0.02 | 0.02 | 0.00 | 1.00 |
| 2.seerstage#2.race | 0.00 | 0.00 | 4.80 | 0.32 |
| 4.seerstage#2.race | 0.01 | 0.01 | 0.00 | 1.00 |
| 4.seerstage#3.race | 0.00 | 0.00 | 0.00 | 1.00 |
| 4.seerstage#4.race | 0.01 | 0.00 | 4.00 | 0.56 |
| 2.race#c.age | 2.97 | 3.25 | 2.10 | 0.80 |
| 3.race#c.age | 1.20 | 1.20 | 0.10 | 0.99 |
| 4.race#c.age | 1.65 | 1.45 | 1.50 | 0.81 |
| 1.insurance#1.surgery | 0.49 | 0.50 | 0.60 | 0.94 |
| 2.insurance#1.surgery | 0.44 | 0.44 | 0.60 | 0.94 |

LABP: lung cancer with subsequent bladder cancer; bias: absolutely standardized difference; p: t test between matched treated and control group

Tab S25. balance test between group sTPC with LABP and single bladder cancer in the matched cohort

|  | Mean | |  |  |
| --- | --- | --- | --- | --- |
| Variable | Treated | Control | %bias | p |
| age | 71.051 | 71.051 | 0 | 1 |
| 2.race | 0.0339 | 0.0339 | 0 | 1 |
| 3.race | 0.00847 | 0.00847 | 0 | 1 |
| 1.surgery | 0.91525 | 0.91525 | 0 | 1 |
| 2.radiation | 0.94068 | 0.94068 | 0 | 1 |
| c.age#c.age | 5107.4 | 5107.4 | 0 | 1 |
| 1.surgery#2.race | 0.01695 | 0.01695 | 0 | 1 |
| 1.surgery#3.race | 0.00847 | 0.00847 | 0 | 1 |

LABP: lung cancer with subsequent bladder cancer; bias: absolutely standardized difference; p: t test between matched treated and control group

Tab S26. balance test between group mTPC1 with LABP and single bladder cancer in the matched cohort

|  | Mean | |  |  |
| --- | --- | --- | --- | --- |
| Variable | Treated | Control | %bias | p |
| age | 72.85 | 72.87 | 0.10 | 0.99 |
| 2.seerstage | 0.04 | 0.04 | 0.00 | 1.00 |
| 4.seerstage | 0.12 | 0.12 | 0.00 | 1.00 |
| 2.race | 0.08 | 0.08 | 3.10 | 0.83 |
| 3.race | 0.01 | 0.01 | 0.00 | 1.00 |
| 4.race | 0.04 | 0.03 | 3.40 | 0.76 |
| 1.surgery | 0.93 | 0.94 | 3.10 | 0.81 |
| 1.insurance | 0.50 | 0.50 | 0.00 | 1.00 |
| 2.insurance | 0.50 | 0.50 | 0.00 | 1.00 |
| c.age#c.age | 5393.10 | 5395.10 | 0.10 | 0.99 |
| 2.seerstage#c.age | 3.09 | 3.09 | 0.00 | 1.00 |
| 4.seerstage#c.age | 9.41 | 9.42 | 0.10 | 1.00 |
| 2.race#4.seerstage | 0.01 | 0.02 | 7.70 | 0.65 |
| 3.race#4.seerstage | 0.01 | 0.01 | 0.00 | 1.00 |
| 4.race#4.seerstage | 0.01 | 0.01 | 7.30 | 0.56 |
| 2.race#c.age | 5.29 | 5.78 | 3.10 | 0.83 |
| 3.race#c.age | 0.86 | 0.86 | 0.00 | 1.00 |
| 4.race#c.age | 2.94 | 2.47 | 3.30 | 0.77 |
| 1.insurance#1.surgery | 0.50 | 0.50 | 1.40 | 0.91 |
| 2.insurance#1.surgery | 0.43 | 0.43 | 0.00 | 1.00 |
| 1.surgery#2.race | 0.06 | 0.06 | 3.40 | 0.80 |
| 1.surgery#3.race | 0.01 | 0.01 | 0.00 | 1.00 |
| 1.surgery#4.race | 0.03 | 0.03 | 0.00 | 1.00 |
| 1.insurance#2.race | 0.04 | 0.05 | 4.10 | 0.78 |
| 1.insurance#3.race | 0.01 | 0.01 | 0.00 | 1.00 |
| 1.insurance#4.race | 0.03 | 0.03 | 4.00 | 0.74 |
| 2.insurance#2.race | 0.03 | 0.03 | 0.00 | 1.00 |
| 2.insurance#3.race | 0.01 | 0.01 | 0.00 | 1.00 |
| 2.insurance#4.race | 0.01 | 0.01 | 0.00 | 1.00 |

LABP: lung cancer with subsequent bladder cancer; bias: absolutely standardized difference; p: t test between matched treated and control group

Tab S27. balance test between group mTPC2 with LABP and single bladder cancer in the matched cohort

|  | Mean | |  |  |
| --- | --- | --- | --- | --- |
| Variable | Treated | Control | %bias | p |
| age | 75.94 | 75.94 | 0.00 | 1.00 |
| 3.race | 0.04 | 0.04 | 0.00 | 1.00 |
| 4.race | 0.02 | 0.02 | 0.00 | 1.00 |
| 2.category | 0.23 | 0.23 | 0.00 | 1.00 |
| 3.category | 0.10 | 0.10 | 0.00 | 1.00 |
| 2.seerstage | 0.05 | 0.05 | 0.00 | 1.00 |
| 3.seerstage | 0.01 | 0.01 | 0.00 | 1.00 |
| 4.seerstage | 0.15 | 0.15 | 0.00 | 1.00 |
| c.age#c.age | 5834.10 | 5834.10 | 0.00 | 1.00 |
| 2.category#3.race | 0.02 | 0.02 | 0.00 | 1.00 |
| 3.category#4.race | 0.01 | 0.01 | 0.00 | 1.00 |
| 2.seerstage#2.category | 0.01 | 0.01 | 0.00 | 1.00 |
| 2.seerstage#3.category | 0.01 | 0.01 | 0.00 | 1.00 |
| 2.category#c.age | 17.26 | 17.26 | 0.00 | 1.00 |
| 3.category#c.age | 7.00 | 7.00 | 0.00 | 1.00 |

LABP: lung cancer with subsequent bladder cancer; bias: absolutely standardized difference; p: t test between matched treated and control group.

Tab S28. balance test between BPLA and single lung cancer in the matched cohort

| Variable | Mean | |  |  |
| --- | --- | --- | --- | --- |
|  | Treated | Control | bias | p |
| age | 70.91 | 70.50 | 4.20 | 0.70 |
| 2.seerstage | 0.27 | 0.28 | 1.80 | 0.89 |
| 3.seerstage | 0.40 | 0.40 | 0.00 | 1.00 |
| 4.seerstage | 0.18 | 0.18 | 0.00 | 1.00 |
| 2.race | 0.03 | 0.03 | 0.00 | 1.00 |
| 3.race | 0.01 | 0.01 | 0.00 | 1.00 |
| 1.surgery | 0.34 | 0.33 | 1.90 | 0.89 |
| 2.surgery | 0.03 | 0.03 | 0.00 | 1.00 |
| 1.radiation | 0.48 | 0.49 | 1.70 | 0.90 |
| 2.radiation | 0.50 | 0.50 | 1.70 | 0.90 |
| 2.category | 0.27 | 0.26 | 3.70 | 0.77 |
| 3.category | 0.19 | 0.21 | 4.20 | 0.74 |
| 1.marital | 0.74 | 0.74 | 0.00 | 1.00 |
| 1.surgery#2.seerstage | 0.15 | 0.15 | 0.00 | 1.00 |
| 1.surgery#3.seerstage | 0.05 | 0.04 | 5.40 | 0.76 |
| 1.surgery#4.seerstage | 0.06 | 0.06 | 0.00 | 1.00 |
| 2.surgery#2.seerstage | 0.01 | 0.01 | 0.00 | 1.00 |
| 2.surgery#4.seerstage | 0.02 | 0.02 | 0.00 | 1.00 |
| c.age#c.age | 5087.10 | 5033.60 | 4.20 | 0.71 |
| 2.seerstage#c.age | 19.24 | 19.90 | 2.00 | 0.88 |
| 3.seerstage#c.age | 27.89 | 27.50 | 1.50 | 0.93 |
| 4.seerstage#c.age | 12.86 | 12.86 | 0.00 | 1.00 |
| 1.marital#2.seerstage | 0.17 | 0.18 | 2.00 | 0.86 |
| 1.marital#3.seerstage | 0.30 | 0.31 | 2.50 | 0.89 |
| 1.marital#4.seerstage | 0.15 | 0.15 | 0.00 | 1.00 |
| 1.marital#2.category | 0.21 | 0.19 | 4.20 | 0.74 |
| 1.marital#3.category | 0.12 | 0.12 | 0.00 | 1.00 |
| 2.category#1.surgery | 0.09 | 0.09 | 3.00 | 0.82 |
| 2.category#2.surgery | 0.01 | 0.01 | 0.00 | 1.00 |
| 3.category#1.surgery | 0.07 | 0.07 | 0.00 | 1.00 |
| 3.category#2.surgery | 0.02 | 0.02 | 0.00 | 1.00 |
| 2.category#2.race | 0.02 | 0.02 | 0.00 | 1.00 |
| 2.category#3.race | 0.01 | 0.01 | 0.00 | 1.00 |
| 2.race#2.seerstage | 0.01 | 0.01 | 0.00 | 1.00 |
| 2.race#3.seerstage | 0.02 | 0.02 | 0.00 | 1.00 |
| 2.race#4.seerstage | 0.01 | 0.01 | 0.00 | 1.00 |
| 3.race#2.seerstage | 0.01 | 0.01 | 0.00 | 1.00 |

BPLA: bladder cancer with subsequent lung cancer; bias: absolutely standardized difference; p: t test between matched treated and control group.

Tab S29.balance test between group sTPC with BPLA and single lung cancer in the matched cohort

| Variable | Mean | |  |  |
| --- | --- | --- | --- | --- |
|  | Treated | Control | bias | p |
| age | 70.91 | 70.50 | 4.20 | 0.70 |
| 2.seerstage | 0.27 | 0.28 | 1.80 | 0.89 |
| 3.seerstage | 0.40 | 0.40 | 0.00 | 1.00 |
| 4.seerstage | 0.18 | 0.18 | 0.00 | 1.00 |
| 2.race | 0.03 | 0.03 | 0.00 | 1.00 |
| 3.race | 0.01 | 0.01 | 0.00 | 1.00 |
| 1.surgery | 0.34 | 0.33 | 1.90 | 0.89 |
| 2.surgery | 0.03 | 0.03 | 0.00 | 1.00 |
| 1.radiation | 0.48 | 0.49 | 1.70 | 0.90 |
| 2.radiation | 0.50 | 0.50 | 1.70 | 0.90 |
| 2.category | 0.27 | 0.26 | 3.70 | 0.77 |
| 3.category | 0.19 | 0.21 | 4.20 | 0.74 |
| 1.marital | 0.74 | 0.74 | 0.00 | 1.00 |
| 1.surgery#2.seerstage | 0.15 | 0.15 | 0.00 | 1.00 |
| 1.surgery#3.seerstage | 0.05 | 0.04 | 5.40 | 0.76 |
| 1.surgery#4.seerstage | 0.06 | 0.06 | 0.00 | 1.00 |
| 2.surgery#2.seerstage | 0.01 | 0.01 | 0.00 | 1.00 |
| 2.surgery#4.seerstage | 0.02 | 0.02 | 0.00 | 1.00 |
| c.age#c.age | 5087.10 | 5033.60 | 4.20 | 0.71 |
| 2.seerstage#c.age | 19.24 | 19.90 | 2.00 | 0.88 |
| 3.seerstage#c.age | 27.89 | 27.50 | 1.50 | 0.93 |
| 4.seerstage#c.age | 12.86 | 12.86 | 0.00 | 1.00 |
| 1.marital#2.seerstage | 0.17 | 0.18 | 2.00 | 0.86 |
| 1.marital#3.seerstage | 0.30 | 0.31 | 2.50 | 0.89 |
| 1.marital#4.seerstage | 0.15 | 0.15 | 0.00 | 1.00 |
| 1.marital#2.category | 0.21 | 0.19 | 4.20 | 0.74 |
| 1.marital#3.category | 0.12 | 0.12 | 0.00 | 1.00 |
| 2.category#1.surgery | 0.09 | 0.09 | 3.00 | 0.82 |
| 2.category#2.surgery | 0.01 | 0.01 | 0.00 | 1.00 |
| 3.category#1.surgery | 0.07 | 0.07 | 0.00 | 1.00 |
| 3.category#2.surgery | 0.02 | 0.02 | 0.00 | 1.00 |
| 2.category#2.race | 0.02 | 0.02 | 0.00 | 1.00 |
| 2.category#3.race | 0.01 | 0.01 | 0.00 | 1.00 |
| 2.race#2.seerstage | 0.01 | 0.01 | 0.00 | 1.00 |
| 2.race#3.seerstage | 0.02 | 0.02 | 0.00 | 1.00 |
| 2.race#4.seerstage | 0.01 | 0.01 | 0.00 | 1.00 |
| 3.race#2.seerstage | 0.01 | 0.01 | 0.00 | 1.00 |

BPLA: bladder cancer with subsequent lung cancer; bias: absolutely standardized difference; p: t test between matched treated and control group.

Tab S30.balance test between group mTPC1 with BPLA and single lung cancer in the matched cohort

| Variable | Mean | |  |  |
| --- | --- | --- | --- | --- |
|  | Treated | Control | bias | p |
| age | 70.44 | 70.94 | 5.10 | 0.65 |
| 2.seerstage | 0.32 | 0.33 | 1.50 | 0.90 |
| 3.seerstage | 0.15 | 0.14 | 5.30 | 0.73 |
| 4.seerstage | 0.11 | 0.11 | 0.00 | 1.00 |
| 1.surgery | 0.57 | 0.57 | 0.00 | 1.00 |
| 2.surgery | 0.01 | 0.01 | 0.00 | 1.00 |
| 2.race | 0.08 | 0.07 | 2.40 | 0.82 |
| 3.race | 0.01 | 0.01 | 0.00 | 1.00 |
| 4.race | 0.04 | 0.04 | 0.00 | 1.00 |
| 1.radiation | 0.41 | 0.40 | 1.40 | 0.90 |
| 2.radiation | 0.59 | 0.60 | 1.40 | 0.90 |
| 1.insurance | 0.44 | 0.41 | 5.70 | 0.63 |
| 2.insurance | 0.56 | 0.59 | 5.70 | 0.63 |
| 2.category | 0.33 | 0.36 | 6.10 | 0.62 |
| 3.category | 0.20 | 0.21 | 1.80 | 0.88 |
| 2.category#2.seerstage | 0.11 | 0.14 | 6.70 | 0.59 |
| 2.category#3.seerstage | 0.05 | 0.05 | 0.00 | 1.00 |
| 3.category#2.seerstage | 0.04 | 0.04 | 3.90 | 0.76 |
| 3.category#4.seerstage | 0.11 | 0.11 | 0.00 | 1.00 |
| 1.surgery#2.seerstage | 0.22 | 0.23 | 2.20 | 0.89 |
| 1.surgery#3.seerstage | 0.01 | 0.00 | 15.70 | 0.16 |
| 1.surgery#4.seerstage | 0.04 | 0.04 | 0.00 | 1.00 |
| 2.surgery#4.seerstage | 0.01 | 0.01 | 0.00 | 1.00 |
| 1.radiation#1.surgery | 0.11 | 0.11 | 0.00 | 1.00 |
| 1.radiation#2.surgery | 0.01 | 0.01 | 0.00 | 1.00 |
| 2.radiation#1.surgery | 0.46 | 0.46 | 0.00 | 1.00 |
| 2.race#2.seerstage | 0.01 | 0.00 | 8.00 | 0.16 |
| 2.race#3.seerstage | 0.03 | 0.04 | 5.80 | 0.74 |
| 2.race#4.seerstage | 0.01 | 0.01 | 0.00 | 1.00 |
| 4.race#2.seerstage | 0.01 | 0.02 | 5.00 | 0.65 |
| 4.race#3.seerstage | 0.01 | 0.00 | 10.70 | 0.32 |
| 1.radiation#2.seerstage | 0.14 | 0.14 | 1.90 | 0.86 |
| 1.radiation#3.seerstage | 0.09 | 0.07 | 7.00 | 0.66 |
| 1.radiation#4.seerstage | 0.07 | 0.07 | 0.00 | 1.00 |
| 2.radiation#2.seerstage | 0.19 | 0.19 | 0.00 | 1.00 |
| 2.radiation#3.seerstage | 0.06 | 0.06 | 0.00 | 1.00 |
| 2.radiation#4.seerstage | 0.04 | 0.04 | 0.00 | 1.00 |
| 2.race#c.age | 5.24 | 4.72 | 2.70 | 0.81 |
| 3.race#c.age | 0.84 | 0.84 | 0.00 | 1.00 |
| 4.race#c.age | 2.90 | 2.96 | 0.40 | 0.97 |
| 2.seerstage#c.age | 22.51 | 23.16 | 2.00 | 0.87 |
| 3.seerstage#c.age | 10.58 | 9.79 | 4.10 | 0.80 |
| 4.seerstage#c.age | 7.15 | 7.15 | 0.00 | 1.00 |
| 2.race#1.surgery | 0.04 | 0.03 | 7.10 | 0.52 |
| 3.race#1.surgery | 0.01 | 0.01 | 0.00 | 1.00 |
| 4.race#1.surgery | 0.03 | 0.04 | 4.80 | 0.74 |

BPLA: bladder cancer with subsequent lung cancer; bias: absolutely standardized difference; p: t test between matched treated and control group.

Tab S31.balance test between group mTPC2 with BPLA and single lung cancer in the matched cohort

| Variable | Mean | |  |  |
| --- | --- | --- | --- | --- |
|  | Treated | Control | bias | p |
| age | 65.7 65 | 65.19 | 5.20 | 0.73 |
| 1.surgery | 0.94 | 0.94 | 0.00 | 1.00 |
| 2.seerstage | 0.21 | 0.20 | 3.60 | 0.85 |
| 3.seerstage | 0.01 | 0.01 | 0.00 | 1.00 |
| 4.seerstage | 0.19 | 0.19 | 0.00 | 1.00 |
| 3.race | 0.04 | 0.05 | 6.00 | 0.70 |
| 4.race | 0.03 | 0.03 | 0.00 | 1.00 |
| 2.category | .45 .4 | 0.44 | 2.50 | 0.88 |
| 3.category | 0.36 | 0.38 | 2.70 | 0.87 |
| 1.marital | 0.80 | 0.80 | 0.00 | 1.00 |
| 2.marital | 0.01 | 0.01 | 0.00 | 1.00 |
| 2.category#2.seerstage | 0.09 | 0.08 | 5.20 | 0.77 |
| 2.category#3.seerstage | 0.01 | 0.01 | 0.00 | 1.00 |
| 3.category#2.seerstage | 0.04 | 0.04 | 0.00 | 1.00 |
| 3.category#4.seerstage | 0.19 | 0.19 | 0.00 | 1.00 |
| 2.category#3.race | 0.01 | 0.01 | 0.00 | 1.00 |
| 3.category#3.race | .025 . | 0.04 | 9.50 | 0.65 |
| 3.category#4.race | 0.03 | 0.03 | 0.00 | 1.00 |
| 2.category#c.age | 30.83 | 30.03 | 2.40 | 0.88 |
| 3.category#c.age | 21.64 | 22.24 | 2.10 | 0.90 |
| 2.seerstage#c.age | 13.68 | 12.44 | 5.50 | 0.77 |
| 3.seerstage#c.age | 0.78 | 0.78 | 0.00 | 1.00 |
| 4.seerstage#c.age | 11.23 | 11.19 | 0.20 | 0.99 |
| c.age#c.age | 4400.50 | 4334.90 | 5.20 | 0.73 |
| 1.surgery#c.age | 60.99 | 60.48 | 2.30 | 0.86 |
| 1.marital#2.seerstage | .175 . | 0.16 | 3.90 | 0.83 |
| 1.marital#3.seerstage | 0.01 | 0.01 | 0.00 | 1.00 |
| 1.marital#4.seerstage | 0.15 | 0.15 | 0.00 | 1.00 |
| 3.race#2.seerstage | 0.01 | 0.01 | 0.00 | 1.00 |
| 4.race#4.seerstage | 0.01 | 0.01 | 0.00 | 1.00 |

BPLA: bladder cancer with subsequent lung cancer; bias: absolutely standardized difference; p: t test between matched treated and control group.

Tab S32.balance test between BPLA and single bladder cancer in the matched cohort

| Variable | Mean | |  |  |
| --- | --- | --- | --- | --- |
|  | Treated | Control | bias | p |
| age | 72.97 | 73.08 | 1.00 | 0.87 |
| 2.race | 0.04 | 0.05 | 1.50 | 0.85 |
| 3.race | 0.02 | 0.02 | 0.00 | 1.00 |
| 4.race | 0.02 | 0.02 | 1.60 | 0.79 |
| 2.seerstage | 0.06 | 0.06 | 1.10 | 0.87 |
| 3.seerstage | 0.01 | 0.01 | 0.00 | 1.00 |
| 4.seerstage | 0.10 | 0.10 | 1.00 | 0.90 |
| 1.surgery | 0.93 | 0.94 | 1.30 | 0.88 |
| 1.insurance | 0.51 | 0.51 | 0.00 | 1.00 |
| 2.insurance | 0.49 | 0.49 | 0.60 | 0.94 |
| 2.radiation | 0.97 | 0.96 | 1.70 | 0.84 |
| c.age#c.age | 5400.20 | 5414.30 | 0.90 | 0.88 |
| 2.seerstage#c.age | 4.38 | 4.22 | 0.90 | 0.91 |
| 3.seerstage#c.age | 0.66 | 0.66 | 0.00 | 1.00 |
| 4.seerstage#c.age | 8.19 | 8.03 | 0.70 | 0.93 |
| 1.insurance#2.seerstage | 0.03 | 0.03 | 0.00 | 1.00 |
| 1.insurance#3.seerstage | 0.01 | 0.01 | 0.00 | 1.00 |
| 1.insurance#4.seerstage | 0.09 | 0.09 | 1.10 | 0.89 |
| 2.insurance#2.seerstage | 0.02 | 0.02 | 0.00 | 1.00 |
| 2.insurance#4.seerstage | 0.01 | 0.01 | 0.00 | 1.00 |
| 1.insurance#2.race | 0.02 | 0.02 | 0.00 | 1.00 |
| 1.insurance#3.race | 0.00 | 0.00 | 0.00 | 1.00 |
| 1.insurance#4.race | 0.02 | 0.01 | 2.00 | 0.76 |
| 2.insurance#2.race | 0.02 | 0.03 | 4.10 | 0.63 |
| 2.insurance#3.race | 0.01 | 0.01 | 0.00 | 1.00 |
| 2.insurance#4.race | 0.01 | 0.01 | 0.00 | 1.00 |
| 1.surgery#2.race | 0.03 | 0.03 | 0.00 | 1.00 |
| 1.surgery#3.race | 0.01 | 0.01 | 0.00 | 1.00 |
| 1.surgery#4.race | 0.02 | 0.02 | 0.00 | 1.00 |
| 2.seerstage#2.race | 0.00 | 0.00 | 4.80 | 0.32 |
| 4.seerstage#2.race | 0.01 | 0.01 | 0.00 | 1.00 |
| 4.seerstage#3.race | 0.00 | 0.00 | 0.00 | 1.00 |
| 4.seerstage#4.race | 0.01 | 0.00 | 4.00 | 0.56 |
| 2.race#c.age | 2.97 | 3.25 | 2.10 | 0.80 |
| 3.race#c.age | 1.20 | 1.20 | 0.10 | 0.99 |
| 4.race#c.age | 1.65 | 1.45 | 1.50 | 0.81 |
| 1.insurance#1.surgery | 0.49 | 0.50 | 0.60 | 0.94 |
| 2.insurance#1.surgery | 0.44 | 0.44 | 0.60 | 0.94 |

BPLA: bladder cancer with subsequent lung cancer; bias: absolutely standardized difference; p: t test between matched treated and control group.

Tab S33.balance test between group sTPC with BPLA and single bladder cancer in the matched cohort

| Variable | Mean | |  |  |
| --- | --- | --- | --- | --- |
|  | Treated | Control | bias | p |
| age | 71.05 | 71.05 | 0.00 | 1.00 |
| 2.race | 0.03 | 0.03 | 0.00 | 1.00 |
| 3.race | 0.01 | 0.01 | 0.00 | 1.00 |
| 1.surgery | 0.92 | 0.92 | 0.00 | 1.00 |
| 2.radiation | 0.94 | 0.94 | 0.00 | 1.00 |
| c.age#c.age | 5107.40 | 5107.40 | 0.00 | 1.00 |
| 1.surgery#2.race | 0.02 | 0.02 | 0.00 | 1.00 |
| 1.surgery#3.race | 0.01 | 0.01 | 0.00 | 1.00 |

BPLA: bladder cancer with subsequent lung cancer; bias: absolutely standardized difference; p: t test between matched treated and control group.

Tab S34.balance test between group mTPC1 with BPLA and single bladder cancer in the matched cohort

| Variable | Mean | |  |  |
| --- | --- | --- | --- | --- |
|  | Treated | Control | bias | p |
| age | 72.85 | 72.87 | 0.10 | 0.99 |
| 2.seerstage | 0.04 | 0.04 | 0.00 | 1.00 |
| 4.seerstage | 0.12 | 0.12 | 0.00 | 1.00 |
| 2.race | 0.08 | 0.08 | 3.10 | 0.83 |
| 3.race | 0.01 | 0.01 | 0.00 | 1.00 |
| 4.race | 0.04 | 0.03 | 3.40 | 0.76 |
| 1.surgery | 0.93 | 0.94 | 3.10 | 0.81 |
| 1.insurance | 0.50 | 0.50 | 0.00 | 1.00 |
| 2.insurance | 0.50 | 0.50 | 0.00 | 1.00 |
| c.age#c.age | 5393.10 | 5395.10 | 0.10 | 0.99 |
| 2.seerstage#c.age | 3.09 | 3.09 | 0.00 | 1.00 |
| 4.seerstage#c.age | 9.41 | 9.42 | 0.10 | 1.00 |
| 2.race#4.seerstage | 0.01 | 0.02 | 7.70 | 0.65 |
| 3.race#4.seerstage | 0.01 | 0.01 | 0.00 | 1.00 |
| 4.race#4.seerstage | 0.01 | 0.01 | 7.30 | 0.56 |
| 2.race#c.age | 5.29 | 5.78 | 3.10 | 0.83 |
| 3.race#c.age | 0.86 | 0.86 | 0.00 | 1.00 |
| 4.race#c.age | 2.94 | 2.47 | 3.30 | 0.77 |
| 1.insurance#1.surgery | 0.50 | 0.50 | 1.40 | 0.91 |
| 2.insurance#1.surgery | 0.43 | 0.43 | 0.00 | 1.00 |
| 1.surgery#2.race | 0.06 | 0.06 | 3.40 | 0.80 |
| 1.surgery#3.race | 0.01 | 0.01 | 0.00 | 1.00 |
| 1.surgery#4.race | 0.03 | 0.03 | 0.00 | 1.00 |
| 1.insurance#2.race | 0.04 | 0.05 | 4.10 | 0.78 |
| 1.insurance#3.race | 0.01 | 0.01 | 0.00 | 1.00 |
| 1.insurance#4.race | 0.03 | 0.03 | 4.00 | 0.74 |
| 2.insurance#2.race | 0.03 | 0.03 | 0.00 | 1.00 |
| 2.insurance#3.race | 0.01 | 0.01 | 0.00 | 1.00 |
| 2.insurance#4.race | 0.01 | 0.01 | 0.00 | 1.00 |

BPLA: bladder cancer with subsequent lung cancer; bias: absolutely standardized difference; p: t test between matched treated and control group.

Tab S35.balance test between group mTPC2 with BPLA and single bladder cancer in the matched cohort

| Variable | Mean | |  |  |
| --- | --- | --- | --- | --- |
|  | Treated | Control | bias | p |
| age | 75.94 | 75.94 | 0.00 | 1.00 |
| 3.race | 0.04 | 0.04 | 0.00 | 1.00 |
| 4.race | 0.02 | 0.02 | 0.00 | 1.00 |
| 2.category | 0.23 | 0.23 | 0.00 | 1.00 |
| 3.category | 0.10 | 0.10 | 0.00 | 1.00 |
| 2.seerstage | 0.05 | 0.05 | 0.00 | 1.00 |
| 3.seerstage | 0.01 | 0.01 | 0.00 | 1.00 |
| 4.seerstage | 0.15 | 0.15 | 0.00 | 1.00 |
| c.age#c.age | 5834.10 | 5834.10 | 0.00 | 1.00 |
| 2.category#3.race | 0.02 | 0.02 | 0.00 | 1.00 |
| 3.category#4.race | 0.01 | 0.01 | 0.00 | 1.00 |
| 2.seerstage#2.category | 0.01 | 0.01 | 0.00 | 1.00 |
| 2.seerstage#3.category | 0.01 | 0.01 | 0.00 | 1.00 |
| 2.category#c.age | 17.26 | 17.26 | 0.00 | 1.00 |
| 3.category#c.age | 7.00 | 7.00 | 0.00 | 1.00 |

BPLA: bladder cancer with subsequent lung cancer; bias: absolutely standardized difference; p: t test between matched treated and control group.

Tab S36.balance test LAPA and single lung cancer in the matched cohort

| Variable | Mean | |  |  |
| --- | --- | --- | --- | --- |
|  | Treated | Control | bias | P |
| age | 66.98 | 66.98 | 0.00 | 1.00 |
| 1.surgery | 0.66 | 0.66 | 0.80 | 0.86 |
| 2.surgery | 0.01 | 0.01 | 0.80 | 0.84 |
| 2.seerstage | 0.24 | 0.24 | 0.60 | 0.88 |
| 3.seerstage | 0.17 | 0.17 | 0.60 | 0.91 |
| 4.seerstage | 0.24 | 0.24 | 0.70 | 0.88 |
| 2.race | 0.18 | 0.18 | 0.20 | 0.96 |
| 3.race | 0.04 | 0.03 | 3.60 | 0.28 |
| 4.race | 0.04 | 0.04 | 0.40 | 0.92 |
| 2.category | 0.31 | 0.31 | 1.30 | 0.75 |
| 3.category | 0.38 | 0.39 | 1.90 | 0.67 |
| 1.marital | 0.72 | 0.73 | 2.40 | 0.54 |
| 2.marital | 0.02 | 0.02 | 3.20 | 0.36 |
| 1.radiation | 0.28 | 0.28 | 0.20 | 0.96 |
| 2.radiation | 0.72 | 0.72 | 0.20 | 0.96 |
| 1.insurance | 0.26 | 0.26 | 1.90 | 0.63 |
| 2.insurance | 0.73 | 0.74 | 2.20 | 0.57 |
| 2.seerstage#1.surgery | 0.18 | 0.18 | 1.20 | 0.83 |
| 3.seerstage#1.surgery | 0.02 | 0.01 | 4.40 | 0.41 |
| 4.seerstage#1.surgery | 0.14 | 0.15 | 0.90 | 0.86 |
| 4.seerstage#2.surgery | 0.01 | 0.01 | 0.00 | 1.00 |
| 2.category#2.seerstage | 0.10 | 0.10 | 0.50 | 0.89 |
| 2.category#3.seerstage | 0.06 | 0.06 | 0.50 | 0.93 |
| 3.category#2.seerstage | 0.04 | 0.05 | 1.60 | 0.69 |
| 3.category#3.seerstage | 0.04 | 0.04 | 2.40 | 0.66 |
| 3.category#4.seerstage | 0.22 | 0.22 | 0.50 | 0.92 |
| 1.radiation#2.seerstage | 0.10 | 0.10 | 0.00 | 1.00 |
| 1.radiation#3.seerstage | 0.08 | 0.08 | 0.40 | 0.94 |
| 1.radiation#4.seerstage | 0.08 | 0.08 | 0.00 | 1.00 |
| 2.radiation#2.seerstage | 0.14 | 0.14 | 0.60 | 0.86 |
| 2.radiation#3.seerstage | 0.09 | 0.09 | 1.20 | 0.83 |
| 2.radiation#4.seerstage | 0.16 | 0.17 | 0.80 | 0.87 |
| c.age#c.age | 4560.00 | 4557.70 | 0.20 | 0.96 |
| 1.radiation#1.surgery | 0.10 | 0.10 | 1.90 | 0.68 |
| 1.radiation#2.surgery | 0.01 | 0.01 | 1.00 | 0.81 |
| 2.radiation#1.surgery | 0.56 | 0.56 | 0.40 | 0.93 |
| 2.radiation#2.surgery | 0.00 | 0.00 | 0.00 | 1.00 |
| 1.radiation#2.category | 0.08 | 0.08 | 0.30 | 0.94 |
| 1.radiation#3.category | 0.11 | 0.12 | 1.90 | 0.64 |
| 2.radiation#2.category | 0.23 | 0.23 | 1.30 | 0.77 |
| 2.radiation#3.category | 0.27 | 0.27 | 0.70 | 0.89 |
| 2.seerstage#c.age | 16.01 | 15.84 | 0.60 | 0.89 |
| 3.seerstage#c.age | 11.89 | 11.78 | 0.50 | 0.92 |
| 4.seerstage#c.age | 15.90 | 16.06 | 0.60 | 0.89 |
| 1.surgery#c.age | 43.46 | 43.22 | 0.80 | 0.86 |
| 2.surgery#c.age | 0.78 | 0.72 | 0.80 | 0.83 |
| 2.race#1.surgery | 0.09 | 0.09 | 0.40 | 0.94 |
| 2.race#2.surgery | 0.00 | 0.00 | 0.00 | 1.00 |
| 3.race#1.surgery | 0.03 | 0.02 | 4.20 | 0.33 |
| 4.race#1.surgery | 0.03 | 0.02 | 2.60 | 0.60 |
| 1.insurance#2.seerstage | 0.08 | 0.07 | 1.70 | 0.57 |
| 1.insurance#3.seerstage | 0.06 | 0.06 | 1.90 | 0.73 |
| 1.insurance#4.seerstage | 0.02 | 0.02 | 0.50 | 0.89 |
| 2.insurance#2.seerstage | 0.16 | 0.17 | 1.30 | 0.73 |
| 2.insurance#3.seerstage | 0.11 | 0.11 | 0.70 | 0.89 |
| 2.insurance#4.seerstage | 0.22 | 0.22 | 0.50 | 0.92 |
| 2.category#2.race | 0.06 | 0.06 | 1.60 | 0.73 |
| 2.category#3.race | 0.01 | 0.01 | 2.00 | 0.55 |
| 2.category#4.race | 0.01 | 0.01 | 0.00 | 1.00 |
| 3.category#2.race | 0.06 | 0.06 | 1.80 | 0.73 |
| 3.category#3.race | 0.02 | 0.02 | 0.70 | 0.87 |
| 3.category#4.race | 0.00 | 0.00 | 2.40 | 0.48 |
| 1.marital#2.race | 0.10 | 0.12 | 5.60 | 0.26 |
| 1.marital#3.race | 0.04 | 0.03 | 4.80 | 0.16 |
| 1.marital#4.race | 0.03 | 0.03 | 0.00 | 1.00 |
| 2.marital#2.race | 0.00 | 0.00 | 2.80 | 0.16 |
| 2.marital#3.race | 0.00 | 0.00 | 2.20 | 0.32 |
| 2.marital#4.race | 0.00 | 0.00 | 2.20 | 0.32 |
| 1.marital#1.surgery | 0.51 | 0.51 | 0.20 | 0.97 |
| 1.marital#2.surgery | 0.01 | 0.01 | 0.90 | 0.83 |
| 2.marital#1.surgery | 0.01 | 0.01 | 2.90 | 0.49 |
| 1.insurance#1.marital | 0.17 | 0.17 | 0.20 | 0.96 |
| 1.insurance#2.marital | 0.01 | 0.01 | 1.60 | 0.64 |
| 2.insurance#1.marital | 0.55 | 0.56 | 2.10 | 0.61 |
| 2.insurance#2.marital | 0.01 | 0.01 | 2.10 | 0.55 |
| 1.marital#2.seerstage | 0.17 | 0.17 | 1.00 | 0.78 |
| 1.marital#3.seerstage | 0.11 | 0.12 | 3.30 | 0.55 |
| 1.marital#4.seerstage | 0.18 | 0.18 | 1.00 | 0.83 |
| 2.marital#2.seerstage | 0.00 | 0.00 | 0.80 | 0.71 |
| 2.marital#3.seerstage | 0.01 | 0.00 | 4.30 | 0.32 |
| 2.marital#4.seerstage | 0.01 | 0.01 | 1.10 | 0.80 |
| 2.category#1.surgery | 0.22 | 0.22 | 0.00 | 1.00 |
| 3.category#1.surgery | 0.25 | 0.26 | 2.00 | 0.70 |
| 3.category#2.surgery | 0.01 | 0.01 | 0.00 | 1.00 |
| 2.race#c.age | 11.38 | 11.30 | 0.40 | 0.94 |
| 3.race#c.age | 2.99 | 2.40 | 3.60 | 0.30 |
| 4.race#c.age | 2.81 | 2.78 | 0.20 | 0.96 |
| 1.insurance#2.race | 0.05 | 0.04 | 3.90 | 0.33 |
| 1.insurance#3.race | 0.01 | 0.01 | 2.20 | 0.41 |
| 1.insurance#4.race | 0.02 | 0.02 | 2.30 | 0.54 |
| 2.insurance#2.race | 0.12 | 0.13 | 2.70 | 0.57 |
| 2.insurance#3.race | 0.03 | 0.03 | 2.90 | 0.45 |
| 2.insurance#4.race | 0.02 | 0.02 | 2.80 | 0.47 |
| 1.radiation#c.age | 19.11 | 19.16 | 0.20 | 0.97 |
| 2.radiation#c.age | 47.87 | 47.82 | 0.20 | 0.97 |
| 2.race#2.seerstage | 0.04 | 0.04 | 1.10 | 0.75 |
| 2.race#3.seerstage | 0.04 | 0.04 | 1.80 | 0.75 |
| 2.race#4.seerstage | 0.04 | 0.04 | 1.00 | 0.83 |
| 3.race#2.seerstage | 0.01 | 0.01 | 2.60 | 0.32 |
| 3.race#3.seerstage | 0.01 | 0.01 | 4.80 | 0.32 |
| 3.race#4.seerstage | 0.01 | 0.01 | 0.00 | 1.00 |
| 4.race#2.seerstage | 0.01 | 0.01 | 1.20 | 0.70 |
| 4.race#3.seerstage | 0.01 | 0.01 | 1.30 | 0.82 |
| 4.race#4.seerstage | 0.00 | 0.00 | 1.30 | 0.71 |
| 1.radiation#2.race | 0.05 | 0.06 | 0.80 | 0.86 |
| 1.radiation#3.race | 0.01 | 0.01 | 3.00 | 0.27 |
| 1.radiation#4.race | 0.01 | 0.01 | 2.10 | 0.55 |
| 2.radiation#2.race | 0.12 | 0.12 | 0.90 | 0.85 |
| 2.radiation#3.race | 0.03 | 0.03 | 2.20 | 0.54 |
| 2.radiation#4.race | 0.03 | 0.03 | 2.00 | 0.62 |

LAPA: lung cancer with subsequent prostate cancer; bias: absolutely standardized difference; p: t test between matched treated and control group.

Tab S37. balance test group sTPC with LAPA and single lung cancer in the matched cohort

| Variable | Mean | |  |  |
| --- | --- | --- | --- | --- |
|  | Treated | Control | bias | P |
| age | 70.07 | 70.01 | 0.60 | 0.93 |
| 2.seerstage | 0.16 | 0.14 | 4.60 | 0.46 |
| 3.seerstage | 0.38 | 0.39 | 3.20 | 0.76 |
| 4.seerstage | 0.30 | 0.31 | 2.10 | 0.81 |
| 2.race | 0.20 | 0.19 | 2.40 | 0.78 |
| 3.race | 0.05 | 0.04 | 3.60 | 0.57 |
| 4.race | 0.04 | 0.03 | 6.80 | 0.31 |
| 1.surgery | 0.31 | 0.28 | 6.50 | 0.41 |
| 2.surgery | 0.03 | 0.03 | 0.00 | 1.00 |
| 2.category | 0.24 | 0.23 | 1.90 | 0.79 |
| 3.category | 0.41 | 0.43 | 2.50 | 0.76 |
| 1.marital | 0.62 | 0.61 | 1.80 | 0.82 |
| 2.marital | 0.02 | 0.03 | 5.50 | 0.49 |
| 1.insurance | 0.31 | 0.31 | 0.00 | 1.00 |
| 2.insurance | 0.69 | 0.69 | 0.00 | 1.00 |
| 1.radiation | 0.39 | 0.40 | 1.80 | 0.82 |
| 2.radiation | 0.61 | 0.60 | 1.80 | 0.82 |
| 1.surgery#2.seerstage | 0.09 | 0.07 | 9.70 | 0.27 |
| 1.surgery#3.seerstage | 0.01 | 0.00 | 10.60 | 0.18 |
| 1.surgery#4.seerstage | 0.08 | 0.09 | 1.20 | 0.89 |
| 2.surgery#4.seerstage | 0.03 | 0.03 | 0.00 | 1.00 |
| 2.category#2.seerstage | 0.06 | 0.05 | 2.70 | 0.62 |
| 2.category#3.seerstage | 0.12 | 0.12 | 0.00 | 1.00 |
| 3.category#2.seerstage | 0.04 | 0.03 | 1.40 | 0.84 |
| 3.category#3.seerstage | 0.10 | 0.11 | 5.40 | 0.62 |
| 3.category#4.seerstage | 0.25 | 0.25 | 0.80 | 0.93 |
| c.age#c.age | 4976.90 | 4968.50 | 0.60 | 0.92 |
| 1.radiation#2.seerstage | 0.08 | 0.07 | 1.60 | 0.78 |
| 1.radiation#3.seerstage | 0.15 | 0.16 | 4.40 | 0.68 |
| 1.radiation#4.seerstage | 0.13 | 0.14 | 0.90 | 0.91 |
| 2.radiation#2.seerstage | 0.08 | 0.07 | 3.80 | 0.48 |
| 2.radiation#3.seerstage | 0.23 | 0.23 | 0.00 | 1.00 |
| 2.radiation#4.seerstage | 0.17 | 0.18 | 1.70 | 0.84 |
| 2.seerstage#c.age | 11.71 | 10.31 | 4.60 | 0.48 |
| 3.seerstage#c.age | 26.82 | 27.60 | 3.10 | 0.77 |
| 4.seerstage#c.age | 21.08 | 21.64 | 1.90 | 0.82 |
| 1.radiation#2.category | 0.09 | 0.09 | 0.90 | 0.89 |
| 1.radiation#3.category | 0.16 | 0.17 | 1.70 | 0.84 |
| 2.radiation#2.category | 0.15 | 0.14 | 3.10 | 0.67 |
| 2.radiation#3.category | 0.25 | 0.26 | 1.50 | 0.86 |
| 2.category#2.race | 0.05 | 0.04 | 2.90 | 0.71 |
| 2.category#3.race | 0.01 | 0.01 | 2.10 | 0.74 |
| 2.category#4.race | 0.02 | 0.01 | 6.20 | 0.40 |
| 3.category#2.race | 0.09 | 0.09 | 0.00 | 1.00 |
| 3.category#3.race | 0.01 | 0.01 | 0.00 | 1.00 |
| 2.category#1.surgery | 0.08 | 0.07 | 5.30 | 0.48 |
| 3.category#1.surgery | 0.12 | 0.12 | 1.00 | 0.91 |
| 3.category#2.surgery | 0.03 | 0.03 | 0.00 | 1.00 |
| 1.marital#2.race | 0.09 | 0.10 | 3.30 | 0.70 |
| 1.marital#3.race | 0.04 | 0.03 | 3.90 | 0.56 |
| 1.marital#4.race | 0.03 | 0.02 | 8.10 | 0.25 |
| 2.marital#2.race | 0.00 | 0.00 | 4.60 | 0.32 |
| 2.marital#4.race | 0.00 | 0.00 | 5.70 | 0.32 |
| 1.surgery#2.race | 0.03 | 0.03 | 5.20 | 0.51 |
| 1.surgery#3.race | 0.01 | 0.00 | 5.20 | 0.32 |
| 1.surgery#4.race | 0.02 | 0.01 | 10.30 | 0.16 |
| 2.surgery#2.race | 0.00 | 0.00 | 0.00 | 1.00 |
| 2.race#2.seerstage | 0.02 | 0.01 | 4.30 | 0.32 |
| 2.race#3.seerstage | 0.10 | 0.10 | 2.70 | 0.80 |
| 2.race#4.seerstage | 0.06 | 0.07 | 2.90 | 0.76 |
| 3.race#2.seerstage | 0.01 | 0.01 | 1.70 | 0.71 |
| 3.race#3.seerstage | 0.02 | 0.01 | 5.90 | 0.53 |
| 3.race#4.seerstage | 0.01 | 0.01 | 0.00 | 1.00 |
| 4.race#2.seerstage | 0.02 | 0.01 | 7.50 | 0.16 |
| 4.race#3.seerstage | 0.02 | 0.01 | 3.00 | 0.76 |
| 4.race#4.seerstage | 0.00 | 0.00 | 0.00 | 1.00 |
| 1.insurance#1.marital | 0.16 | 0.15 | 2.10 | 0.75 |
| 1.insurance#2.marital | 0.01 | 0.02 | 7.60 | 0.36 |
| 2.insurance#1.marital | 0.46 | 0.46 | 0.00 | 1.00 |
| 2.insurance#2.marital | 0.01 | 0.01 | 0.00 | 1.00 |
| 1.marital#2.category | 0.14 | 0.14 | 0.80 | 0.91 |
| 1.marital#3.category | 0.29 | 0.29 | 0.70 | 0.93 |
| 2.marital#2.category | 0.01 | 0.01 | 0.00 | 1.00 |
| 1.radiation#c.age | 27.80 | 28.28 | 1.40 | 0.85 |
| 2.radiation#c.age | 42.27 | 41.73 | 1.60 | 0.84 |
| 2.race#c.age | 13.55 | 12.90 | 2.70 | 0.75 |
| 3.race#c.age | 3.38 | 2.86 | 3.10 | 0.65 |
| 4.race#c.age | 3.10 | 2.02 | 7.30 | 0.29 |

LAPA: lung cancer with subsequent prostate cancer; bias: absolutely standardized difference; p: t test between matched treated and control group.

Tab S38. balance test group mTPC1 with LAPA and single lung cancer in the matched cohort

| Variable | Mean | |  |  |
| --- | --- | --- | --- | --- |
|  | Treated | Control | bias | P |
| age | 67.49 | 67.66 | 1.80 | 0.73 |
| 1.surgery | 0.75 | 0.76 | 1.20 | 0.83 |
| 2.surgery | 0.01 | 0.01 | 0.00 | 1.00 |
| 2.seerstage | 0.27 | 0.28 | 0.40 | 0.95 |
| 3.seerstage | 0.10 | 0.09 | 3.20 | 0.67 |
| 4.seerstage | 0.17 | 0.17 | 0.00 | 1.00 |
| 2.race | 0.17 | 0.17 | 2.80 | 0.68 |
| 3.race | 0.05 | 0.05 | 0.00 | 1.00 |
| 4.race | 0.04 | 0.05 | 0.90 | 0.88 |
| 1.marital | 0.76 | 0.77 | 3.80 | 0.51 |
| 2.marital | 0.02 | 0.01 | 7.90 | 0.15 |
| 1.insurance | 0.32 | 0.32 | 0.40 | 0.95 |
| 2.insurance | 0.68 | 0.68 | 0.40 | 0.95 |
| 1.radiation | 0.25 | 0.24 | 2.00 | 0.72 |
| 2.radiation | 0.75 | 0.76 | 2.00 | 0.72 |
| 2.category | 0.32 | 0.33 | 2.50 | 0.69 |
| 3.category | 0.30 | 0.30 | 0.40 | 0.95 |
| 2.seerstage#1.surgery | 0.22 | 0.22 | 0.00 | 1.00 |
| 3.seerstage#1.surgery | 0.02 | 0.02 | 3.80 | 0.65 |
| 4.seerstage#1.surgery | 0.11 | 0.11 | 0.70 | 0.92 |
| 4.seerstage#2.surgery | 0.00 | 0.01 | 2.50 | 0.65 |
| 2.category#2.seerstage | 0.11 | 0.11 | 1.80 | 0.77 |
| 2.category#3.seerstage | 0.03 | 0.03 | 1.40 | 0.85 |
| 3.category#2.seerstage | 0.04 | 0.04 | 0.00 | 1.00 |
| 3.category#3.seerstage | 0.01 | 0.01 | 2.00 | 0.76 |
| 3.category#4.seerstage | 0.16 | 0.16 | 0.50 | 0.93 |
| 1.radiation#2.seerstage | 0.09 | 0.09 | 1.10 | 0.83 |
| 1.radiation#3.seerstage | 0.06 | 0.05 | 5.30 | 0.50 |
| 1.radiation#4.seerstage | 0.06 | 0.07 | 1.50 | 0.80 |
| 2.radiation#2.seerstage | 0.18 | 0.19 | 1.50 | 0.81 |
| 2.radiation#3.seerstage | 0.04 | 0.04 | 1.10 | 0.87 |
| 2.radiation#4.seerstage | 0.11 | 0.10 | 1.30 | 0.84 |
| c.age#c.age | 4619.20 | 4642.00 | 1.80 | 0.73 |
| 1.radiation#1.surgery | 0.10 | 0.09 | 1.30 | 0.83 |
| 1.radiation#2.surgery | 0.00 | 0.00 | 2.90 | 0.56 |
| 2.radiation#1.surgery | 0.66 | 0.67 | 2.10 | 0.75 |
| 2.radiation#2.surgery | 0.00 | 0.00 | 3.70 | 0.56 |
| 2.seerstage#c.age | 18.60 | 18.64 | 0.20 | 0.98 |
| 3.seerstage#c.age | 6.40 | 5.95 | 2.80 | 0.71 |
| 4.seerstage#c.age | 11.51 | 11.60 | 0.40 | 0.96 |
| 1.marital#2.race | 0.10 | 0.11 | 5.00 | 0.48 |
| 1.marital#3.race | 0.04 | 0.04 | 0.00 | 1.00 |
| 1.marital#4.race | 0.03 | 0.04 | 1.00 | 0.87 |
| 2.marital#3.race | 0.00 | 0.00 | 4.20 | 0.32 |
| 2.category#1.marital | 0.24 | 0.25 | 0.90 | 0.89 |
| 3.category#1.marital | 0.24 | 0.25 | 2.40 | 0.72 |
| 3.category#2.marital | 0.01 | 0.00 | 6.90 | 0.26 |
| 2.race#c.age | 10.50 | 11.13 | 2.90 | 0.67 |
| 3.race#c.age | 3.37 | 3.43 | 0.30 | 0.95 |
| 4.race#c.age | 3.06 | 3.24 | 1.20 | 0.84 |
| 2.category#2.race | 0.06 | 0.07 | 5.40 | 0.46 |
| 2.category#3.race | 0.01 | 0.01 | 1.40 | 0.78 |
| 2.category#4.race | 0.01 | 0.01 | 0.00 | 1.00 |
| 3.category#2.race | 0.03 | 0.04 | 1.10 | 0.87 |
| 3.category#3.race | 0.02 | 0.02 | 6.40 | 0.37 |
| 3.category#4.race | 0.00 | 0.00 | 5.50 | 0.16 |
| 2.race#1.surgery | 0.11 | 0.11 | 1.50 | 0.84 |
| 3.race#1.surgery | 0.04 | 0.04 | 1.10 | 0.87 |
| 4.race#1.surgery | 0.03 | 0.04 | 3.70 | 0.63 |
| 2.race#2.seerstage | 0.06 | 0.06 | 2.60 | 0.69 |
| 2.race#3.seerstage | 0.02 | 0.02 | 1.80 | 0.82 |
| 2.race#4.seerstage | 0.02 | 0.02 | 1.50 | 0.82 |
| 3.race#2.seerstage | 0.01 | 0.01 | 1.10 | 0.78 |
| 3.race#3.seerstage | 0.01 | 0.01 | 0.00 | 1.00 |
| 3.race#4.seerstage | 0.01 | 0.01 | 2.30 | 0.71 |
| 4.race#2.seerstage | 0.01 | 0.02 | 4.20 | 0.46 |
| 4.race#3.seerstage | 0.01 | 0.00 | 6.30 | 0.32 |
| 4.race#4.seerstage | 0.00 | 0.00 | 0.00 | 1.00 |
| 1.marital#2.seerstage | 0.21 | 0.21 | 0.40 | 0.94 |
| 1.marital#3.seerstage | 0.07 | 0.07 | 1.00 | 0.90 |
| 1.marital#4.seerstage | 0.13 | 0.14 | 2.40 | 0.72 |
| 2.marital#2.seerstage | 0.00 | 0.00 | 0.00 | 1.00 |
| 2.marital#3.seerstage | 0.00 | 0.00 | 7.50 | 0.16 |
| 2.marital#4.seerstage | 0.01 | 0.00 | 7.80 | 0.18 |
| 1.marital#1.surgery | 0.58 | 0.60 | 2.80 | 0.66 |
| 1.marital#2.surgery | 0.01 | 0.01 | 0.00 | 1.00 |
| 2.marital#1.surgery | 0.01 | 0.01 | 6.00 | 0.32 |
| 2.category#c.age | 21.69 | 22.45 | 2.50 | 0.70 |
| 3.category#c.age | 20.34 | 20.32 | 0.10 | 0.99 |
| 1.insurance#2.seerstage | 0.10 | 0.10 | 0.50 | 0.92 |
| 1.insurance#3.seerstage | 0.05 | 0.05 | 1.10 | 0.89 |
| 1.insurance#4.seerstage | 0.01 | 0.01 | 1.50 | 0.74 |
| 2.insurance#2.seerstage | 0.17 | 0.18 | 1.00 | 0.87 |
| 2.insurance#3.seerstage | 0.05 | 0.04 | 3.20 | 0.65 |
| 2.insurance#4.seerstage | 0.16 | 0.16 | 0.50 | 0.93 |

LAPA: lung cancer with subsequent prostate cancer; bias: absolutely standardized difference; p: t test between matched treated and control group.

Tab S39. balance test group mTPC2 with LAPA and single lung cancer in the matched cohort

| Variable | Mean | |  |  |
| --- | --- | --- | --- | --- |
|  | Treated | Control | bias | P |
| age | 62.02 | 61.82 | 2.20 | 0.78 |
| 2.seerstage | 0.26 | 0.23 | 9.20 | 0.36 |
| 3.seerstage | 0.05 | 0.06 | 4.20 | 0.71 |
| 4.seerstage | 0.29 | 0.31 | 4.80 | 0.63 |
| 1.surgery | 0.94 | 0.95 | 3.50 | 0.57 |
| 2.category | 0.37 | 0.33 | 7.90 | 0.36 |
| 3.category | 0.49 | 0.54 | 10.70 | 0.26 |
| 1.marital | 0.81 | 0.85 | 10.20 | 0.20 |
| 2.race | 0.17 | 0.15 | 3.40 | 0.72 |
| 3.race | 0.07 | 0.07 | 2.60 | 0.73 |
| 1.radiation | 0.19 | 0.21 | 4.70 | 0.58 |
| 2.radiation | 0.81 | 0.79 | 4.70 | 0.58 |
| 1.insurance | 0.10 | 0.10 | 0.00 | 1.00 |
| 2.insurance | 0.90 | 0.90 | 0.00 | 1.00 |
| 1.radiation#2.seerstage | 0.12 | 0.12 | 1.40 | 0.89 |
| 1.radiation#3.seerstage | 0.03 | 0.04 | 8.60 | 0.48 |
| 1.radiation#4.seerstage | 0.03 | 0.03 | 2.30 | 0.81 |
| 2.radiation#2.seerstage | 0.14 | 0.10 | 13.40 | 0.18 |
| 2.radiation#3.seerstage | 0.02 | 0.02 | 3.00 | 0.76 |
| 2.radiation#4.seerstage | 0.26 | 0.28 | 4.00 | 0.69 |
| c.age#c.age | 3910.10 | 3883.40 | 2.30 | 0.76 |
| 2.category#2.seerstage | 0.15 | 0.11 | 15.90 | 0.12 |
| 2.category#3.seerstage | 0.04 | 0.04 | 2.50 | 0.82 |
| 3.category#2.seerstage | 0.06 | 0.07 | 8.70 | 0.48 |
| 3.category#3.seerstage | 0.01 | 0.02 | 9.00 | 0.48 |
| 3.category#4.seerstage | 0.29 | 0.31 | 4.80 | 0.63 |
| 2.race#2.seerstage | 0.05 | 0.03 | 13.50 | 0.17 |
| 2.race#3.seerstage | 0.01 | 0.01 | 0.00 | 1.00 |
| 2.race#4.seerstage | 0.05 | 0.07 | 6.50 | 0.58 |
| 3.race#2.seerstage | 0.02 | 0.02 | 0.00 | 1.00 |
| 3.race#4.seerstage | 0.02 | 0.02 | 0.00 | 1.00 |
| 1.surgery#2.seerstage | 0.23 | 0.20 | 8.50 | 0.46 |
| 1.surgery#3.seerstage | 0.03 | 0.03 | 2.90 | 0.81 |
| 1.surgery#4.seerstage | 0.29 | 0.31 | 5.80 | 0.57 |
| 2.category#1.surgery | 0.33 | 0.30 | 5.80 | 0.51 |
| 3.category#1.surgery | 0.48 | 0.54 | 11.70 | 0.22 |
| 2.race#1.surgery | 0.14 | 0.14 | 0.00 | 1.00 |
| 3.race#1.surgery | 0.06 | 0.07 | 2.90 | 0.72 |
| 1.insurance#2.seerstage | 0.04 | 0.05 | 1.90 | 0.83 |
| 1.insurance#3.seerstage | 0.00 | 0.00 | 6.70 | 0.32 |
| 2.insurance#2.seerstage | 0.22 | 0.18 | 11.50 | 0.27 |
| 2.insurance#3.seerstage | 0.05 | 0.06 | 6.50 | 0.57 |
| 2.insurance#4.seerstage | 0.29 | 0.31 | 4.80 | 0.63 |
| 1.marital#2.seerstage | 0.18 | 0.17 | 3.50 | 0.73 |
| 1.marital#3.seerstage | 0.05 | 0.06 | 7.00 | 0.55 |
| 1.marital#4.seerstage | 0.25 | 0.28 | 6.10 | 0.55 |
| 1.radiation#c.age | 11.54 | 12.35 | 3.20 | 0.71 |
| 2.radiation#c.age | 50.47 | 49.47 | 3.70 | 0.66 |
| 2.race#1.marital | 0.12 | 0.12 | 0.00 | 1.00 |
| 3.race#1.marital | 0.05 | 0.07 | 5.90 | 0.45 |
| 2.race#c.age | 10.12 | 9.36 | 3.70 | 0.70 |
| 3.race#c.age | 3.92 | 4.42 | 2.70 | 0.71 |

LAPA: lung cancer with subsequent prostate cancer; bias: absolutely standardized difference; p: t test between matched treated and control group.

Tab S40. balance test LAPA and single prostate cancer in the matched cohort

| Variable | Mean | |  |  |
| --- | --- | --- | --- | --- |
|  | Treated | Control | bias | P |
| age | 70.39 | 70.40 | 0.20 | 0.97 |
| 1.surgery | 0.26 | 0.26 | 0.00 | 1.00 |
| 2.surgery | 0.02 | 0.02 | 0.00 | 1.00 |
| 2.category | 0.27 | 0.27 | 0.00 | 1.00 |
| 3.category | 0.28 | 0.28 | 0.00 | 1.00 |
| 2.race | 0.18 | 0.18 | 0.00 | 1.00 |
| 3.race | 0.04 | 0.04 | 0.00 | 1.00 |
| 4.race | 0.04 | 0.04 | 0.40 | 0.92 |
| 5.race | 0.00 | 0.00 | 0.00 | 1.00 |
| 1.radiation | 0.31 | 0.31 | 0.00 | 1.00 |
| 2.radiation | 0.69 | 0.69 | 0.00 | 1.00 |
| 1.marital | 0.64 | 0.64 | 0.00 | 1.00 |
| 2.marital | 0.11 | 0.11 | 0.00 | 1.00 |
| 1.insurance | 0.34 | 0.35 | 0.20 | 0.97 |
| 2.insurance | 0.65 | 0.65 | 0.00 | 1.00 |
| c.age#c.age | 5018.10 | 5020.30 | 0.20 | 0.96 |
| 2.category#c.age | 18.84 | 18.84 | 0.00 | 1.00 |
| 3.category#c.age | 19.84 | 19.84 | 0.00 | 1.00 |
| 1.surgery#c.age | 18.40 | 18.41 | 0.00 | 0.99 |
| 2.surgery#c.age | 1.71 | 1.71 | 0.00 | 1.00 |
| 2.category#1.surgery | 0.07 | 0.07 | 0.00 | 1.00 |
| 2.category#2.surgery | 0.01 | 0.01 | 0.00 | 1.00 |
| 3.category#1.surgery | 0.10 | 0.10 | 0.00 | 1.00 |
| 3.category#2.surgery | 0.01 | 0.01 | 0.00 | 1.00 |
| 1.radiation#1.surgery | 0.04 | 0.04 | 0.00 | 1.00 |
| 1.radiation#2.surgery | 0.00 | 0.00 | 0.00 | 1.00 |
| 2.radiation#1.surgery | 0.23 | 0.23 | 0.00 | 1.00 |
| 2.radiation#2.surgery | 0.02 | 0.02 | 0.00 | 1.00 |
| 1.marital#c.age | 45.14 | 45.14 | 0.00 | 1.00 |
| 2.marital#c.age | 8.15 | 8.16 | 0.10 | 0.99 |
| 1.marital#2.category | 0.19 | 0.19 | 0.00 | 1.00 |
| 1.marital#3.category | 0.20 | 0.20 | 0.00 | 1.00 |
| 2.marital#2.category | 0.02 | 0.02 | 0.00 | 1.00 |
| 2.marital#3.category | 0.01 | 0.01 | 0.00 | 1.00 |
| 1.radiation#2.category | 0.10 | 0.10 | 0.00 | 1.00 |
| 1.radiation#3.category | 0.06 | 0.06 | 0.00 | 1.00 |
| 2.radiation#2.category | 0.16 | 0.16 | 0.00 | 1.00 |
| 2.radiation#3.category | 0.22 | 0.22 | 0.00 | 1.00 |
| 1.insurance#1.marital | 0.22 | 0.22 | 0.00 | 1.00 |
| 1.insurance#2.marital | 0.03 | 0.03 | 0.50 | 0.90 |
| 2.insurance#1.marital | 0.42 | 0.42 | 0.00 | 1.00 |
| 2.insurance#2.marital | 0.09 | 0.09 | 0.00 | 1.00 |
| 1.radiation#2.race | 0.05 | 0.05 | 0.00 | 1.00 |
| 1.radiation#3.race | 0.01 | 0.01 | 0.00 | 1.00 |
| 1.radiation#4.race | 0.01 | 0.01 | 0.00 | 1.00 |
| 1.radiation#5.race | 0.00 | 0.00 | 0.00 | 1.00 |
| 2.radiation#2.race | 0.13 | 0.13 | 0.00 | 1.00 |
| 2.radiation#3.race | 0.03 | 0.03 | 0.00 | 1.00 |
| 2.radiation#4.race | 0.03 | 0.03 | 0.40 | 0.90 |
| 2.radiation#5.race | 0.00 | 0.00 | 0.00 | 1.00 |
| 2.race#2.category | 0.05 | 0.05 | 0.00 | 1.00 |
| 2.race#3.category | 0.04 | 0.04 | 0.00 | 1.00 |
| 3.race#2.category | 0.01 | 0.01 | 0.00 | 1.00 |
| 3.race#3.category | 0.01 | 0.01 | 0.00 | 1.00 |
| 4.race#2.category | 0.01 | 0.01 | 0.00 | 1.00 |
| 4.race#3.category | 0.00 | 0.00 | 0.00 | 1.00 |
| 5.race#3.category | 0.00 | 0.00 | 0.00 | 1.00 |
| 2.race#1.surgery | 0.04 | 0.04 | 0.00 | 1.00 |
| 2.race#2.surgery | 0.00 | 0.00 | 0.00 | 1.00 |
| 3.race#1.surgery | 0.01 | 0.01 | 0.00 | 1.00 |
| 3.race#2.surgery | 0.00 | 0.00 | 0.00 | 1.00 |
| 4.race#1.surgery | 0.01 | 0.01 | 0.60 | 0.81 |
| 5.race#1.surgery | 0.00 | 0.00 | 0.00 | 1.00 |
| 2.race#c.age | 11.91 | 11.91 | 0.00 | 1.00 |
| 3.race#c.age | 3.16 | 3.16 | 0.00 | 1.00 |
| 4.race#c.age | 2.91 | 2.85 | 0.40 | 0.92 |
| 5.race#c.age | 0.11 | 0.11 | 0.00 | 1.00 |
| 1.marital#2.race | 0.09 | 0.09 | 0.00 | 1.00 |
| 1.marital#3.race | 0.03 | 0.03 | 0.00 | 1.00 |
| 1.marital#4.race | 0.02 | 0.02 | 0.00 | 1.00 |
| 1.marital#5.race | 0.00 | 0.00 | 0.00 | 1.00 |
| 2.marital#2.race | 0.02 | 0.02 | 0.00 | 1.00 |
| 2.marital#3.race | 0.01 | 0.01 | 0.00 | 1.00 |
| 2.marital#4.race | 0.01 | 0.01 | 0.80 | 0.84 |
| 1.marital#1.radiation | 0.22 | 0.22 | 0.00 | 1.00 |
| 1.marital#2.radiation | 0.42 | 0.42 | 0.00 | 1.00 |
| 2.marital#1.radiation | 0.02 | 0.02 | 0.00 | 1.00 |
| 2.marital#2.radiation | 0.09 | 0.09 | 0.00 | 1.00 |
| 1.marital#1.surgery | 0.19 | 0.19 | 0.00 | 1.00 |
| 1.marital#2.surgery | 0.01 | 0.01 | 0.00 | 1.00 |
| 2.marital#1.surgery | 0.01 | 0.01 | 0.00 | 1.00 |
| 2.marital#2.surgery | 0.01 | 0.01 | 0.00 | 1.00 |
| 1.insurance#1.radiation | 0.12 | 0.12 | 0.00 | 1.00 |
| 1.insurance#2.radiation | 0.22 | 0.22 | 0.20 | 0.96 |
| 2.insurance#1.radiation | 0.18 | 0.18 | 0.00 | 1.00 |
| 2.insurance#2.radiation | 0.47 | 0.47 | 0.00 | 1.00 |

LAPA: lung cancer with subsequent prostate cancer; bias: absolutely standardized difference; p: t test between matched treated and control group.

Tab S41. balance test group sTPC with LAPA and single prostate cancer in the matched cohort

| Variable | Mean | |  |  |
| --- | --- | --- | --- | --- |
|  | Treated | Control | bias | P |
| age | 70.18 | 70.18 | 0.00 | 1.00 |
| 2.category | 0.24 | 0.24 | 0.00 | 1.00 |
| 3.category | 0.40 | 0.40 | 0.00 | 1.00 |
| 1.surgery | 0.22 | 0.22 | 0.00 | 1.00 |
| 2.surgery | 0.04 | 0.04 | 0.00 | 1.00 |
| 1.radiation | 0.17 | 0.17 | 0.00 | 1.00 |
| 2.radiation | 0.83 | 0.83 | 0.00 | 1.00 |
| 2.race | 0.20 | 0.20 | 0.00 | 1.00 |
| 3.race | 0.05 | 0.05 | 0.00 | 1.00 |
| 4.race | 0.04 | 0.04 | 0.00 | 1.00 |
| 1.marital | 0.57 | 0.57 | 0.00 | 1.00 |
| 2.marital | 0.10 | 0.10 | 0.00 | 1.00 |
| 1.insurance | 0.25 | 0.25 | 0.00 | 1.00 |
| 2.insurance | 0.75 | 0.75 | 0.00 | 1.00 |
| 2.category#c.age | 17.26 | 17.26 | 0.00 | 1.00 |
| 3.category#c.age | 28.15 | 28.15 | 0.00 | 1.00 |
| 1.surgery#c.age | 15.83 | 15.83 | 0.00 | 1.00 |
| 2.surgery#c.age | 2.90 | 2.90 | 0.00 | 1.00 |
| c.age#c.age | 4992.00 | 4992.00 | 0.00 | 1.00 |
| 1.surgery#2.category | 0.06 | 0.06 | 0.00 | 1.00 |
| 1.surgery#3.category | 0.11 | 0.11 | 0.00 | 1.00 |
| 2.surgery#2.category | 0.01 | 0.01 | 0.00 | 1.00 |
| 2.surgery#3.category | 0.01 | 0.01 | 0.00 | 1.00 |
| 1.radiation#1.surgery | 0.03 | 0.03 | 0.00 | 1.00 |
| 1.radiation#2.surgery | 0.01 | 0.01 | 0.00 | 1.00 |
| 2.radiation#1.surgery | 0.19 | 0.19 | 0.00 | 1.00 |
| 2.radiation#2.surgery | 0.03 | 0.03 | 0.00 | 1.00 |
| 1.marital#2.category | 0.14 | 0.14 | 0.00 | 1.00 |
| 1.marital#3.category | 0.27 | 0.27 | 0.00 | 1.00 |
| 2.marital#2.category | 0.02 | 0.02 | 0.00 | 1.00 |
| 2.marital#3.category | 0.00 | 0.00 | 0.00 | 1.00 |
| 2.race#2.category | 0.05 | 0.05 | 0.00 | 1.00 |
| 2.race#3.category | 0.08 | 0.08 | 0.00 | 1.00 |
| 3.race#2.category | 0.01 | 0.01 | 0.00 | 1.00 |
| 3.race#3.category | 0.01 | 0.01 | 0.00 | 1.00 |
| 4.race#2.category | 0.02 | 0.02 | 0.00 | 1.00 |
| 1.marital#2.race | 0.09 | 0.09 | 0.00 | 1.00 |
| 1.marital#3.race | 0.04 | 0.04 | 0.00 | 1.00 |
| 1.marital#4.race | 0.03 | 0.03 | 0.00 | 1.00 |
| 2.marital#2.race | 0.01 | 0.01 | 0.00 | 1.00 |
| 2.marital#3.race | 0.01 | 0.01 | 0.00 | 1.00 |
| 2.marital#4.race | 0.01 | 0.01 | 0.00 | 1.00 |
| 2.race#1.radiation | 0.02 | 0.02 | 0.00 | 1.00 |
| 2.race#2.radiation | 0.18 | 0.18 | 0.00 | 1.00 |
| 3.race#1.radiation | 0.01 | 0.01 | 0.00 | 1.00 |
| 3.race#2.radiation | 0.04 | 0.04 | 0.00 | 1.00 |
| 4.race#1.radiation | 0.00 | 0.00 | 0.00 | 1.00 |
| 4.race#2.radiation | 0.04 | 0.04 | 0.00 | 1.00 |
| 2.race#1.surgery | 0.03 | 0.03 | 0.00 | 1.00 |
| 2.race#2.surgery | 0.01 | 0.01 | 0.00 | 1.00 |
| 3.race#1.surgery | 0.01 | 0.01 | 0.00 | 1.00 |
| 4.race#1.surgery | 0.01 | 0.01 | 0.00 | 1.00 |
| 1.marital#c.age | 40.13 | 40.13 | 0.00 | 1.00 |
| 2.marital#c.age | 7.05 | 7.05 | 0.00 | 1.00 |
| 1.insurance#1.marital | 0.13 | 0.13 | 0.00 | 1.00 |
| 1.insurance#2.marital | 0.02 | 0.02 | 0.00 | 1.00 |
| 2.insurance#1.marital | 0.44 | 0.44 | 0.00 | 1.00 |
| 2.insurance#2.marital | 0.08 | 0.08 | 0.00 | 1.00 |
| 1.insurance#c.age | 17.53 | 17.53 | 0.00 | 1.00 |
| 2.insurance#c.age | 52.30 | 52.30 | 0.00 | 1.00 |
| 1.radiation#c.age | 11.28 | 11.28 | 0.00 | 1.00 |
| 2.radiation#c.age | 58.47 | 58.47 | 0.00 | 1.00 |
| 1.insurance#2.race | 0.06 | 0.06 | 0.00 | 1.00 |
| 1.insurance#3.race | 0.01 | 0.01 | 0.00 | 1.00 |
| 1.insurance#4.race | 0.01 | 0.01 | 0.00 | 1.00 |
| 2.insurance#2.race | 0.14 | 0.14 | 0.00 | 1.00 |
| 2.insurance#3.race | 0.03 | 0.03 | 0.00 | 1.00 |
| 2.insurance#4.race | 0.03 | 0.03 | 0.00 | 1.00 |
| 2.race#c.age | 13.73 | 13.73 | 0.00 | 1.00 |
| 3.race#c.age | 3.34 | 3.34 | 0.00 | 1.00 |
| 4.race#c.age | 3.05 | 3.05 | 0.00 | 1.00 |

LAPA: lung cancer with subsequent prostate cancer; bias: absolutely standardized difference; p: t test between matched treated and control group.

Tab S42. balance test group mTPC1 with LAPA and single prostate cancer in the matched cohort

| Variable | Mean | |  |  |
| --- | --- | --- | --- | --- |
|  | Treated | Control | bias | P |
| age | 69.78 | 69.78 | 0.10 | 0.99 |
| 1.surgery | 0.25 | 0.25 | 0.00 | 1.00 |
| 2.surgery | 0.02 | 0.02 | 0.00 | 1.00 |
| 2.category | 0.27 | 0.27 | 0.00 | 1.00 |
| 3.category | 0.26 | 0.26 | 0.00 | 1.00 |
| 2.race | 0.16 | 0.16 | 0.00 | 1.00 |
| 3.race | 0.05 | 0.05 | 0.00 | 1.00 |
| 4.race | 0.04 | 0.04 | 0.00 | 1.00 |
| 5.race | 0.00 | 0.00 | 0.00 | 1.00 |
| 1.radiation | 0.35 | 0.35 | 0.00 | 1.00 |
| 2.radiation | 0.64 | 0.64 | 0.00 | 1.00 |
| 1.marital | 0.67 | 0.67 | 0.00 | 1.00 |
| 2.marital | 0.12 | 0.12 | 0.00 | 1.00 |
| 1.insurance | 0.37 | 0.37 | 0.00 | 1.00 |
| 2.insurance | 0.63 | 0.63 | 0.00 | 1.00 |
| c.age#c.age | 4932.00 | 4931.20 | 0.10 | 0.99 |
| 2.category#c.age | 18.41 | 18.41 | 0.00 | 1.00 |
| 3.category#c.age | 18.00 | 18.00 | 0.00 | 1.00 |
| 1.surgery#c.age | 17.63 | 17.63 | 0.00 | 1.00 |
| 2.surgery#c.age | 1.43 | 1.43 | 0.00 | 1.00 |
| 2.race#2.category | 0.05 | 0.05 | 0.00 | 1.00 |
| 2.race#3.category | 0.03 | 0.03 | 0.00 | 1.00 |
| 3.race#2.category | 0.02 | 0.02 | 0.00 | 1.00 |
| 3.race#3.category | 0.01 | 0.01 | 0.00 | 1.00 |
| 4.race#2.category | 0.01 | 0.01 | 0.00 | 1.00 |
| 5.race#3.category | 0.00 | 0.00 | 0.00 | 1.00 |
| 2.race#c.age | 10.64 | 10.64 | 0.00 | 1.00 |
| 3.race#c.age | 3.53 | 3.53 | 0.00 | 1.00 |
| 4.race#c.age | 3.09 | 3.09 | 0.00 | 1.00 |
| 5.race#c.age | 0.23 | 0.23 | 0.00 | 1.00 |
| 1.radiation#2.race | 0.06 | 0.06 | 0.00 | 1.00 |
| 1.radiation#3.race | 0.02 | 0.02 | 0.00 | 1.00 |
| 1.radiation#4.race | 0.01 | 0.01 | 0.00 | 1.00 |
| 1.radiation#5.race | 0.00 | 0.00 | 0.00 | 1.00 |
| 2.radiation#2.race | 0.10 | 0.10 | 0.00 | 1.00 |
| 2.radiation#3.race | 0.03 | 0.03 | 0.00 | 1.00 |
| 2.radiation#4.race | 0.03 | 0.03 | 0.00 | 1.00 |
| 2.radiation#5.race | 0.00 | 0.00 | 0.00 | 1.00 |
| 2.category#1.surgery | 0.07 | 0.07 | 0.00 | 1.00 |
| 2.category#2.surgery | 0.01 | 0.01 | 0.00 | 1.00 |
| 3.category#1.surgery | 0.10 | 0.10 | 0.00 | 1.00 |
| 3.category#2.surgery | 0.00 | 0.00 | 0.00 | 1.00 |
| 1.insurance#1.surgery | 0.09 | 0.09 | 0.00 | 1.00 |
| 1.insurance#2.surgery | 0.00 | 0.00 | 0.00 | 1.00 |
| 2.insurance#1.surgery | 0.17 | 0.17 | 0.00 | 1.00 |
| 2.insurance#2.surgery | 0.02 | 0.02 | 0.00 | 1.00 |
| 1.marital#c.age | 47.21 | 47.21 | 0.00 | 1.00 |
| 2.marital#c.age | 8.54 | 8.53 | 0.00 | 1.00 |
| 1.radiation#2.category | 0.11 | 0.11 | 0.00 | 1.00 |
| 1.radiation#3.category | 0.06 | 0.06 | 0.00 | 1.00 |
| 2.radiation#2.category | 0.15 | 0.15 | 0.00 | 1.00 |
| 2.radiation#3.category | 0.20 | 0.20 | 0.00 | 1.00 |
| 1.radiation#1.surgery | 0.03 | 0.03 | 0.00 | 1.00 |
| 1.radiation#2.surgery | 0.00 | 0.00 | 0.00 | 1.00 |
| 2.radiation#1.surgery | 0.22 | 0.22 | 0.00 | 1.00 |
| 2.radiation#2.surgery | 0.02 | 0.02 | 0.00 | 1.00 |
| 1.insurance#1.marital | 0.25 | 0.25 | 0.00 | 1.00 |
| 1.insurance#2.marital | 0.03 | 0.03 | 0.00 | 1.00 |
| 2.insurance#1.marital | 0.43 | 0.43 | 0.00 | 1.00 |
| 2.insurance#2.marital | 0.09 | 0.09 | 0.00 | 1.00 |
| 1.insurance#2.race | 0.07 | 0.07 | 0.00 | 1.00 |
| 1.insurance#3.race | 0.02 | 0.02 | 0.00 | 1.00 |
| 1.insurance#4.race | 0.02 | 0.02 | 0.00 | 1.00 |
| 1.insurance#5.race | 0.00 | 0.00 | 0.00 | 1.00 |
| 2.insurance#2.race | 0.09 | 0.09 | 0.00 | 1.00 |
| 2.insurance#3.race | 0.03 | 0.03 | 0.00 | 1.00 |
| 2.insurance#4.race | 0.02 | 0.02 | 0.00 | 1.00 |
| 2.insurance#5.race | 0.00 | 0.00 | 0.00 | 1.00 |
| 1.marital#2.race | 0.09 | 0.09 | 0.00 | 1.00 |
| 1.marital#3.race | 0.03 | 0.03 | 0.00 | 1.00 |
| 1.marital#4.race | 0.02 | 0.02 | 0.00 | 1.00 |
| 1.marital#5.race | 0.00 | 0.00 | 0.00 | 1.00 |
| 2.marital#2.race | 0.02 | 0.02 | 0.00 | 1.00 |
| 2.marital#3.race | 0.01 | 0.01 | 0.00 | 1.00 |
| 2.marital#4.race | 0.01 | 0.01 | 0.00 | 1.00 |
| 1.marital#2.category | 0.19 | 0.19 | 0.00 | 1.00 |
| 1.marital#3.category | 0.20 | 0.20 | 0.00 | 1.00 |
| 2.marital#2.category | 0.02 | 0.02 | 0.00 | 1.00 |
| 2.marital#3.category | 0.01 | 0.01 | 0.00 | 1.00 |
| 2.race#1.surgery | 0.03 | 0.03 | 0.00 | 1.00 |
| 2.race#2.surgery | 0.00 | 0.00 | 0.00 | 1.00 |
| 3.race#1.surgery | 0.01 | 0.01 | 0.00 | 1.00 |
| 3.race#2.surgery | 0.00 | 0.00 | 0.00 | 1.00 |
| 4.race#1.surgery | 0.01 | 0.01 | 0.00 | 1.00 |
| 5.race#1.surgery | 0.00 | 0.00 | 0.00 | 1.00 |

LAPA: lung cancer with subsequent prostate cancer; bias: absolutely standardized difference; p: t test between matched treated and control group.

Tab S43. balance test group mTPC2 with LAPA and single prostate cancer in the matched cohort

| Variable | Mean | |  |  |
| --- | --- | --- | --- | --- |
|  | Treated | Control | bias | P |
| age | 71.87 | 71.87 | 0.00 | 1.00 |
| 2.race | 0.17 | 0.17 | 0.00 | 1.00 |
| 3.race | 0.03 | 0.03 | 0.00 | 1.00 |
| 4.race | 0.03 | 0.03 | 0.00 | 1.00 |
| 1.insurance | 0.42 | 0.42 | 0.00 | 1.00 |
| 2.insurance | 0.58 | 0.58 | 0.00 | 1.00 |
| 1.radiation | 0.39 | 0.39 | 0.00 | 1.00 |
| 2.radiation | 0.61 | 0.61 | 0.00 | 1.00 |
| 2.category | 0.30 | 0.30 | 0.00 | 1.00 |
| 3.category | 0.18 | 0.18 | 0.00 | 1.00 |
| 1.surgery | 0.33 | 0.33 | 0.00 | 1.00 |
| 2.surgery | 0.01 | 0.01 | 0.00 | 1.00 |
| c.age#c.age | 5224.50 | 5224.50 | 0.00 | 1.00 |
| 2.category#c.age | 21.77 | 21.77 | 0.00 | 1.00 |
| 3.category#c.age | 12.66 | 12.66 | 0.00 | 1.00 |
| 2.category#2.race | 0.06 | 0.06 | 0.00 | 1.00 |
| 2.category#3.race | 0.00 | 0.00 | 0.00 | 1.00 |
| 3.category#2.race | 0.02 | 0.02 | 0.00 | 1.00 |
| 3.category#3.race | 0.01 | 0.01 | 0.00 | 1.00 |
| 3.category#4.race | 0.01 | 0.01 | 0.00 | 1.00 |
| 1.surgery#2.race | 0.07 | 0.07 | 0.00 | 1.00 |
| 1.surgery#3.race | 0.01 | 0.01 | 0.00 | 1.00 |
| 1.surgery#4.race | 0.01 | 0.01 | 0.00 | 1.00 |
| 1.surgery#c.age | 23.29 | 23.29 | 0.00 | 1.00 |
| 2.surgery#c.age | 0.72 | 0.72 | 0.00 | 1.00 |
| 1.insurance#2.race | 0.06 | 0.06 | 0.00 | 1.00 |
| 1.insurance#3.race | 0.01 | 0.01 | 0.00 | 1.00 |
| 1.insurance#4.race | 0.02 | 0.02 | 0.00 | 1.00 |
| 2.insurance#2.race | 0.11 | 0.11 | 0.00 | 1.00 |
| 2.insurance#3.race | 0.02 | 0.02 | 0.00 | 1.00 |
| 2.insurance#4.race | 0.01 | 0.01 | 0.00 | 1.00 |
| 2.category#1.radiation | 0.14 | 0.14 | 0.00 | 1.00 |
| 2.category#2.radiation | 0.16 | 0.16 | 0.00 | 1.00 |
| 3.category#1.radiation | 0.07 | 0.07 | 0.00 | 1.00 |
| 3.category#2.radiation | 0.11 | 0.11 | 0.00 | 1.00 |
| 1.insurance#c.age | 29.64 | 29.64 | 0.00 | 1.00 |
| 2.insurance#c.age | 42.24 | 42.24 | 0.00 | 1.00 |

LAPA: lung cancer with subsequent prostate cancer; bias: absolutely standardized difference; p: t test between matched treated and control group.

Tab S44. balance test between PALA and single lung cancer in the matched cohort

| Variable | Mean | |  |  |
| --- | --- | --- | --- | --- |
|  | Treated | Control | bias | P |
| age | 74.28 | 74.24 | 0.40 | 0.75 |
| 2.seerstage | 0.19 | 0.19 | 0.10 | 0.93 |
| 3.seerstage | 0.52 | 0.52 | 0.60 | 0.80 |
| 4.seerstage | 0.11 | 0.11 | 0.00 | 0.98 |
| 2.category | 0.26 | 0.25 | 1.30 | 0.41 |
| 3.category | 0.09 | 0.09 | 0.10 | 0.93 |
| 1.surgery | 0.25 | 0.24 | 1.20 | 0.46 |
| 2.surgery | 0.01 | 0.01 | 2.10 | 0.08 |
| 2.race | 0.16 | 0.16 | 0.60 | 0.75 |
| 3.race | 0.05 | 0.05 | 0.60 | 0.67 |
| 4.race | 0.05 | 0.05 | 0.80 | 0.64 |
| 5.race | 0.00 | 0.00 | 2.10 | 0.13 |
| 1.radiation | 0.36 | 0.35 | 3.30 | 0.05 |
| 2.radiation | 0.64 | 0.65 | 3.30 | 0.04 |
| 1.marital | 0.68 | 0.70 | 3.00 | 0.07 |
| 2.marital | 0.04 | 0.03 | 4.60 | 0.00 |
| 1.surgery#2.seerstage | 0.10 | 0.10 | 0.20 | 0.93 |
| 1.surgery#3.seerstage | 0.02 | 0.02 | 6.30 | 0.00 |
| 1.surgery#4.seerstage | 0.02 | 0.02 | 0.20 | 0.91 |
| 2.surgery#2.seerstage | 0.00 | 0.00 | 0.70 | 0.56 |
| 2.surgery#3.seerstage | 0.00 | 0.00 | 2.90 | 0.08 |
| 2.surgery#4.seerstage | 0.00 | 0.00 | 0.40 | 0.67 |
| 2.category#2.seerstage | 0.06 | 0.07 | 0.80 | 0.54 |
| 2.category#3.seerstage | 0.14 | 0.14 | 3.60 | 0.11 |
| 3.category#2.seerstage | 0.02 | 0.02 | 0.80 | 0.48 |
| 3.category#3.seerstage | 0.03 | 0.03 | 1.20 | 0.57 |
| 3.category#4.seerstage | 0.03 | 0.03 | 0.10 | 0.96 |
| c.age#c.age | 5578.50 | 5571.50 | 0.50 | 0.71 |
| 1.radiation#1.surgery | 0.04 | 0.03 | 3.40 | 0.02 |
| 1.radiation#2.surgery | 0.00 | 0.00 | 1.40 | 0.12 |
| 2.radiation#1.surgery | 0.20 | 0.21 | 0.60 | 0.74 |
| 2.radiation#2.surgery | 0.01 | 0.01 | 1.60 | 0.27 |
| 2.seerstage#c.age | 14.25 | 14.21 | 0.10 | 0.94 |
| 3.seerstage#c.age | 38.80 | 38.61 | 0.70 | 0.77 |
| 4.seerstage#c.age | 8.21 | 8.19 | 0.10 | 0.96 |
| 1.marital#2.seerstage | 0.14 | 0.14 | 0.60 | 0.66 |
| 1.marital#3.seerstage | 0.36 | 0.37 | 2.70 | 0.24 |
| 1.marital#4.seerstage | 0.08 | 0.08 | 0.10 | 0.93 |
| 2.marital#2.seerstage | 0.01 | 0.00 | 1.60 | 0.14 |
| 2.marital#3.seerstage | 0.02 | 0.01 | 6.30 | 0.00 |
| 2.marital#4.seerstage | 0.00 | 0.00 | 1.60 | 0.29 |
| 1.radiation#2.category | 0.09 | 0.09 | 2.40 | 0.12 |
| 1.radiation#3.category | 0.04 | 0.04 | 0.30 | 0.82 |
| 2.radiation#2.category | 0.16 | 0.16 | 0.40 | 0.79 |
| 2.radiation#3.category | 0.06 | 0.06 | 0.40 | 0.80 |
| 1.radiation#2.seerstage | 0.08 | 0.08 | 0.20 | 0.88 |
| 1.radiation#3.seerstage | 0.20 | 0.18 | 5.40 | 0.02 |
| 1.radiation#4.seerstage | 0.04 | 0.04 | 0.50 | 0.73 |
| 2.radiation#2.seerstage | 0.11 | 0.11 | 0.10 | 0.96 |
| 2.radiation#3.seerstage | 0.32 | 0.34 | 4.00 | 0.08 |
| 2.radiation#4.seerstage | 0.07 | 0.07 | 0.40 | 0.82 |
| 2.race#c.age | 11.39 | 11.15 | 1.00 | 0.57 |
| 3.race#c.age | 3.87 | 3.74 | 0.70 | 0.66 |
| 4.race#c.age | 3.70 | 3.58 | 0.80 | 0.66 |
| 5.race#c.age | 0.23 | 0.15 | 2.00 | 0.20 |
| 1.surgery#2.category | 0.07 | 0.07 | 0.40 | 0.82 |
| 1.surgery#3.category | 0.03 | 0.03 | 0.50 | 0.72 |
| 2.surgery#2.category | 0.00 | 0.00 | 1.40 | 0.29 |
| 2.surgery#3.category | 0.00 | 0.00 | 0.50 | 0.63 |
| 1.radiation#c.age | 26.81 | 25.64 | 3.40 | 0.05 |
| 2.radiation#c.age | 47.45 | 48.60 | 3.20 | 0.06 |
| 2.race#2.seerstage | 0.03 | 0.03 | 0.80 | 0.54 |
| 2.race#3.seerstage | 0.08 | 0.08 | 1.00 | 0.65 |
| 2.race#4.seerstage | 0.02 | 0.02 | 0.00 | 1.00 |
| 3.race#2.seerstage | 0.01 | 0.01 | 0.50 | 0.58 |
| 3.race#3.seerstage | 0.03 | 0.03 | 1.40 | 0.54 |
| 3.race#4.seerstage | 0.01 | 0.01 | 0.80 | 0.58 |
| 4.race#2.seerstage | 0.01 | 0.01 | 0.00 | 1.00 |
| 4.race#3.seerstage | 0.03 | 0.03 | 0.20 | 0.92 |
| 4.race#4.seerstage | 0.01 | 0.00 | 1.60 | 0.29 |
| 5.race#2.seerstage | 0.00 | 0.00 | 0.70 | 0.41 |
| 5.race#3.seerstage | 0.00 | 0.00 | 2.60 | 0.18 |
| 2.race#2.category | 0.04 | 0.03 | 2.80 | 0.09 |
| 2.race#3.category | 0.01 | 0.01 | 0.50 | 0.68 |
| 3.race#2.category | 0.01 | 0.01 | 2.10 | 0.12 |
| 3.race#3.category | 0.00 | 0.00 | 0.50 | 0.64 |
| 4.race#2.category | 0.01 | 0.01 | 1.70 | 0.26 |
| 4.race#3.category | 0.00 | 0.00 | 2.40 | 0.01 |
| 5.race#2.category | 0.00 | 0.00 | 0.40 | 0.74 |
| 5.race#3.category | 0.00 | 0.00 | 0.80 | 0.32 |
| 1.marital#2.race | 0.09 | 0.09 | 2.80 | 0.13 |
| 1.marital#3.race | 0.04 | 0.04 | 0.40 | 0.80 |
| 1.marital#4.race | 0.03 | 0.03 | 0.30 | 0.85 |
| 1.marital#5.race | 0.00 | 0.00 | 1.80 | 0.26 |
| 2.marital#2.race | 0.01 | 0.01 | 3.40 | 0.05 |
| 2.marital#3.race | 0.00 | 0.00 | 1.30 | 0.21 |
| 2.marital#4.race | 0.00 | 0.00 | 0.60 | 0.74 |
| 2.marital#5.race | 0.00 | 0.00 | 0.60 | 0.56 |
| 1.radiation#2.race | 0.05 | 0.05 | 2.40 | 0.12 |
| 1.radiation#3.race | 0.02 | 0.02 | 0.30 | 0.85 |
| 1.radiation#4.race | 0.01 | 0.01 | 1.50 | 0.29 |
| 1.radiation#5.race | 0.00 | 0.00 | 3.30 | 0.01 |
| 2.radiation#2.race | 0.11 | 0.11 | 1.30 | 0.47 |
| 2.radiation#3.race | 0.03 | 0.03 | 0.60 | 0.71 |
| 2.radiation#4.race | 0.04 | 0.04 | 0.20 | 0.93 |
| 2.radiation#5.race | 0.00 | 0.00 | 0.00 | 1.00 |
| 2.category#c.age | 18.92 | 18.43 | 1.60 | 0.36 |
| 3.category#c.age | 6.80 | 6.74 | 0.20 | 0.87 |
| 1.marital#c.age | 50.72 | 51.81 | 3.20 | 0.06 |
| 2.marital#c.age | 2.70 | 2.04 | 5.00 | 0.00 |
| 1.surgery#c.age | 17.74 | 17.36 | 1.30 | 0.47 |
| 2.surgery#c.age | 0.65 | 0.46 | 2.40 | 0.08 |
| 1.marital#1.surgery | 0.18 | 0.17 | 0.60 | 0.71 |
| 1.marital#2.surgery | 0.01 | 0.00 | 1.20 | 0.34 |
| 2.marital#1.surgery | 0.01 | 0.00 | 3.30 | 0.03 |
| 2.marital#2.surgery | 0.00 | 0.00 | 2.80 | 0.01 |
| 2.race#1.surgery | 0.04 | 0.03 | 1.70 | 0.33 |
| 2.race#2.surgery | 0.00 | 0.00 | 2.00 | 0.10 |
| 3.race#1.surgery | 0.01 | 0.01 | 0.40 | 0.81 |
| 3.race#2.surgery | 0.00 | 0.00 | 1.30 | 0.32 |
| 4.race#1.surgery | 0.01 | 0.01 | 0.70 | 0.67 |
| 4.race#2.surgery | 0.00 | 0.00 | 1.70 | 0.26 |
| 5.race#1.surgery | 0.00 | 0.00 | 0.50 | 0.74 |
| 1.marital#1.radiation | 0.26 | 0.25 | 0.90 | 0.58 |
| 1.marital#2.radiation | 0.43 | 0.44 | 3.80 | 0.03 |
| 2.marital#1.radiation | 0.01 | 0.01 | 2.60 | 0.07 |
| 2.marital#2.radiation | 0.03 | 0.02 | 3.70 | 0.02 |

PALA: prostate cancer with subsequent lung cancer; bias: absolutely standardized difference; p: t test between matched treated and control group.

Tab S45. balance test between group sTPC with PALA and single lung cancer in the matched cohort

| Variable | Mean | |  |  |
| --- | --- | --- | --- | --- |
|  | Treated | Control | bias | P |
| age | 69.95 | 69.66 | 3.10 | 0.48 |
| 2.seerstage | 0.25 | 0.25 | 0.30 | 0.95 |
| 3.seerstage | 0.42 | 0.42 | 0.40 | 0.96 |
| 4.seerstage | 0.14 | 0.14 | 0.40 | 0.94 |
| 1.surgery | 0.36 | 0.35 | 3.30 | 0.57 |
| 2.surgery | 0.02 | 0.02 | 0.00 | 1.00 |
| 2.race | 0.18 | 0.18 | 0.40 | 0.94 |
| 3.race | 0.05 | 0.05 | 0.60 | 0.90 |
| 4.race | 0.05 | 0.04 | 4.00 | 0.44 |
| 1.marital | 0.71 | 0.72 | 2.30 | 0.67 |
| 2.marital | 0.04 | 0.03 | 7.20 | 0.17 |
| 2.category | 0.31 | 0.32 | 2.00 | 0.72 |
| 3.category | 0.22 | 0.24 | 2.90 | 0.60 |
| 1.radiation | 0.37 | 0.37 | 0.00 | 1.00 |
| 2.radiation | 0.63 | 0.63 | 0.00 | 1.00 |
| 1.surgery#2.seerstage | 0.14 | 0.14 | 1.10 | 0.88 |
| 1.surgery#3.seerstage | 0.03 | 0.03 | 4.70 | 0.51 |
| 1.surgery#4.seerstage | 0.05 | 0.05 | 1.40 | 0.80 |
| 2.surgery#2.seerstage | 0.00 | 0.01 | 6.30 | 0.41 |
| 2.surgery#3.seerstage | 0.00 | 0.00 | 6.70 | 0.16 |
| 2.surgery#4.seerstage | 0.01 | 0.01 | 1.50 | 0.76 |
| c.age#c.age | 4951.10 | 4907.20 | 3.40 | 0.45 |
| 2.category#2.seerstage | 0.09 | 0.10 | 2.20 | 0.64 |
| 2.category#3.seerstage | 0.16 | 0.16 | 0.60 | 0.94 |
| 3.category#2.seerstage | 0.05 | 0.05 | 0.00 | 1.00 |
| 3.category#3.seerstage | 0.06 | 0.06 | 4.30 | 0.57 |
| 3.category#4.seerstage | 0.10 | 0.10 | 1.40 | 0.78 |
| 1.radiation#2.seerstage | 0.09 | 0.09 | 0.00 | 1.00 |
| 1.radiation#3.seerstage | 0.19 | 0.20 | 1.10 | 0.89 |
| 1.radiation#4.seerstage | 0.05 | 0.05 | 1.80 | 0.71 |
| 2.radiation#2.seerstage | 0.16 | 0.16 | 0.40 | 0.94 |
| 2.radiation#3.seerstage | 0.23 | 0.23 | 0.50 | 0.95 |
| 2.radiation#4.seerstage | 0.09 | 0.09 | 1.10 | 0.85 |
| 1.radiation#1.surgery | 0.06 | 0.05 | 6.30 | 0.23 |
| 1.radiation#2.surgery | 0.00 | 0.00 | 0.00 | 1.00 |
| 2.radiation#1.surgery | 0.30 | 0.30 | 0.00 | 1.00 |
| 2.radiation#2.surgery | 0.01 | 0.01 | 0.00 | 1.00 |
| 2.category#c.age | 21.60 | 22.09 | 1.50 | 0.79 |
| 3.category#c.age | 15.93 | 16.65 | 2.50 | 0.66 |
| 1.marital#2.seerstage | 0.18 | 0.18 | 0.00 | 1.00 |
| 1.marital#3.seerstage | 0.29 | 0.31 | 4.10 | 0.59 |
| 1.marital#4.seerstage | 0.11 | 0.11 | 0.50 | 0.93 |
| 2.marital#2.seerstage | 0.02 | 0.01 | 3.60 | 0.46 |
| 2.marital#3.seerstage | 0.01 | 0.01 | 7.20 | 0.25 |
| 2.marital#4.seerstage | 0.00 | 0.00 | 4.40 | 0.32 |
| 2.race#1.surgery | 0.05 | 0.05 | 0.80 | 0.90 |
| 3.race#1.surgery | 0.02 | 0.02 | 1.20 | 0.83 |
| 3.race#2.surgery | 0.00 | 0.00 | 0.00 | 1.00 |
| 4.race#1.surgery | 0.03 | 0.02 | 2.30 | 0.72 |
| 1.marital#2.race | 0.09 | 0.09 | 2.30 | 0.70 |
| 1.marital#3.race | 0.05 | 0.05 | 1.30 | 0.80 |
| 1.marital#4.race | 0.03 | 0.03 | 1.70 | 0.75 |
| 2.marital#2.race | 0.02 | 0.01 | 2.90 | 0.64 |
| 2.marital#4.race | 0.01 | 0.00 | 9.40 | 0.05 |
| 2.seerstage#c.age | 17.22 | 17.20 | 0.10 | 0.99 |
| 3.seerstage#c.age | 29.54 | 29.43 | 0.40 | 0.95 |
| 4.seerstage#c.age | 10.06 | 9.99 | 0.30 | 0.96 |
| 2.race#2.seerstage | 0.06 | 0.06 | 1.90 | 0.73 |
| 2.race#3.seerstage | 0.08 | 0.08 | 0.00 | 1.00 |
| 2.race#4.seerstage | 0.01 | 0.01 | 2.40 | 0.62 |
| 3.race#2.seerstage | 0.01 | 0.01 | 0.90 | 0.81 |
| 3.race#3.seerstage | 0.03 | 0.03 | 1.30 | 0.86 |
| 3.race#4.seerstage | 0.01 | 0.01 | 1.60 | 0.76 |
| 4.race#2.seerstage | 0.01 | 0.01 | 1.00 | 0.78 |
| 4.race#3.seerstage | 0.03 | 0.02 | 3.90 | 0.59 |
| 4.race#4.seerstage | 0.00 | 0.00 | 2.10 | 0.65 |
| 2.category#1.surgery | 0.12 | 0.12 | 0.50 | 0.93 |
| 2.category#2.surgery | 0.00 | 0.00 | 0.00 | 1.00 |
| 3.category#1.surgery | 0.08 | 0.08 | 0.00 | 1.00 |
| 3.category#2.surgery | 0.01 | 0.01 | 1.50 | 0.76 |
| 1.radiation#2.category | 0.12 | 0.12 | 0.00 | 1.00 |
| 1.radiation#3.category | 0.08 | 0.10 | 4.60 | 0.39 |
| 2.radiation#2.category | 0.19 | 0.20 | 2.30 | 0.68 |
| 2.radiation#3.category | 0.14 | 0.14 | 0.50 | 0.94 |
| 2.category#1.marital | 0.21 | 0.23 | 5.20 | 0.36 |
| 2.category#2.marital | 0.02 | 0.01 | 5.50 | 0.32 |
| 3.category#1.marital | 0.17 | 0.17 | 1.60 | 0.77 |
| 3.category#2.marital | 0.01 | 0.01 | 3.40 | 0.56 |
| 2.category#2.race | 0.06 | 0.06 | 1.40 | 0.82 |
| 2.category#3.race | 0.02 | 0.01 | 3.20 | 0.51 |
| 2.category#4.race | 0.02 | 0.02 | 2.20 | 0.71 |
| 3.category#2.race | 0.03 | 0.03 | 2.90 | 0.63 |
| 3.category#3.race | 0.01 | 0.01 | 3.10 | 0.56 |
| 3.category#4.race | 0.01 | 0.00 | 3.90 | 0.41 |
| 1.surgery#c.age | 24.90 | 23.79 | 3.60 | 0.54 |
| 2.surgery#c.age | 1.07 | 1.02 | 0.60 | 0.92 |
| 2.race#c.age | 12.11 | 12.17 | 0.30 | 0.97 |
| 3.race#c.age | 3.73 | 3.59 | 0.80 | 0.88 |
| 4.race#c.age | 3.71 | 3.06 | 4.20 | 0.43 |

PALA: prostate cancer with subsequent lung cancer; bias: absolutely standardized difference; p: t test between matched treated and control group.

Tab S46. balance test between group mTPC1 with PALA and single lung cancer in the matched cohort

| Variable | Mean | |  |  |
| --- | --- | --- | --- | --- |
|  | Treated | Control | bias | P |
| age | 72.51 | 72.36 | 1.60 | 0.45 |
| 2.seerstage | 0.20 | 0.20 | 0.10 | 0.97 |
| 3.seerstage | 0.54 | 0.54 | 0.10 | 0.98 |
| 4.seerstage | 0.09 | 0.09 | 0.30 | 0.89 |
| 1.surgery | 0.25 | 0.24 | 2.80 | 0.30 |
| 2.surgery | 0.01 | 0.01 | 1.80 | 0.35 |
| 2.category | 0.33 | 0.33 | 0.30 | 0.91 |
| 3.category | 0.12 | 0.12 | 1.10 | 0.63 |
| 1.radiation | 0.39 | 0.36 | 5.70 | 0.03 |
| 2.radiation | 0.61 | 0.64 | 5.90 | 0.02 |
| 2.race | 0.17 | 0.17 | 0.40 | 0.89 |
| 3.race | 0.05 | 0.05 | 1.60 | 0.49 |
| 4.race | 0.04 | 0.04 | 2.30 | 0.36 |
| 5.race | 0.00 | 0.00 | 3.40 | 0.11 |
| 1.marital | 0.69 | 0.71 | 4.30 | 0.10 |
| 2.marital | 0.04 | 0.03 | 2.60 | 0.30 |
| 1.surgery#2.seerstage | 0.10 | 0.10 | 0.60 | 0.86 |
| 1.surgery#3.seerstage | 0.03 | 0.02 | 7.90 | 0.02 |
| 1.surgery#4.seerstage | 0.02 | 0.02 | 0.20 | 0.92 |
| 2.surgery#2.seerstage | 0.00 | 0.00 | 0.00 | 1.00 |
| 2.surgery#3.seerstage | 0.00 | 0.00 | 3.00 | 0.32 |
| 2.surgery#4.seerstage | 0.00 | 0.00 | 0.80 | 0.64 |
| 2.category#2.seerstage | 0.08 | 0.09 | 2.00 | 0.37 |
| 2.category#3.seerstage | 0.18 | 0.18 | 2.90 | 0.41 |
| 3.category#2.seerstage | 0.02 | 0.02 | 0.70 | 0.71 |
| 3.category#3.seerstage | 0.04 | 0.05 | 3.20 | 0.38 |
| 3.category#4.seerstage | 0.04 | 0.04 | 0.10 | 0.95 |
| c.age#c.age | 5315.50 | 5291.80 | 1.80 | 0.41 |
| 1.radiation#1.surgery | 0.05 | 0.04 | 5.60 | 0.02 |
| 1.radiation#2.surgery | 0.00 | 0.00 | 1.30 | 0.47 |
| 2.radiation#1.surgery | 0.20 | 0.20 | 0.30 | 0.92 |
| 2.radiation#2.surgery | 0.00 | 0.00 | 1.30 | 0.55 |
| 2.seerstage#c.age | 14.52 | 14.55 | 0.10 | 0.97 |
| 3.seerstage#c.age | 38.96 | 38.84 | 0.40 | 0.91 |
| 4.seerstage#c.age | 6.51 | 6.43 | 0.40 | 0.87 |
| 1.marital#2.seerstage | 0.14 | 0.14 | 0.60 | 0.79 |
| 1.marital#3.seerstage | 0.37 | 0.39 | 4.40 | 0.22 |
| 1.marital#4.seerstage | 0.06 | 0.06 | 0.40 | 0.87 |
| 2.marital#2.seerstage | 0.01 | 0.01 | 0.30 | 0.86 |
| 2.marital#3.seerstage | 0.02 | 0.02 | 1.70 | 0.61 |
| 2.marital#4.seerstage | 0.00 | 0.00 | 2.20 | 0.25 |
| 2.category#c.age | 23.83 | 23.71 | 0.40 | 0.89 |
| 3.category#c.age | 8.68 | 8.98 | 1.20 | 0.64 |
| 2.race#c.age | 12.01 | 11.79 | 0.90 | 0.75 |
| 3.race#c.age | 3.68 | 3.36 | 1.80 | 0.46 |
| 4.race#c.age | 3.26 | 2.90 | 2.40 | 0.35 |
| 5.race#c.age | 0.25 | 0.11 | 3.40 | 0.14 |
| 2.category#1.surgery | 0.09 | 0.09 | 0.60 | 0.82 |
| 2.category#2.surgery | 0.00 | 0.00 | 2.80 | 0.08 |
| 3.category#1.surgery | 0.03 | 0.03 | 1.50 | 0.50 |
| 3.category#2.surgery | 0.00 | 0.00 | 0.40 | 0.81 |
| 2.race#2.seerstage | 0.04 | 0.03 | 0.90 | 0.67 |
| 2.race#3.seerstage | 0.09 | 0.09 | 0.30 | 0.93 |
| 2.race#4.seerstage | 0.02 | 0.01 | 1.30 | 0.58 |
| 3.race#2.seerstage | 0.01 | 0.01 | 0.20 | 0.89 |
| 3.race#3.seerstage | 0.03 | 0.02 | 3.00 | 0.39 |
| 3.race#4.seerstage | 0.00 | 0.00 | 0.00 | 1.00 |
| 4.race#2.seerstage | 0.01 | 0.01 | 1.00 | 0.56 |
| 4.race#3.seerstage | 0.02 | 0.02 | 3.70 | 0.29 |
| 4.race#4.seerstage | 0.00 | 0.00 | 0.00 | 1.00 |
| 5.race#2.seerstage | 0.00 | 0.00 | 0.90 | 0.56 |
| 5.race#3.seerstage | 0.00 | 0.00 | 5.80 | 0.03 |
| 2.race#1.surgery | 0.04 | 0.03 | 3.70 | 0.19 |
| 2.race#2.surgery | 0.00 | 0.00 | 0.90 | 0.74 |
| 3.race#1.surgery | 0.01 | 0.01 | 0.30 | 0.91 |
| 4.race#1.surgery | 0.01 | 0.01 | 1.50 | 0.52 |
| 5.race#1.surgery | 0.00 | 0.00 | 2.50 | 0.16 |
| 2.race#1.radiation | 0.07 | 0.06 | 4.40 | 0.09 |
| 2.race#2.radiation | 0.11 | 0.12 | 3.40 | 0.26 |
| 3.race#1.radiation | 0.02 | 0.01 | 2.90 | 0.15 |
| 3.race#2.radiation | 0.03 | 0.03 | 0.40 | 0.88 |
| 4.race#1.radiation | 0.01 | 0.01 | 3.80 | 0.07 |
| 4.race#2.radiation | 0.03 | 0.03 | 0.00 | 1.00 |
| 5.race#1.radiation | 0.00 | 0.00 | 3.30 | 0.16 |
| 5.race#2.radiation | 0.00 | 0.00 | 1.60 | 0.41 |
| 2.race#2.category | 0.05 | 0.04 | 4.50 | 0.10 |
| 2.race#3.category | 0.02 | 0.02 | 1.50 | 0.53 |
| 3.race#2.category | 0.02 | 0.02 | 1.30 | 0.60 |
| 3.race#3.category | 0.00 | 0.00 | 0.40 | 0.83 |
| 4.race#2.category | 0.01 | 0.01 | 0.60 | 0.81 |
| 4.race#3.category | 0.00 | 0.00 | 2.70 | 0.10 |
| 5.race#2.category | 0.00 | 0.00 | 0.00 | 1.00 |
| 5.race#3.category | 0.00 | 0.00 | 1.80 | 0.32 |
| 1.marital#c.age | 49.91 | 51.30 | 4.10 | 0.12 |
| 2.marital#c.age | 2.58 | 2.19 | 3.00 | 0.26 |
| 1.marital#1.surgery | 0.18 | 0.17 | 0.90 | 0.73 |
| 1.marital#2.surgery | 0.01 | 0.00 | 1.10 | 0.58 |
| 2.marital#1.surgery | 0.01 | 0.01 | 2.40 | 0.34 |
| 2.marital#2.surgery | 0.00 | 0.00 | 2.10 | 0.32 |
| 1.marital#2.category | 0.24 | 0.24 | 0.30 | 0.93 |
| 1.marital#3.category | 0.08 | 0.09 | 1.80 | 0.43 |
| 2.marital#2.category | 0.01 | 0.01 | 0.80 | 0.75 |
| 2.marital#3.category | 0.00 | 0.00 | 1.10 | 0.64 |

PALA: prostate cancer with subsequent lung cancer; bias: absolutely standardized difference; p: t test between matched treated and control group.

Tab S47. balance test between group mTPC2 with PALA and single lung cancer in the matched cohort

| Variable | Mean | |  |  |
| --- | --- | --- | --- | --- |
|  | Treated | Control | bias | P |
| age | 76.45 | 76.44 | 0.10 | 0.95 |
| 2.seerstage | 0.18 | 0.18 | 0.10 | 0.95 |
| 3.seerstage | 0.53 | 0.52 | 1.30 | 0.69 |
| 4.seerstage | 0.12 | 0.12 | 0.40 | 0.86 |
| 2.category | 0.19 | 0.19 | 1.00 | 0.63 |
| 3.category | 0.05 | 0.05 | 0.30 | 0.83 |
| 1.surgery | 0.22 | 0.21 | 0.30 | 0.89 |
| 2.surgery | 0.01 | 0.00 | 5.00 | 0.00 |
| 1.radiation | 0.34 | 0.33 | 2.80 | 0.23 |
| 2.radiation | 0.66 | 0.67 | 2.80 | 0.23 |
| 2.race | 0.15 | 0.16 | 2.30 | 0.35 |
| 3.race | 0.05 | 0.04 | 3.00 | 0.14 |
| 4.race | 0.05 | 0.04 | 3.00 | 0.17 |
| 5.race | 0.00 | 0.00 | 0.90 | 0.67 |
| 1.marital | 0.68 | 0.67 | 1.00 | 0.67 |
| 2.marital | 0.04 | 0.04 | 0.70 | 0.76 |
| 1.surgery#2.seerstage | 0.09 | 0.09 | 0.20 | 0.93 |
| 1.surgery#3.seerstage | 0.02 | 0.01 | 5.60 | 0.06 |
| 1.surgery#4.seerstage | 0.02 | 0.02 | 0.30 | 0.87 |
| 2.surgery#2.seerstage | 0.00 | 0.00 | 1.60 | 0.16 |
| 2.surgery#3.seerstage | 0.00 | 0.00 | 3.60 | 0.06 |
| 2.surgery#4.seerstage | 0.00 | 0.00 | 2.10 | 0.09 |
| c.age#c.age | 5897.00 | 5895.30 | 0.10 | 0.95 |
| 2.category#2.seerstage | 0.04 | 0.05 | 0.50 | 0.74 |
| 2.category#3.seerstage | 0.11 | 0.10 | 3.60 | 0.24 |
| 3.category#2.seerstage | 0.01 | 0.01 | 0.60 | 0.63 |
| 3.category#3.seerstage | 0.02 | 0.02 | 0.50 | 0.86 |
| 3.category#4.seerstage | 0.01 | 0.01 | 0.40 | 0.69 |
| 1.radiation#1.surgery | 0.03 | 0.02 | 3.20 | 0.08 |
| 1.radiation#2.surgery | 0.00 | 0.00 | 1.50 | 0.05 |
| 2.radiation#1.surgery | 0.19 | 0.19 | 1.40 | 0.57 |
| 2.radiation#2.surgery | 0.01 | 0.00 | 5.20 | 0.00 |
| 2.seerstage#c.age | 13.49 | 13.44 | 0.20 | 0.94 |
| 3.seerstage#c.age | 40.33 | 39.97 | 1.30 | 0.69 |
| 4.seerstage#c.age | 9.21 | 9.10 | 0.40 | 0.85 |
| 1.marital#2.seerstage | 0.12 | 0.12 | 0.20 | 0.92 |
| 1.marital#3.seerstage | 0.36 | 0.35 | 1.30 | 0.68 |
| 1.marital#4.seerstage | 0.08 | 0.08 | 0.20 | 0.93 |
| 2.marital#2.seerstage | 0.01 | 0.00 | 0.30 | 0.87 |
| 2.marital#3.seerstage | 0.02 | 0.02 | 0.60 | 0.86 |
| 2.marital#4.seerstage | 0.01 | 0.01 | 0.40 | 0.88 |
| 1.radiation#2.category | 0.06 | 0.06 | 1.30 | 0.49 |
| 1.radiation#3.category | 0.02 | 0.02 | 0.20 | 0.86 |
| 2.radiation#2.category | 0.13 | 0.13 | 0.20 | 0.94 |
| 2.radiation#3.category | 0.03 | 0.03 | 0.20 | 0.89 |
| 2.race#c.age | 10.91 | 11.46 | 2.30 | 0.38 |
| 3.race#c.age | 4.01 | 3.45 | 3.10 | 0.15 |
| 4.race#c.age | 4.02 | 3.48 | 3.30 | 0.17 |
| 5.race#c.age | 0.25 | 0.21 | 0.80 | 0.73 |
| 2.race#1.surgery | 0.03 | 0.03 | 1.20 | 0.64 |
| 2.race#2.surgery | 0.00 | 0.00 | 1.60 | 0.32 |
| 3.race#1.surgery | 0.01 | 0.01 | 1.00 | 0.60 |
| 3.race#2.surgery | 0.00 | 0.00 | 2.40 | 0.16 |
| 4.race#1.surgery | 0.01 | 0.01 | 2.00 | 0.35 |
| 4.race#2.surgery | 0.00 | 0.00 | 4.50 | 0.03 |
| 5.race#1.surgery | 0.00 | 0.00 | 1.00 | 0.71 |
| 1.radiation#2.seerstage | 0.08 | 0.07 | 0.70 | 0.69 |
| 1.radiation#3.seerstage | 0.18 | 0.16 | 4.00 | 0.20 |
| 1.radiation#4.seerstage | 0.04 | 0.04 | 0.60 | 0.78 |
| 2.radiation#2.seerstage | 0.10 | 0.10 | 0.50 | 0.79 |
| 2.radiation#3.seerstage | 0.35 | 0.36 | 1.90 | 0.56 |
| 2.radiation#4.seerstage | 0.08 | 0.08 | 0.00 | 1.00 |
| 2.race#2.category | 0.03 | 0.02 | 1.70 | 0.42 |
| 2.race#3.category | 0.00 | 0.00 | 0.90 | 0.46 |
| 3.race#2.category | 0.01 | 0.01 | 2.00 | 0.22 |
| 3.race#3.category | 0.00 | 0.00 | 0.30 | 0.78 |
| 4.race#2.category | 0.01 | 0.01 | 1.70 | 0.38 |
| 4.race#3.category | 0.00 | 0.00 | 1.40 | 0.08 |
| 5.race#2.category | 0.00 | 0.00 | 0.00 | 1.00 |
| 1.marital#2.category | 0.13 | 0.13 | 0.30 | 0.89 |
| 1.marital#3.category | 0.03 | 0.03 | 0.60 | 0.69 |
| 2.marital#2.category | 0.01 | 0.01 | 0.90 | 0.67 |
| 2.marital#3.category | 0.00 | 0.00 | 0.00 | 1.00 |
| 1.marital#c.age | 51.61 | 51.35 | 0.80 | 0.76 |
| 2.marital#c.age | 2.78 | 2.84 | 0.40 | 0.87 |
| 1.marital#2.race | 0.08 | 0.09 | 1.60 | 0.53 |
| 1.marital#3.race | 0.04 | 0.04 | 2.10 | 0.30 |
| 1.marital#4.race | 0.03 | 0.03 | 0.90 | 0.68 |
| 1.marital#5.race | 0.00 | 0.00 | 0.00 | 1.00 |
| 2.marital#2.race | 0.01 | 0.01 | 2.50 | 0.35 |
| 2.marital#3.race | 0.00 | 0.00 | 0.60 | 0.71 |
| 2.marital#4.race | 0.00 | 0.00 | 0.60 | 0.81 |
| 2.marital#5.race | 0.00 | 0.00 | 1.30 | 0.32 |
| 2.race#1.radiation | 0.04 | 0.04 | 0.60 | 0.77 |
| 2.race#2.radiation | 0.11 | 0.11 | 2.30 | 0.37 |
| 3.race#1.radiation | 0.02 | 0.01 | 3.50 | 0.06 |
| 3.race#2.radiation | 0.03 | 0.03 | 1.00 | 0.64 |
| 4.race#1.radiation | 0.01 | 0.01 | 1.70 | 0.40 |
| 4.race#2.radiation | 0.04 | 0.03 | 2.50 | 0.29 |
| 5.race#1.radiation | 0.00 | 0.00 | 1.50 | 0.41 |
| 5.race#2.radiation | 0.00 | 0.00 | 0.00 | 1.00 |
| 1.surgery#2.category | 0.05 | 0.05 | 0.10 | 0.96 |
| 1.surgery#3.category | 0.01 | 0.01 | 0.40 | 0.77 |
| 2.surgery#2.category | 0.00 | 0.00 | 2.30 | 0.08 |
| 2.surgery#3.category | 0.00 | 0.00 | 0.30 | 0.71 |
| 1.marital#1.radiation | 0.24 | 0.23 | 3.70 | 0.10 |
| 1.marital#2.radiation | 0.43 | 0.44 | 2.40 | 0.32 |
| 2.marital#1.radiation | 0.01 | 0.01 | 1.90 | 0.38 |
| 2.marital#2.radiation | 0.03 | 0.03 | 0.30 | 0.89 |
| 2.race#2.seerstage | 0.03 | 0.03 | 0.50 | 0.77 |
| 2.race#3.seerstage | 0.08 | 0.08 | 2.40 | 0.43 |
| 2.race#4.seerstage | 0.02 | 0.02 | 0.20 | 0.93 |
| 3.race#2.seerstage | 0.01 | 0.01 | 0.50 | 0.68 |
| 3.race#3.seerstage | 0.03 | 0.02 | 5.90 | 0.05 |
| 3.race#4.seerstage | 0.01 | 0.01 | 0.00 | 1.00 |
| 4.race#2.seerstage | 0.01 | 0.01 | 0.80 | 0.60 |
| 4.race#3.seerstage | 0.03 | 0.02 | 2.80 | 0.38 |
| 4.race#4.seerstage | 0.01 | 0.01 | 1.70 | 0.47 |
| 5.race#2.seerstage | 0.00 | 0.00 | 0.70 | 0.66 |
| 5.race#3.seerstage | 0.00 | 0.00 | 2.70 | 0.32 |
| 1.radiation#c.age | 26.18 | 25.21 | 2.80 | 0.25 |
| 2.radiation#c.age | 50.27 | 51.22 | 2.70 | 0.27 |
| 1.marital#1.surgery | 0.16 | 0.15 | 1.10 | 0.63 |
| 1.marital#2.surgery | 0.00 | 0.00 | 3.80 | 0.00 |
| 2.marital#1.surgery | 0.01 | 0.01 | 1.40 | 0.55 |
| 2.marital#2.surgery | 0.00 | 0.00 | 2.40 | 0.08 |
| 2.category#c.age | 14.54 | 14.22 | 1.10 | 0.65 |
| 3.category#c.age | 3.64 | 3.55 | 0.40 | 0.81 |

PALA: prostate cancer with subsequent lung cancer; bias: absolutely standardized difference; p: t test between matched treated and control group.

Tab S48.balance test between PALA and single prostate cancer in the matched cohort

| Variable | Mean | |  |  |
| --- | --- | --- | --- | --- |
|  | Treated | Control | bias | P |
| age | 68.17 | 68.17 | 0.00 | 0.99 |
| 2.category | 0.56 | 0.56 | 0.00 | 0.99 |
| 3.category | 0.10 | 0.10 | 0.00 | 1.00 |
| 3.seerstage | 0.02 | 0.02 | 0.00 | 1.00 |
| 4.seerstage | 0.12 | 0.12 | 0.00 | 0.98 |
| 2.race | 0.17 | 0.17 | 0.00 | 0.98 |
| 3.race | 0.05 | 0.05 | 0.00 | 1.00 |
| 4.race | 0.05 | 0.05 | 0.00 | 1.00 |
| 1.radiation | 0.42 | 0.42 | 0.00 | 0.99 |
| 2.radiation | 0.57 | 0.57 | 0.00 | 0.99 |
| 1.surgery | 0.36 | 0.36 | 0.00 | 0.99 |
| 2.surgery | 0.01 | 0.01 | 0.00 | 1.00 |
| 3.seerstage#2.category | 0.01 | 0.01 | 0.00 | 1.00 |
| 4.seerstage#2.category | 0.02 | 0.01 | 0.10 | 0.94 |
| 4.seerstage#3.category | 0.10 | 0.10 | 0.00 | 1.00 |
| c.age#c.age | 4705.80 | 4706.10 | 0.00 | 0.99 |
| 2.category#c.age | 37.75 | 37.74 | 0.00 | 0.99 |
| 3.category#c.age | 6.66 | 6.66 | 0.00 | 1.00 |
| 2.race#c.age | 11.43 | 11.44 | 0.10 | 0.98 |
| 3.race#c.age | 3.52 | 3.52 | 0.00 | 1.00 |
| 4.race#c.age | 3.72 | 3.72 | 0.00 | 1.00 |
| 1.surgery#2.category | 0.20 | 0.20 | 0.00 | 1.00 |
| 1.surgery#3.category | 0.07 | 0.07 | 0.00 | 1.00 |
| 2.surgery#2.category | 0.01 | 0.01 | 0.00 | 1.00 |
| 2.surgery#3.category | 0.00 | 0.00 | 0.00 | 1.00 |
| 1.radiation#3.seerstage | 0.01 | 0.01 | 0.00 | 1.00 |
| 1.radiation#4.seerstage | 0.04 | 0.04 | 0.10 | 0.96 |
| 2.radiation#3.seerstage | 0.01 | 0.01 | 0.10 | 0.94 |
| 2.radiation#4.seerstage | 0.09 | 0.09 | 0.00 | 1.00 |
| 2.race#2.category | 0.10 | 0.10 | 0.00 | 1.00 |
| 2.race#3.category | 0.01 | 0.01 | 0.00 | 1.00 |
| 3.race#2.category | 0.03 | 0.03 | 0.00 | 1.00 |
| 3.race#3.category | 0.00 | 0.00 | 0.00 | 1.00 |
| 4.race#2.category | 0.03 | 0.03 | 0.00 | 1.00 |
| 4.race#3.category | 0.00 | 0.00 | 0.00 | 1.00 |
| 1.radiation#2.category | 0.25 | 0.25 | 0.00 | 0.98 |
| 1.radiation#3.category | 0.03 | 0.03 | 0.00 | 1.00 |
| 2.radiation#2.category | 0.30 | 0.30 | 0.00 | 0.99 |
| 2.radiation#3.category | 0.06 | 0.06 | 0.00 | 1.00 |
| 1.surgery#2.race | 0.06 | 0.06 | 0.10 | 0.97 |
| 1.surgery#3.race | 0.02 | 0.02 | 0.00 | 1.00 |
| 1.surgery#4.race | 0.02 | 0.02 | 0.00 | 1.00 |
| 2.surgery#2.race | 0.00 | 0.00 | 0.00 | 1.00 |
| 2.surgery#3.race | 0.00 | 0.00 | 0.00 | 1.00 |
| 2.surgery#4.race | 0.00 | 0.00 | 0.00 | 1.00 |
| 1.radiation#2.race | 0.07 | 0.07 | 0.00 | 1.00 |
| 1.radiation#3.race | 0.02 | 0.02 | 0.10 | 0.95 |
| 1.radiation#4.race | 0.02 | 0.02 | 0.00 | 1.00 |
| 2.radiation#2.race | 0.10 | 0.10 | 0.00 | 1.00 |
| 2.radiation#3.race | 0.03 | 0.03 | 0.10 | 0.96 |
| 2.radiation#4.race | 0.03 | 0.03 | 0.00 | 1.00 |
| 1.surgery#3.seerstage | 0.00 | 0.00 | 0.00 | 1.00 |
| 1.surgery#4.seerstage | 0.07 | 0.07 | 0.00 | 1.00 |
| 2.surgery#3.seerstage | 0.00 | 0.00 | 0.00 | 1.00 |
| 2.surgery#4.seerstage | 0.00 | 0.00 | 0.00 | 1.00 |
| 2.race#3.seerstage | 0.00 | 0.00 | 0.00 | 1.00 |
| 2.race#4.seerstage | 0.02 | 0.02 | 0.00 | 1.00 |
| 3.race#3.seerstage | 0.00 | 0.00 | 0.30 | 0.84 |
| 3.race#4.seerstage | 0.00 | 0.00 | 0.20 | 0.90 |
| 4.race#3.seerstage | 0.00 | 0.00 | 0.00 | 1.00 |
| 4.race#4.seerstage | 0.01 | 0.01 | 0.00 | 1.00 |
| 3.seerstage#c.age | 1.49 | 1.48 | 0.00 | 1.00 |
| 4.seerstage#c.age | 8.50 | 8.49 | 0.00 | 0.98 |
| 1.surgery#1.radiation | 0.04 | 0.04 | 0.00 | 1.00 |
| 1.surgery#2.radiation | 0.32 | 0.32 | 0.00 | 1.00 |
| 2.surgery#1.radiation | 0.00 | 0.00 | 0.00 | 1.00 |
| 2.surgery#2.radiation | 0.01 | 0.01 | 0.00 | 1.00 |

PALA: prostate cancer with subsequent lung cancer; bias: absolutely standardized difference; p: t test between matched treated and control group.

Tab S49. balance test between group sTPC with PALA and single prostate cancer in the matched cohort

| Variable | Mean | |  |  |
| --- | --- | --- | --- | --- |
|  | Treated | Control | bias | P |
| age | 69.52 | 69.60 | 0.90 | 0.86 |
| 1.surgery | 0.22 | 0.22 | 0.40 | 0.94 |
| 2.surgery | 0.03 | 0.03 | 1.10 | 0.87 |
| 1.radiation | 0.26 | 0.26 | 0.00 | 1.00 |
| 2.radiation | 0.73 | 0.73 | 0.00 | 1.00 |
| 2.race | 0.19 | 0.19 | 0.40 | 0.94 |
| 3.race | 0.11 | 0.11 | 0.50 | 0.93 |
| 3.seerstage | 0.08 | 0.08 | 0.70 | 0.92 |
| 4.seerstage | 0.18 | 0.18 | 0.40 | 0.94 |
| 2.category | 0.37 | 0.37 | 0.00 | 1.00 |
| 3.category | 0.12 | 0.12 | 0.50 | 0.93 |
| 1.marital | 0.63 | 0.63 | 0.70 | 0.91 |
| 2.marital | 0.13 | 0.13 | 0.00 | 1.00 |
| 2.category#3.seerstage | 0.04 | 0.04 | 0.00 | 1.00 |
| 2.category#4.seerstage | 0.02 | 0.02 | 0.00 | 1.00 |
| 3.category#4.seerstage | 0.12 | 0.12 | 0.50 | 0.93 |
| c.age#c.age | 4891.80 | 4902.20 | 0.90 | 0.87 |
| 3.seerstage#1.radiation | 0.03 | 0.03 | 0.00 | 1.00 |
| 3.seerstage#2.radiation | 0.05 | 0.05 | 0.90 | 0.90 |
| 4.seerstage#1.radiation | 0.03 | 0.03 | 0.00 | 1.00 |
| 4.seerstage#2.radiation | 0.15 | 0.15 | 0.50 | 0.94 |
| 1.surgery#c.age | 14.96 | 15.14 | 0.60 | 0.92 |
| 2.surgery#c.age | 2.22 | 2.12 | 1.00 | 0.88 |
| 2.category#c.age | 25.46 | 25.46 | 0.00 | 1.00 |
| 3.category#c.age | 8.46 | 8.61 | 0.60 | 0.91 |
| 2.race#c.age | 12.58 | 12.50 | 0.30 | 0.96 |
| 3.race#c.age | 7.86 | 8.02 | 0.70 | 0.90 |
| 1.marital#3.seerstage | 0.06 | 0.06 | 0.90 | 0.90 |
| 1.marital#4.seerstage | 0.12 | 0.12 | 0.50 | 0.93 |
| 2.marital#3.seerstage | 0.00 | 0.00 | 0.00 | 1.00 |
| 2.marital#4.seerstage | 0.01 | 0.01 | 0.00 | 1.00 |
| 2.category#2.race | 0.07 | 0.07 | 0.00 | 1.00 |
| 2.category#3.race | 0.05 | 0.05 | 0.00 | 1.00 |
| 3.category#2.race | 0.01 | 0.01 | 0.00 | 1.00 |
| 3.category#3.race | 0.01 | 0.01 | 1.60 | 0.74 |
| 1.radiation#1.surgery | 0.02 | 0.02 | 0.00 | 1.00 |
| 1.radiation#2.surgery | 0.00 | 0.00 | 0.00 | 1.00 |
| 2.radiation#1.surgery | 0.19 | 0.20 | 0.40 | 0.94 |
| 2.radiation#2.surgery | 0.03 | 0.03 | 1.10 | 0.87 |
| 1.marital#1.surgery | 0.15 | 0.14 | 0.40 | 0.94 |
| 1.marital#2.surgery | 0.01 | 0.01 | 1.60 | 0.80 |
| 2.marital#1.surgery | 0.01 | 0.01 | 0.00 | 1.00 |
| 2.marital#2.surgery | 0.02 | 0.02 | 0.00 | 1.00 |
| 1.marital#1.radiation | 0.17 | 0.17 | 0.00 | 1.00 |
| 1.marital#2.radiation | 0.46 | 0.46 | 0.70 | 0.91 |
| 2.marital#1.radiation | 0.02 | 0.02 | 0.00 | 1.00 |
| 2.marital#2.radiation | 0.10 | 0.10 | 0.00 | 1.00 |
| 1.marital#2.race | 0.09 | 0.09 | 0.60 | 0.92 |
| 1.marital#3.race | 0.08 | 0.08 | 0.60 | 0.91 |
| 2.marital#2.race | 0.03 | 0.03 | 0.00 | 1.00 |
| 2.marital#3.race | 0.02 | 0.02 | 0.00 | 1.00 |
| 3.seerstage#2.race | 0.02 | 0.02 | 1.50 | 0.83 |
| 3.seerstage#3.race | 0.01 | 0.01 | 0.00 | 1.00 |
| 4.seerstage#2.race | 0.02 | 0.02 | 0.00 | 1.00 |
| 4.seerstage#3.race | 0.02 | 0.02 | 1.10 | 0.83 |
| 1.marital#2.category | 0.25 | 0.25 | 0.00 | 1.00 |
| 1.marital#3.category | 0.09 | 0.09 | 0.00 | 1.00 |
| 2.marital#2.category | 0.03 | 0.03 | 0.00 | 1.00 |
| 2.marital#3.category | 0.00 | 0.00 | 0.00 | 1.00 |
| 3.seerstage#1.surgery | 0.01 | 0.00 | 2.20 | 0.71 |
| 4.seerstage#1.surgery | 0.07 | 0.07 | 1.20 | 0.82 |
| 4.seerstage#2.surgery | 0.01 | 0.01 | 1.90 | 0.74 |
| 2.category#1.surgery | 0.08 | 0.08 | 0.00 | 1.00 |
| 2.category#2.surgery | 0.01 | 0.01 | 0.00 | 1.00 |
| 3.category#1.surgery | 0.05 | 0.06 | 0.60 | 0.90 |
| 3.category#2.surgery | 0.00 | 0.00 | 0.00 | 1.00 |

PALA: prostate cancer with subsequent lung cancer; bias: absolutely standardized difference; p: t test between matched treated and control group.

Tab S50. balance test between group mTPC1 with PALA and single prostate cancer in the matched cohort

| Variable | Mean | |  |  |
| --- | --- | --- | --- | --- |
|  | Treated | Control | bias | P |
| age | 69.73 | 69.72 | 0.10 | 0.97 |
| 3.seerstage | 0.02 | 0.02 | 0.20 | 0.92 |
| 4.seerstage | 0.10 | 0.10 | 0.10 | 0.96 |
| 2.category | 0.46 | 0.46 | 0.10 | 0.98 |
| 3.category | 0.08 | 0.08 | 0.00 | 1.00 |
| 1.radiation | 0.45 | 0.45 | 0.10 | 0.98 |
| 2.radiation | 0.55 | 0.55 | 0.10 | 0.98 |
| 2.race | 0.19 | 0.19 | 0.10 | 0.97 |
| 3.race | 0.05 | 0.05 | 0.00 | 1.00 |
| 4.race | 0.05 | 0.05 | 0.00 | 1.00 |
| 1.surgery | 0.31 | 0.31 | 0.00 | 1.00 |
| 2.surgery | 0.01 | 0.01 | 0.00 | 1.00 |
| 1.marital | 0.65 | 0.65 | 0.00 | 1.00 |
| 2.marital | 0.12 | 0.12 | 0.10 | 0.97 |
| 2.category#3.seerstage | 0.01 | 0.01 | 0.40 | 0.88 |
| 2.category#4.seerstage | 0.02 | 0.02 | 0.00 | 1.00 |
| 3.category#4.seerstage | 0.08 | 0.08 | 0.00 | 1.00 |
| c.age#c.age | 4920.70 | 4919.40 | 0.10 | 0.97 |
| 1.radiation#3.seerstage | 0.01 | 0.01 | 0.50 | 0.86 |
| 1.radiation#4.seerstage | 0.03 | 0.03 | 0.00 | 1.00 |
| 2.radiation#3.seerstage | 0.01 | 0.01 | 0.00 | 1.00 |
| 2.radiation#4.seerstage | 0.07 | 0.07 | 0.10 | 0.96 |
| 1.surgery#2.category | 0.12 | 0.12 | 0.10 | 0.97 |
| 1.surgery#3.category | 0.05 | 0.05 | 0.00 | 1.00 |
| 2.surgery#2.category | 0.00 | 0.00 | 0.00 | 1.00 |
| 2.surgery#3.category | 0.00 | 0.00 | 0.00 | 1.00 |
| 1.surgery#1.radiation | 0.04 | 0.04 | 0.20 | 0.94 |
| 1.surgery#2.radiation | 0.27 | 0.27 | 0.10 | 0.98 |
| 2.surgery#1.radiation | 0.00 | 0.00 | 0.00 | 1.00 |
| 2.surgery#2.radiation | 0.01 | 0.01 | 0.00 | 1.00 |
| 1.marital#3.seerstage | 0.01 | 0.01 | 0.00 | 1.00 |
| 1.marital#4.seerstage | 0.07 | 0.07 | 0.00 | 1.00 |
| 2.marital#3.seerstage | 0.00 | 0.00 | 0.00 | 1.00 |
| 2.marital#4.seerstage | 0.01 | 0.01 | 0.30 | 0.90 |
| 1.marital#1.surgery | 0.23 | 0.23 | 0.00 | 1.00 |
| 1.marital#2.surgery | 0.00 | 0.00 | 0.00 | 1.00 |
| 2.marital#1.surgery | 0.01 | 0.01 | 0.30 | 0.91 |
| 2.marital#2.surgery | 0.01 | 0.01 | 0.00 | 1.00 |
| 2.race#3.seerstage | 0.01 | 0.01 | 0.00 | 1.00 |
| 2.race#4.seerstage | 0.02 | 0.02 | 0.00 | 1.00 |
| 3.race#3.seerstage | 0.00 | 0.00 | 0.00 | 1.00 |
| 3.race#4.seerstage | 0.00 | 0.00 | 0.00 | 1.00 |
| 4.race#3.seerstage | 0.00 | 0.00 | 0.00 | 1.00 |
| 4.race#4.seerstage | 0.01 | 0.01 | 0.00 | 1.00 |
| 1.surgery#c.age | 20.51 | 20.50 | 0.00 | 0.99 |
| 2.surgery#c.age | 0.93 | 0.93 | 0.00 | 1.00 |
| 2.race#c.age | 12.53 | 12.55 | 0.10 | 0.98 |
| 3.race#c.age | 3.43 | 3.43 | 0.00 | 1.00 |
| 4.race#c.age | 3.48 | 3.48 | 0.00 | 1.00 |
| 1.marital#1.radiation | 0.31 | 0.31 | 0.00 | 1.00 |
| 1.marital#2.radiation | 0.34 | 0.34 | 0.00 | 1.00 |
| 2.marital#1.radiation | 0.03 | 0.03 | 0.00 | 1.00 |
| 2.marital#2.radiation | 0.09 | 0.09 | 0.10 | 0.96 |
| 1.surgery#3.seerstage | 0.00 | 0.00 | 0.70 | 0.71 |
| 1.surgery#4.seerstage | 0.06 | 0.06 | 0.10 | 0.95 |
| 2.surgery#4.seerstage | 0.00 | 0.00 | 0.00 | 1.00 |
| 1.surgery#2.race | 0.05 | 0.05 | 0.20 | 0.95 |
| 1.surgery#3.race | 0.01 | 0.01 | 0.00 | 1.00 |
| 1.surgery#4.race | 0.01 | 0.01 | 0.00 | 1.00 |
| 2.surgery#2.race | 0.00 | 0.00 | 0.00 | 1.00 |
| 2.surgery#3.race | 0.00 | 0.00 | 0.00 | 1.00 |
| 1.marital#2.race | 0.10 | 0.10 | 0.00 | 1.00 |
| 1.marital#3.race | 0.04 | 0.04 | 0.00 | 1.00 |
| 1.marital#4.race | 0.03 | 0.03 | 0.00 | 1.00 |
| 2.marital#2.race | 0.02 | 0.02 | 0.30 | 0.92 |
| 2.marital#3.race | 0.01 | 0.01 | 0.00 | 1.00 |
| 2.marital#4.race | 0.01 | 0.01 | 0.00 | 1.00 |
| 1.marital#2.category | 0.32 | 0.32 | 0.00 | 1.00 |
| 1.marital#3.category | 0.05 | 0.05 | 0.00 | 1.00 |
| 2.marital#2.category | 0.04 | 0.04 | 0.00 | 1.00 |
| 2.marital#3.category | 0.01 | 0.01 | 0.00 | 1.00 |
| 2.race#2.category | 0.08 | 0.08 | 0.20 | 0.96 |
| 2.race#3.category | 0.01 | 0.01 | 0.00 | 1.00 |
| 3.race#2.category | 0.02 | 0.02 | 0.00 | 1.00 |
| 3.race#3.category | 0.00 | 0.00 | 0.00 | 1.00 |
| 4.race#2.category | 0.02 | 0.02 | 0.00 | 1.00 |
| 4.race#3.category | 0.00 | 0.00 | 0.00 | 1.00 |
| 1.radiation#2.category | 0.22 | 0.22 | 0.10 | 0.97 |
| 1.radiation#3.category | 0.03 | 0.03 | 0.00 | 1.00 |
| 2.radiation#2.category | 0.24 | 0.24 | 0.00 | 1.00 |
| 2.radiation#3.category | 0.05 | 0.05 | 0.00 | 1.00 |
| 1.marital#c.age | 45.20 | 45.20 | 0.00 | 1.00 |
| 2.marital#c.age | 8.53 | 8.54 | 0.10 | 0.98 |

PALA: prostate cancer with subsequent lung cancer; bias: absolutely standardized difference; p: t test between matched treated and control group.

Tab S51. balance test between group mTPC2 with PALA and single prostate cancer in the matched cohort

| Variable | Mean | |  |  |
| --- | --- | --- | --- | --- |
|  | Treated | Control | bias | P |
| 1.marital | 0.72 | 0.72 | 0.00 | 1.00 |
| 1.marital#1.radiation | 0.32 | 0.32 | 0.10 | 0.98 |
| 1.marital#1.surgery | 0.34 | 0.34 | 0.00 | 1.00 |
| 1.marital#2.category | 0.49 | 0.49 | 0.00 | 1.00 |
| 1.marital#2.race | 0.09 | 0.09 | 0.00 | 1.00 |
| 1.marital#2.radiation | 0.39 | 0.39 | 0.10 | 0.98 |
| 1.marital#2.surgery | 0.01 | 0.01 | 0.40 | 0.88 |
| 1.marital#3.category | 0.09 | 0.09 | 0.00 | 1.00 |
| 1.marital#3.race | 0.04 | 0.04 | 0.00 | 1.00 |
| 1.marital#3.seerstage | 0.01 | 0.01 | 0.30 | 0.87 |
| 1.marital#4.race | 0.04 | 0.04 | 0.00 | 1.00 |
| 1.marital#4.seerstage | 0.10 | 0.10 | 0.10 | 0.97 |
| 1.marital#c.age | 47.78 | 47.78 | 0.00 | 1.00 |
| 1.radiation | 0.44 | 0.44 | 0.20 | 0.94 |
| 1.radiation#2.category | 0.31 | 0.30 | 0.20 | 0.93 |
| 1.radiation#3.category | 0.04 | 0.04 | 0.00 | 1.00 |
| 1.radiation#3.seerstage | 0.00 | 0.00 | 0.80 | 0.71 |
| 1.radiation#4.seerstage | 0.05 | 0.05 | 0.10 | 0.95 |
| 1.surgery | 0.44 | 0.44 | 0.10 | 0.98 |
| 1.surgery#1.radiation | 0.05 | 0.05 | 0.00 | 1.00 |
| 1.surgery#2.category | 0.29 | 0.29 | 0.10 | 0.98 |
| 1.surgery#2.radiation | 0.38 | 0.38 | 0.00 | 1.00 |
| 1.surgery#3.category | 0.08 | 0.08 | 0.00 | 1.00 |
| 1.surgery#4.seerstage | 0.08 | 0.08 | 0.00 | 1.00 |
| 1.surgery#c.age | 28.07 | 28.09 | 0.10 | 0.98 |
| 2.category | 0.68 | 0.68 | 0.00 | 1.00 |
| 2.category#3.seerstage | 0.01 | 0.01 | 0.60 | 0.75 |
| 2.category#4.seerstage | 0.01 | 0.01 | 0.30 | 0.91 |
| 2.category#c.age | 45.12 | 45.11 | 0.00 | 0.99 |
| 2.marital | 0.08 | 0.08 | 0.10 | 0.96 |
| 2.marital#1.radiation | 0.03 | 0.03 | 0.20 | 0.94 |
| 2.marital#1.surgery | 0.01 | 0.01 | 0.00 | 1.00 |
| 2.marital#2.category | 0.05 | 0.05 | 0.20 | 0.95 |
| 2.marital#2.race | 0.02 | 0.02 | 0.30 | 0.92 |
| 2.marital#2.radiation | 0.06 | 0.06 | 0.10 | 0.96 |
| 2.marital#2.surgery | 0.00 | 0.00 | 0.50 | 0.83 |
| 2.marital#3.category | 0.01 | 0.01 | 0.00 | 1.00 |
| 2.marital#3.race | 0.00 | 0.00 | 0.00 | 1.00 |
| 2.marital#3.seerstage | 0.00 | 0.00 | 0.80 | 0.71 |
| 2.marital#4.race | 0.01 | 0.01 | 0.00 | 1.00 |
| 2.marital#4.seerstage | 0.01 | 0.01 | 0.00 | 1.00 |
| 2.marital#c.age | 5.61 | 5.63 | 0.10 | 0.97 |
| 2.race | 0.16 | 0.16 | 0.20 | 0.95 |
| 2.race#1.radiation | 0.07 | 0.07 | 0.00 | 1.00 |
| 2.race#1.surgery | 0.07 | 0.07 | 0.10 | 0.96 |
| 2.race#2.category | 0.11 | 0.11 | 0.20 | 0.94 |
| 2.race#2.radiation | 0.09 | 0.09 | 0.00 | 1.00 |
| 2.race#2.surgery | 0.00 | 0.00 | 0.00 | 1.00 |
| 2.race#3.category | 0.01 | 0.01 | 0.00 | 1.00 |
| 2.race#3.seerstage | 0.00 | 0.00 | 0.00 | 1.00 |
| 2.race#4.seerstage | 0.02 | 0.02 | 0.00 | 1.00 |
| 2.race#c.age | 10.26 | 10.30 | 0.20 | 0.95 |
| 2.radiation | 0.55 | 0.56 | 0.10 | 0.96 |
| 2.radiation#2.category | 0.37 | 0.37 | 0.10 | 0.96 |
| 2.radiation#3.category | 0.07 | 0.07 | 0.00 | 1.00 |
| 2.radiation#3.seerstage | 0.01 | 0.01 | 0.30 | 0.86 |
| 2.radiation#4.seerstage | 0.09 | 0.09 | 0.00 | 1.00 |
| 2.surgery | 0.01 | 0.01 | 0.00 | 1.00 |
| 2.surgery#1.radiation | 0.00 | 0.00 | 0.00 | 1.00 |
| 2.surgery#2.category | 0.01 | 0.01 | 0.00 | 1.00 |
| 2.surgery#2.radiation | 0.01 | 0.01 | 0.00 | 1.00 |
| 2.surgery#3.category | 0.00 | 0.00 | 0.00 | 1.00 |
| 2.surgery#3.seerstage | 0.00 | 0.00 | 0.00 | 1.00 |
| 2.surgery#4.seerstage | 0.00 | 0.00 | 0.00 | 1.00 |
| 2.surgery#c.age | 0.71 | 0.72 | 0.00 | 0.99 |
| 3.category | 0.11 | 0.11 | 0.00 | 1.00 |
| 3.category#4.seerstage | 0.11 | 0.11 | 0.00 | 1.00 |
| 3.category#c.age | 7.48 | 7.48 | 0.00 | 1.00 |
| 3.race | 0.05 | 0.05 | 0.10 | 0.95 |
| 3.race#1.radiation | 0.02 | 0.02 | 0.70 | 0.80 |
| 3.race#1.surgery | 0.02 | 0.02 | 0.00 | 1.00 |
| 3.race#2.category | 0.04 | 0.04 | 0.20 | 0.95 |
| 3.race#2.radiation | 0.03 | 0.03 | 0.40 | 0.88 |
| 3.race#2.surgery | 0.00 | 0.00 | 1.40 | 0.56 |
| 3.race#3.category | 0.00 | 0.00 | 0.00 | 1.00 |
| 3.race#3.seerstage | 0.00 | 0.00 | 0.00 | 1.00 |
| 3.race#4.seerstage | 0.00 | 0.00 | 0.40 | 0.85 |
| 3.race#c.age | 3.55 | 3.53 | 0.20 | 0.95 |
| 3.seerstage | 0.01 | 0.01 | 0.40 | 0.80 |
| 3.seerstage#c.age | 0.67 | 0.63 | 0.40 | 0.79 |
| 4.race | 0.06 | 0.06 | 0.00 | 1.00 |
| 4.race#1.radiation | 0.02 | 0.02 | 0.00 | 1.00 |
| 4.race#1.surgery | 0.02 | 0.02 | 0.00 | 1.00 |
| 4.race#2.category | 0.04 | 0.04 | 0.00 | 1.00 |
| 4.race#2.radiation | 0.03 | 0.03 | 0.00 | 1.00 |
| 4.race#2.surgery | 0.00 | 0.00 | 0.00 | 1.00 |
| 4.race#3.category | 0.00 | 0.00 | 0.00 | 1.00 |
| 4.race#3.seerstage | 0.00 | 0.00 | 0.00 | 1.00 |
| 4.race#4.seerstage | 0.01 | 0.01 | 0.00 | 1.00 |
| 4.race#c.age | 3.81 | 3.81 | 0.00 | 1.00 |
| 4.seerstage | 0.13 | 0.13 | 0.10 | 0.97 |
| 4.seerstage#c.age | 8.80 | 8.78 | 0.10 | 0.97 |
| age | 66.63 | 66.62 | 0.10 | 0.97 |
| c.age#c.age | 4493.40 | 4492.50 | 0.10 | 0.97 |
